# The association of hyponatremia and clinical outcomes in patients with acute myocardial infarction: a cross-sectional study

**DOI:** 10.1186/s12872-022-02700-y

**Published:** 2022-06-18

**Authors:** Andres Cordova Sanchez, Kunal Bhuta, Gary Shmorgon, Nicholas Angeloni, Ryan Murphy, Debanik Chaudhuri

**Affiliations:** 1 Department of Medicine, SUNY Upstate Medical University, 750 East Adams Street, Syracuse, NY 13210 USA; 2 College of Medicine, SUNY Upstate Medical University, Syracuse, NY 13210 USA; 3 Division of Cardiology, SUNY Upstate Medical University, Syracuse, NY 13210 USA

**Keywords:** Hyponatremia, Acute myocardial infarction, Heart failure, Mortality

## Abstract

**Introduction:**

Hyponatremia is a common electrolyte abnormality that has been associated with poor outcomes in several conditions including acute myocardial infarction (AMI). However, those studies were performed in the era before percutaneous coronary intervention (PCI), focused mostly on ST-elevation myocardial infarction (STEMI), and sodium levels up to 72 h of admission. The purpose of this study was to identify the association between hyponatremia and clinical outcomes in patients with acute myocardial infarction.

**Methods:**

We performed a retrospective analysis of patients with a diagnosis of non-ST-elevation myocardial infarction (NSTEMI) and STEMI presenting at our institution from March 2021 to September 2021. Our independent variables were sodium levels on the day of admission and up to 7 days later. Dependent variables were in-hospital mortality, 30-day mortality, length of hospital stay, intensive care admission, new heart failure diagnosis, and ejection fraction.

**Results:**

50.2% of patients had hyponatremia up to 7 days of admission. Intensive care admission was higher in patients with hyponatremia up to7 days (69.7% vs 54.3%, P 0.019, OR 1.9), they had worse 30-day mortality (12.7% vs to 2.2%, P 0.004, OR 6.5), in hospital mortality (9.9% vs 1.1%, P 0.006, OR 9.9), and new heart failure diagnosis (31.5% vs 17.9%, P < 0.043, OR 2.1). Hyponatremia on admission was associated with in-hopital mortality (16.3% vs 3.8%, P 0.004, OR 4.9), 30-day mortality (18.4% vs 5.9%, P 0.017, OR 3.5).

**Conclusions:**

This study suggests that hyponatremia on admission and at any point during the first seven days of hospitalization are associated with in-hospital and 30-day mortality.

**Supplementary Information:**

The online version contains supplementary material available at 10.1186/s12872-022-02700-y.

## Introduction

Approximately 805,000 people in the United States have an event of AMI every year [1]. In the past, studies have attempted to assess various prognosticating factors in this patient population. Dysnatremias especially hyponatremia is regarded as the most common electrolyte abnormality seen in the hospital [2] and is a useful marker of water excess in the body [3]. Hyponatremia has been associated with increased mortality in patients with several cardiovascular conditions including heart failure [4–6].

Although the association between hyponatremia in patients with AMI with mortality and heart failure has been previously described [7–10]; most of the studies were focused on hyponatremia up to 72 h of admission, patients with STEMI, and were performed in the era before PCI. The cause for hyponatremia in patients with AMI has not been elucidated. It is likely a multifactorial phenomenon involving the sympathetic system, renin–angiotensin–aldosterone cascade, and vasopressin release [11, 12]. The effect of hyponatremia on cardiac function in this patient population remains unclear. It is hypothesized that low sodium levels may lead to downregulation of calcium channels in the myocyte leading to decreased contractility [13].

The purpose of this study was to identify the association between hyponatremia and clinical outcomes in patients with acute myocardial infarction.

## Methods

### Study design

We performed a single-center, cross-sectional, observational study of patients presenting to Upstate Medical University (Syracuse, NY). All patient hospital data was obtained utilizing the Slicer Dicer tool from EPIC^®^. This study was determined to be exempt from Institutional Review Board (IRB) by the Upstate Medical university IRB (ID: 1825224-1).

### Study population and criteria for inclusion and exclusion

We included all patients 18 to 89 years old with ICD-10 and ICD-9 corresponding to STEMI or NSTEMI diagnosis from March 2021 to September 2021 at Upstate Medical University. Upon electronic medical record revision, the diagnosis of STEMI or NSTEMI was confirmed. Patients were excluded if there was no sodium level on admission, they were younger than 18 years old, and if they were older than 89 years old. The sodium level was corrected for hyperglycemia using the formula proposed by Hillier et al., corrected sodium = [Measured sodium + 2.4 * (Serum glucose − 100)]/100 [14].

### Independent and dependent variable assessment

We aimed to identify the association between hyponatremia present on admission and up to 7 days of hospitalization in patients diagnosed with STEMI and NSTEMI to new heart failure development, length of hospital stay, intensive care admission, depressed ejection fraction, in-hospital mortality, and 30-day mortality.

Sodium levels included in the study were the first sodium level drawn at the hospital and the lowest sodium level during the first 7 days of hospitalization. The calculated ejection fraction from echocardiograms done during hospitalization was used, in case more than one echocardiogram had been performed the last measurement was used.

The independent variables were hyponatremia on admission (defined as sodium level < 135 mmol/L during the first blood test drawn in our hospital) and hyponatremia (sodium level < 135 mmol/L) present at any point during the first seven days of hospitalization. Delta sodium (∆Na) was defined as the difference between sodium on admission and the lowest sodium up to 7 days of hospitalization.

Our dependent variables were in-hospital mortality, 30-day mortality, length of hospital stay, intensive care admission, new heart failure diagnosis, and ejection fraction. 30-day-all-cause-mortality was defined as death for any reason up to 30 days from the moment of hospital admission.

### Baseline covariates

We collected and analyzed age, smoking status, sex, past medical history including hypertension, diabetes, transient ischemic attack, peripheral vascular disease, cancer, heart failure, myocardial infarction, chronic kidney disease, previous percutaneous coronary intervention, and coronary artery bypass graft, as well as the use of diuretics ACEi, ARB, or ARNi to address and minimize confounding effects and frailty bias.

### Statistical analysis

For this study, we present categorical variables as percentages and continuous variables as means (SD) for normally distributed data and median with interquartile range (IQR) for non-normally distributed data. A P value of ≤ 0.05 was considered statistically significant.

Baseline characteristics were analyzed using descriptive statistics. The statistical tools used to analyze categorical data were Fisher’s exact test, odds ratio (95% confidence interval (CI)), and logistic regression analysis with backward elimination. A P value of ≤ 0.05 was considered statistically significant. For continuous data, the Kolmogorov–Smirnov test was done to determine normality. If the data was found to be normally distributed, a t-test was utilized to determine if there was a significant P-value. For non-normally distributed data, Mann–Whitney U-test was done to evaluate for a significant P-value.

The variables initially entered for multivariate logistic regression analysis were age, sex, smoking status, past medical history including hypertension, diabetes, transient ischemic attack, peripheral vascular disease, cancer, heart failure, myocardial infarction, chronic kidney disease, previous percutaneous coronary intervention, and coronary artery bypass graft, as well as the use of diuretics, ACEi, ARB, or ARNi. All statistical analysis was performed using the IBM^®^ SPSS^®^ software version 28.0.

## Results

### Baseline characteristics

A total of 234 patients were included, of which 44.4% were females. The mean age was 64.86 years. 20.9% of the patients had hyponatremia on admission. Of the 185 patients who did not have hyponatremia on admission, 50.2% developed hyponatremia at some point during the first seven days of hospitalization. Baseline characteristics grouped by the presence of hyponatremia can be seen in Tables 1 and 2.

**Table 1:** Baseline characteristics of categorical data

	Hyponatremia during the first seven days of admission						
	Total		Absent		Present		P-value
	Number	Percentage (%)	Number	Percentage (%)	Number	Percentage (%)	
N	234		92	39.30	142	60.70	
Females	104	44.40	44	47.80	60	42.30	0.422
Current smoker	56	23.9	16	17.40	40	28.20	0.062
Hypertension	189	80.8	73	79.30	116	81.70	0.735
Diabetes mellitus	104	44.4	41	44.60	63	44.40	1
Stroke	22	9.4	9	9.80	13	9.20	1
Transient ischemic attack	2	0.9	1	1.10	1	0.70	1
PVD	22	9.4	10	10.90	12	8.50	0.647
Cancer	54	23.1	17	18.50	37	26.10	0.206
Previous MI	53	22.6	24	26.10	29	20.40	0.34
History of HF	46	19.7	14	15.20	32	22.50	0.182
Previous PCI	68	29.1	27	29.30	41	28.90	1
Previous CABG	31	13.2	8	8.70	23	16.20	0.116
History of CKD	48	20.5	17	18.50	31	21.80	0.62
Taking ACEi/ARB/ARNi	81	34.6	33	35.90	48	33.80	0.779
Outpatient diuretic use	70	29.9	29	31.50	41	28.90	0.664
In-patient diuretic use	125	53.4	44	47.8	81	57	0.182
Diagnosis of NSTEMI type I	176	75.2	73	79.30	103	72.50	0.279
Diagnosis of STEMI	58	24.8	19	20.70	39	27.50	0.279
ICU admission	149	63.7	50	54.30	99	69.70	0.019
Cardiac catheterization	173	73.9	65	70.70	108	76.10	0.365
Stents placed	100	42.7	36	39.10	64	45.10	0.418
CABG done	15	6.4	4	4.30	11	7.70	0.415
EF < 50%	94	40.2	24	28.90	70	50.70	0.002
New heart failure diagnosis	49	20.9	14	17.9	35	31.5	0.043
30-day Mortality	20	8.5	2	2.20	18	12.70	0.004
In-hospital mortality	15	6.4	1	1.10	14	9.90	0.006

N = number, PVD = peripheral vascular disease, MI = Myocardial infarction, HF = heart failure, PCI = percutaneous coronary intervention, CABG = coronary artery bypass graft, CKD = chronic kidney disease, ACEi = acetylcholinesterase inhibitor, ARB = aldosterone receptor blocker, ARNi = angiotensin-neprylisin inhibitor, EF = ejection fraction, ICU = intensive care unit

**Table 2:** Baseline characteristics of continuous data

	N	Mean	SD
Age*	234	64.86	14.51

	N	Median	IQR
Sodium level on admission in mmol/L^†^	234	137	135–140
Lowest Na level up to 7 days of admission in mmol/L^†^	234	133	130–136
EF% during admission^†^	221	52	41.5–58
Days of mortality after admission^†^	13	10	3.5–16.5
Length of hospital stay in days^†^	234	4	2–8

EF = ejection fraction, Na = sodium. SD = standard deviation, N = number, Na = sodium, EF = ejection fraction. SD = Standard Deviation, IQR = interquartile range

*Data is normally distributed

^†^Data is non-normally distributed

Hyponatremia was present on admission in 20.9% of the patients and 60.7% had hyponatremia during the first seven days of hospitalization. The median sodium level on admission was 137 mmol/L (IQR 135–140 mmol/L). The median sodium nadir during the first seven days of hospitalization was 133 mmol/L (IQR 130–136 mmol/L) (Table 2).

There was no significant difference for most of the baseline characteristics between patients presenting hyponatremia versus those who did not, except for patients with a history of diabetes mellitus (Table 1).

### Outcomes

The median length of hospital stay was 4 days. Patients with hyponatremia on admission had a similar length of hospital stay compared to those without and were found to be similar on non-parametric tests (Table 3). In contrast, patients who had hyponatremia during the first seven days of hospitalization had a median length of hospital stay of 3 days (IQR 2–5) compared to 5 days (IQR 2–10) in the normonatremic group (Table 4).

**Table 3:** Comparison of continuous data in patients with hyponatremia on admission

	Hyponatremia on admission		
	Absent	Present	P-value
Age, mean (SD)*	65.14(14.504)	63.82(14.634)	0.629
EF% during Admission, median (IQR)^†^	52(44.5–59)	43(34–55)	< 0.001
Length of Hospital Stay, median (IQR)^†^	4(2–9)	4(2–8)	0.468
GFR, median (IQR)^†^	79(53–90)	86(62–90)	0.216
Regular troponin, median (IQR)^†^	0.645(0.15–2.52)	0.66(0.1–1.56)	0.492
High sensitivity troponin, median (IQR)^†^	339(141–738)	321(91–1963)	0.720

For normally distributed data mean and SD are presented. For non-normally-distributed data median and IQR are presented

Na = sodium, EF = ejection fraction, GFR = glomerular filtration range, SD = Standard Deviation, IQR = interquartile range

*Data is normally distributed

^†^Data is non-normally distributed

**Table 4:** Comparison of continuous data in patients with hyponatremia during the first 7 days of hospitalization

	Hyponatremia during the first 7 days of hospitalization		
	Absent	Present	P-value
Age, mean (SD)*	64.75(15.671)	64.94(13.763)	0.091
EF% during Admission, median (IQR)^†^	55(48–60)	48(40–57)	0.009
Length of Hospital Stay, median (IQR)^†^	3(2–5)	5(2–10)	< 0.001
GFR, median (IQR)^†^	81.5(62.5–90)	82(50–90)	0.568
Regular troponin, median (IQR)^†^	0.58(0.06–1.8)	0.76(0.23–1.98)	0.178
High sensitivity troponin, median (IQR)^†^	287(102.5–556.75)	398(150–1434)	0.125

For normally distributed data mean and SD are presented. For non-normally-distributed data median and IQR are presented

Na = sodium, EF = ejection fraction, GFR = glomerular filtration range, SD = Standard Deviation, IQR = interquartile range

*Data is normally distributed

^†^Data is non-normally distributed

In-hospital mortality was seen in 6.4% and 30-day mortality in 8.5% of our patients. In bivariate analysis, in-hospital mortality was seen in 9.9% compared to 1.1%, OR 9.95 (P 0.006) in those who had hyponatremia during the first 7 days of admission versus those without. Patients who had hyponatremia on admission also had a significant association with in-hospital mortality of 16.3% versus 3.78%, OR 4.9 (P 0.004) (Table 6).

30-day mortality was higher in patients with hyponatremia during the first 7 days of admission 12.7% versus 2.2%, OR 6.5 (P 0.004) (Table 5). In-hospital mortality was also associated with hyponatremia on admission 16.3% versus 3.8%, OR 3.5 (P 0.004) (Table 6).

**Table 5:** Outcomes in patients with hyponatremia during the first 7 days of hospitalization

	Hyponatremia during the first 7 days of admission			
	Absent	Present	P-value	OR(95% CI)
In-hospital mortality	1.1%(1)	9.9%(14)	0.006	9.953(1.286–77.043)*
30-day mortality	2.2%(2)	12.7%(18)	0.004	6.532(1.478–28.865)*
ICU or CCU admission	54.3%(50)	69.7%(99)	0.019	1.934(1.122–3.333)*
New heart failure diagnosis	17.9%(14)	31.5%(35)	0.043	2.105(1.042–4.254)*
EF < 50%	28.9%(24)	50.7%(70)	0.002	2.531(1.417–4.52)*

ICU = intensive care unit, CCU = cardiac care unit, OR = odds ratio

*Significant after regression analysis

**Table 6:** Outcomes in patients with hyponatremia on admission

	Hyponatremia on admission			
	Absent	Present	P-value	OR(95% CI)
In-hospital mortality	3.8%(7)	16.3%(8)	0.004	4.962(1.702–14.61)*
30-day mortality	5.9%(11)	18.4%(9)	0.017	3.559(1.3183–9.162)*
ICU or CCU admission	65.4%(121)	57.1%(28)	0.318	0.705(0.371–1.340)
New heart failure diagnosis	24%(36)	33.3%(13)	0.305	1.583(0.738–3.399)
EF < 50%	37.6%(65)	60.4%(29)	0.005	2.536(1.317–4.883)*

ICU = intensive care unit, CCU = cardiac care unit, OR = odds ratio

*Significant after regression analysis

Both hyponatremia on admission and during the first 7 days of admission was significantly associated with mortality even when depressed ejection fraction and heart failure diagnosis were included in logistic regression analysis.

Intensive care admission was higher in patients with hyponatremia up to 7 days of admission (69.7% vs 54.3%, OR 1.9, P 0.019). This association was not seen in patients with hyponatremia on admission only.

New heart failure diagnosis was not significantly associated with hyponatremia on admission. However, it was associated with patients that had hyponatremia during the first 7 days of hospitalization (31.5%. vs 17.9%, OR 2.1, P 0.043). Depressed ejection fraction was higher in those with hyponatremia during the first 7 days of hospitalization (50.7% vs 28.9%, P 0.002, OR 2.531) (Table 5). Both hyponatremia on admission and during the first seven days of hospitalization were significantly associated with lower left ventricular ejection fraction (Tables 3 and 4).

The median ∆Na was 3 with an IQR of 0 to 7. We did not find an association between ∆Na and any of the studied outcomes.

## Discussion

Hyponatremia during the first seven days of hospitalization was seen in more than half the patients with AMI and was associated with poorer outcomes including 30-day and in-hospital mortality, ICU or CCU admission, new heart failure diagnosis, and depressed EF. Hyponatremia on admission was also associated with poorer outcomes. However, there was no significant association between ICU or CCU admission as well as new heart failure diagnosis.

Several studies have shown an association between hyponatremia up to 72 h of admission with short- and long-term mortality in patients with AMI [7, 8, 15]. More recently there has been conflicting evidence in patients with STEMI receiving percutaneous coronary intervention prompting the hypothesis that hyponatremia is a marker of disease severity and not an independent predictor of mortality [9]. This conflicting evidence could be, at least in part, explained by advances in PCI and medical therapy. Although less studied, NSTEMI also has been associated with short-term mortality [8, 15], but since these studies were performed before the PCI era. We found a significant association between hyponatremia on admission and up to 7 days of hospitalization with in-hospital and 30-day mortality. Although previous studies have demonstrated that hyponatremia conveys a worse prognosis in patients with heart failure [5, 6]. In those patients, correction of hyponatremia fails to improve mortality [6]. In patients with AMI, correction of hyponatremia might not have any mortality benefits as well [8].

Hyponatremia for up to 7 days was associated with new heart failure diagnosis. Other studies have reported an association between STEMI and heart failure in up to 30.2% of the patients [7, 10]. Qureshi et al. reported a higher prevalence of heart failure in patients with STEMI and NSTEMI that had persistent hyponatremia (6.4% and 14.3%) and hyponatremia only on admission (5.2% and 3.8%) compared to those without hyponatremia (1.8% and 2.1%). They found that patients with hyponatremia on admission had an increased risk of 30-day rehospitalization due to heart failure regardless of whether the sodium was corrected or not, it is important to highlight that in this study, 20.4% of patients underwent revascularization [8], compared to 42.7% in ours. In our study, hyponatremia on admission was not associated with heart failure, but it was significantly associated with hyponatremia up to 7 days of admission. A possible explanation for this finding is that hyponatremia might take some time to develop in patients with new-onset or acutely decompensated heart failure.

Although patients with hyponatremia appeared to have a more severe condition as evidenced by the higher admissions to ICU or CCU and lower EF% on echocardiograms. We did not find a statistically significant association with mortality between the levels of peak troponin and hyponatremia; our study may be underpowered to find this association. Further studies would be needed to discover if hyponatremia is also related to the size of myocardial involvement in patients with AMI.

The association between hyponatremia and mortality was significant even after accounting for depressed ejection fraction, heart failure diagnosis, and in-patient use of diuretics in the regression analysis. This could indicate that hyponatremia in patients with AMI is more than only a marker of heart failure in these patients.

## Limitations of the study

This was a retrospective, single site, observational analysis that had a relatively small number of patients, thus it may have been underpowered to find certain differences. There might be other confounding factors that we did not take into consideration. Other causes of hyponatremia and BNP or NT-ProBNP levels might have a significant association with poorer outcomes, but this was not analyzed in the study.

## Conclusions

This study suggests that hyponatremia on admission and at any point during the first seven days of hospitalization are associated with in-hospital and 30-day mortality.

## Supplementary Information

**Additional file 1.** Regression and sensitivity analysis.

**Additional file 2.** Regression and sensitivity analysis cont'd.

**Additional file 3.** Data.

## Data Availability

All data generated or analyzed during this study are included in this published article and its supplementary information files.
