# Supplementary material for: The association of hyponatremia and clinical outcomes in patients with acute myocardial infarction: a cross-sectional study
Source: BMC Cardiovasc Disord. 2022 Jun 18;22:276. doi: 10.1186/s12872-022-02700-y (PMC9206366; doi:10.1186/s12872-022-02700-y)
Supplement: Supplementary file 1 — Additional file 1. Regression and sensitivity analysis. [file 12872_2022_2700_MOESM1_ESM.docx]

**Dependent variable: In-hospital mortality**

**Independent variable: Hyponatremia on admission**

| **Omnibus Tests of Model Coefficients** | | | | |
| --- | --- | --- | --- | --- |
|  | | Chi-square | df | Sig. |
| Step 1 | Step | 32.014 | 20 | .043 |
|  | Block | 32.014 | 20 | .043 |
|  | Model | 32.014 | 20 | .043 |
| Step 2^a^ | Step | -.002 | 1 | .960 |
|  | Block | 32.012 | 19 | .031 |
|  | Model | 32.012 | 19 | .031 |
| Step 3^a^ | Step | -.025 | 1 | .875 |
|  | Block | 31.987 | 18 | .022 |
|  | Model | 31.987 | 18 | .022 |
| Step 4^a^ | Step | -.041 | 1 | .839 |
|  | Block | 31.946 | 17 | .015 |
|  | Model | 31.946 | 17 | .015 |
| Step 5^a^ | Step | -.093 | 1 | .761 |
|  | Block | 31.853 | 16 | .010 |
|  | Model | 31.853 | 16 | .010 |
| Step 6^a^ | Step | -.672 | 2 | .714 |
|  | Block | 31.181 | 14 | .005 |
|  | Model | 31.181 | 15 | .008 |
| Step 7^a^ | Step | -.355 | 1 | .551 |
|  | Block | 30.826 | 13 | .004 |
|  | Model | 30.826 | 13 | .004 |
| Step 8^a^ | Step | -.400 | 1 | .527 |
|  | Block | 30.426 | 12 | .002 |
|  | Model | 30.426 | 12 | .002 |
| Step 9^a^ | Step | -.653 | 1 | .419 |
|  | Block | 29.773 | 11 | .002 |
|  | Model | 29.773 | 11 | .002 |
| Step 10^a^ | Step | -.886 | 1 | .346 |
|  | Block | 28.887 | 10 | .001 |
|  | Model | 28.887 | 10 | .001 |
| Step 11^a^ | Step | -.908 | 1 | .341 |
|  | Block | 27.979 | 9 | <.001 |
|  | Model | 27.979 | 9 | <.001 |
| Step 12^a^ | Step | -.849 | 1 | .357 |
|  | Block | 27.129 | 8 | <.001 |
|  | Model | 27.129 | 8 | <.001 |
| Step 13^a^ | Step | -.388 | 1 | .533 |
|  | Block | 26.741 | 7 | <.001 |
|  | Model | 26.741 | 7 | <.001 |
| Step 14^a^ | Step | -1.305 | 1 | .253 |
|  | Block | 25.436 | 6 | <.001 |
|  | Model | 25.436 | 6 | <.001 |
| Step 15^a^ | Step | -2.116 | 1 | .146 |
|  | Block | 23.320 | 5 | <.001 |
|  | Model | 23.320 | 5 | <.001 |
| Step 16^a^ | Step | -2.506 | 1 | .113 |
|  | Block | 20.813 | 4 | <.001 |
|  | Model | 20.813 | 4 | <.001 |
| Step 17^a^ | Step | -2.701 | 1 | .100 |
|  | Block | 18.112 | 3 | <.001 |
|  | Model | 18.112 | 3 | <.001 |
| a. A negative Chi-squares value indicates that the Chi-squares value has decreased from the previous step. | | | | |

| **Hosmer and Lemeshow Test** | | | |
| --- | --- | --- | --- |
| Step | Chi-square | df | Sig. |
| 1 | 3.228 | 8 | .919 |
| 2 | 3.210 | 8 | .920 |
| 3 | 3.501 | 8 | .899 |
| 4 | 3.331 | 8 | .912 |
| 5 | 4.941 | 8 | .764 |
| 6 | 3.270 | 8 | .916 |
| 7 | 6.693 | 8 | .570 |
| 8 | 5.554 | 8 | .697 |
| 9 | 3.386 | 8 | .908 |
| 10 | 3.848 | 8 | .871 |
| 11 | 4.401 | 8 | .819 |
| 12 | 1.905 | 8 | .984 |
| 13 | 1.708 | 8 | .989 |
| 14 | 3.067 | 8 | .930 |
| 15 | 2.248 | 7 | .945 |
| 16 | 6.449 | 7 | .488 |
| 17 | 3.230 | 4 | .520 |

| **Classification Table**^a^ | | | | | |
| --- | --- | --- | --- | --- | --- |
|  | Observed | | Predicted | | |
|  |  |  | In Hospital mortality Yes:1 No:0 | | Percentage Correct |
|  |  |  | no | yes |  |
| Step 1 | In Hospital mortality Yes:1 No:0 | no | 204 | 2 | 99.0 |
|  |  | yes | 13 | 2 | 13.3 |
|  | Overall Percentage | |  |  | 93.2 |
| Step 2 | In Hospital mortality Yes:1 No:0 | no | 204 | 2 | 99.0 |
|  |  | yes | 13 | 2 | 13.3 |
|  | Overall Percentage | |  |  | 93.2 |
| Step 3 | In Hospital mortality Yes:1 No:0 | no | 204 | 2 | 99.0 |
|  |  | yes | 13 | 2 | 13.3 |
|  | Overall Percentage | |  |  | 93.2 |
| Step 4 | In Hospital mortality Yes:1 No:0 | no | 204 | 2 | 99.0 |
|  |  | yes | 13 | 2 | 13.3 |
|  | Overall Percentage | |  |  | 93.2 |
| Step 5 | In Hospital mortality Yes:1 No:0 | no | 204 | 2 | 99.0 |
|  |  | yes | 13 | 2 | 13.3 |
|  | Overall Percentage | |  |  | 93.2 |
| Step 6 | In Hospital mortality Yes:1 No:0 | no | 205 | 1 | 99.5 |
|  |  | yes | 13 | 2 | 13.3 |
|  | Overall Percentage | |  |  | 93.7 |
| Step 7 | In Hospital mortality Yes:1 No:0 | no | 204 | 2 | 99.0 |
|  |  | yes | 13 | 2 | 13.3 |
|  | Overall Percentage | |  |  | 93.2 |
| Step 8 | In Hospital mortality Yes:1 No:0 | no | 205 | 1 | 99.5 |
|  |  | yes | 13 | 2 | 13.3 |
|  | Overall Percentage | |  |  | 93.7 |
| Step 9 | In Hospital mortality Yes:1 No:0 | no | 204 | 2 | 99.0 |
|  |  | yes | 13 | 2 | 13.3 |
|  | Overall Percentage | |  |  | 93.2 |
| Step 10 | In Hospital mortality Yes:1 No:0 | no | 204 | 2 | 99.0 |
|  |  | yes | 14 | 1 | 6.7 |
|  | Overall Percentage | |  |  | 92.8 |
| Step 11 | In Hospital mortality Yes:1 No:0 | no | 204 | 2 | 99.0 |
|  |  | yes | 14 | 1 | 6.7 |
|  | Overall Percentage | |  |  | 92.8 |
| Step 12 | In Hospital mortality Yes:1 No:0 | no | 205 | 1 | 99.5 |
|  |  | yes | 14 | 1 | 6.7 |
|  | Overall Percentage | |  |  | 93.2 |
| Step 13 | In Hospital mortality Yes:1 No:0 | no | 205 | 1 | 99.5 |
|  |  | yes | 14 | 1 | 6.7 |
|  | Overall Percentage | |  |  | 93.2 |
| Step 14 | In Hospital mortality Yes:1 No:0 | no | 203 | 3 | 98.5 |
|  |  | yes | 12 | 3 | 20.0 |
|  | Overall Percentage | |  |  | 93.2 |
| Step 15 | In Hospital mortality Yes:1 No:0 | no | 206 | 0 | 100.0 |
|  |  | yes | 15 | 0 | .0 |
|  | Overall Percentage | |  |  | 93.2 |
| Step 16 | In Hospital mortality Yes:1 No:0 | no | 206 | 0 | 100.0 |
|  |  | yes | 15 | 0 | .0 |
|  | Overall Percentage | |  |  | 93.2 |
| Step 17 | In Hospital mortality Yes:1 No:0 | no | 206 | 0 | 100.0 |
|  |  | yes | 15 | 0 | .0 |
|  | Overall Percentage | |  |  | 93.2 |
| a. The cut value is .500 | | | | | |

| **Variables in the Equation** | | | | | | | | | |
| --- | --- | --- | --- | --- | --- | --- | --- | --- | --- |
|  | | B | S.E. | Wald | df | Sig. | Exp(B) | 95% C.I.for EXP(B) | |
|  |  |  |  |  |  |  |  | Lower | Upper |
| Step 1^a^ | SEX Female: 1 Male:0(1) | .571 | .669 | .730 | 1 | .393 | 1.771 | .477 | 6.569 |
|  | Age 1<65 2:≥ 65(1) | .722 | .822 | .771 | 1 | .380 | 2.059 | .411 | 10.319 |
|  | Current Smoker Yes:1 No: 0(1) | -2.383 | 1.216 | 3.841 | 1 | .050 | .092 | .009 | 1.000 |
|  | History of HTN Yes:1 No:0(1) | .050 | 1.005 | .002 | 1 | .961 | 1.051 | .147 | 7.531 |
|  | History of Diabetes Yes:1 No:0(1) | -.559 | .757 | .546 | 1 | .460 | .572 | .130 | 2.520 |
|  | History of Stroke Yes:1 No:0(1) | 2.163 | 1.121 | 3.723 | 1 | .054 | 8.701 | .966 | 78.354 |
|  | History of Transient Ischemic Attack Yes:1 No:0(1) | -16.353 | 25446.454 | .000 | 1 | .999 | .000 | .000 | . |
|  | History of Peripheral Vascular Disease Yes:1 No:0(1) | .361 | 1.185 | .093 | 1 | .760 | 1.435 | .141 | 14.646 |
|  | History of Cancer Yes:1 No:0(1) | -.379 | .746 | .258 | 1 | .612 | .685 | .159 | 2.953 |
|  | History of Myocardial Infarction Yes:1 No:0(1) | -.184 | 1.181 | .024 | 1 | .876 | .832 | .082 | 8.426 |
|  | History of Percutaneous Coronary Intervention Yes:1 No:0(1) | -.965 | 1.182 | .667 | 1 | .414 | .381 | .038 | 3.863 |
|  | History of CABG Yes:1 No:0(1) | 1.420 | 1.150 | 1.525 | 1 | .217 | 4.136 | .434 | 39.373 |
|  | History of CKD Yes:1 No:0(1) | -.711 | .964 | .545 | 1 | .460 | .491 | .074 | 3.247 |
|  | Taking ACEi, ARNi, or ARB Yes:1 No:0(1) | -1.894 | .935 | 4.099 | 1 | .043 | .150 | .024 | .941 |
|  | Taking Diuretics Yes:1 No:0(1) | .453 | .770 | .346 | 1 | .557 | 1.573 | .348 | 7.113 |
|  | Heart failure diagnosis (0=No HF, 1= History of HF, 2=New HF) |  |  | .603 | 2 | .740 |  |  |  |
|  | Heart failure diagnosis (0=No HF, 1= History of HF, 2=New HF)(1) | .253 | .936 | .073 | 1 | .787 | 1.288 | .206 | 8.061 |
|  | Heart failure diagnosis (0=No HF, 1= History of HF, 2=New HF)(2) | .605 | .779 | .602 | 1 | .438 | 1.831 | .397 | 8.434 |
|  | Left ventricular ejection fraction <50% Yes:1 No:0(1) | 1.334 | .820 | 2.646 | 1 | .104 | 3.797 | .761 | 18.954 |
|  | In patient diuretic use(1) | .893 | .765 | 1.364 | 1 | .243 | 2.443 | .546 | 10.933 |
|  | Hyponatremia on admission Yes:1 No:0(1) | 2.142 | .746 | 8.250 | 1 | .004 | 8.515 | 1.974 | 36.724 |
|  | Constant | -4.810 | 1.295 | 13.798 | 1 | <.001 | .008 |  |  |
| Step 2^a^ | SEX Female: 1 Male:0(1) | .562 | .642 | .767 | 1 | .381 | 1.754 | .499 | 6.169 |
|  | Age 1<65 2:≥ 65(1) | .726 | .818 | .789 | 1 | .374 | 2.068 | .416 | 10.270 |
|  | Current Smoker Yes:1 No: 0(1) | -2.389 | 1.209 | 3.905 | 1 | .048 | .092 | .009 | .981 |
|  | History of Diabetes Yes:1 No:0(1) | -.557 | .755 | .544 | 1 | .461 | .573 | .130 | 2.518 |
|  | History of Stroke Yes:1 No:0(1) | 2.156 | 1.110 | 3.775 | 1 | .052 | 8.637 | .981 | 76.022 |
|  | History of Transient Ischemic Attack Yes:1 No:0(1) | -16.372 | 25443.439 | .000 | 1 | .999 | .000 | .000 | . |
|  | History of Peripheral Vascular Disease Yes:1 No:0(1) | .372 | 1.164 | .102 | 1 | .749 | 1.451 | .148 | 14.200 |
|  | History of Cancer Yes:1 No:0(1) | -.379 | .745 | .259 | 1 | .611 | .685 | .159 | 2.950 |
|  | History of Myocardial Infarction Yes:1 No:0(1) | -.185 | 1.180 | .024 | 1 | .876 | .831 | .082 | 8.394 |
|  | History of Percutaneous Coronary Intervention Yes:1 No:0(1) | -.950 | 1.140 | .694 | 1 | .405 | .387 | .041 | 3.615 |
|  | History of CABG Yes:1 No:0(1) | 1.418 | 1.149 | 1.522 | 1 | .217 | 4.128 | .434 | 39.244 |
|  | History of CKD Yes:1 No:0(1) | -.695 | .902 | .593 | 1 | .441 | .499 | .085 | 2.926 |
|  | Taking ACEi, ARNi, or ARB Yes:1 No:0(1) | -1.883 | .908 | 4.303 | 1 | .038 | .152 | .026 | .901 |
|  | Taking Diuretics Yes:1 No:0(1) | .460 | .755 | .372 | 1 | .542 | 1.585 | .361 | 6.958 |
|  | Heart failure diagnosis (0=No HF, 1= History of HF, 2=New HF) |  |  | .647 | 2 | .724 |  |  |  |
|  | Heart failure diagnosis (0=No HF, 1= History of HF, 2=New HF)(1) | .244 | .916 | .071 | 1 | .790 | 1.276 | .212 | 7.679 |
|  | Heart failure diagnosis (0=No HF, 1= History of HF, 2=New HF)(2) | .612 | .766 | .638 | 1 | .424 | 1.844 | .411 | 8.275 |
|  | Left ventricular ejection fraction <50% Yes:1 No:0(1) | 1.321 | .773 | 2.922 | 1 | .087 | 3.746 | .824 | 17.030 |
|  | In patient diuretic use(1) | .896 | .762 | 1.381 | 1 | .240 | 2.450 | .550 | 10.919 |
|  | Hyponatremia on admission Yes:1 No:0(1) | 2.147 | .739 | 8.436 | 1 | .004 | 8.559 | 2.010 | 36.446 |
|  | Constant | -4.777 | 1.106 | 18.662 | 1 | <.001 | .008 |  |  |
| Step 3^a^ | SEX Female: 1 Male:0(1) | .559 | .640 | .763 | 1 | .382 | 1.749 | .499 | 6.136 |
|  | Age 1<65 2:≥ 65(1) | .745 | .811 | .844 | 1 | .358 | 2.106 | .430 | 10.313 |
|  | Current Smoker Yes:1 No: 0(1) | -2.400 | 1.207 | 3.954 | 1 | .047 | .091 | .009 | .966 |
|  | History of Diabetes Yes:1 No:0(1) | -.587 | .734 | .640 | 1 | .424 | .556 | .132 | 2.342 |
|  | History of Stroke Yes:1 No:0(1) | 2.189 | 1.090 | 4.031 | 1 | .045 | 8.923 | 1.053 | 75.581 |
|  | History of Transient Ischemic Attack Yes:1 No:0(1) | -16.454 | 25408.221 | .000 | 1 | .999 | .000 | .000 | . |
|  | History of Peripheral Vascular Disease Yes:1 No:0(1) | .377 | 1.165 | .105 | 1 | .746 | 1.458 | .149 | 14.316 |
|  | History of Cancer Yes:1 No:0(1) | -.401 | .734 | .298 | 1 | .585 | .670 | .159 | 2.825 |
|  | History of Percutaneous Coronary Intervention Yes:1 No:0(1) | -1.032 | 1.022 | 1.020 | 1 | .312 | .356 | .048 | 2.639 |
|  | History of CABG Yes:1 No:0(1) | 1.383 | 1.127 | 1.505 | 1 | .220 | 3.986 | .438 | 36.300 |
|  | History of CKD Yes:1 No:0(1) | -.719 | .891 | .652 | 1 | .419 | .487 | .085 | 2.790 |
|  | Taking ACEi, ARNi, or ARB Yes:1 No:0(1) | -1.884 | .907 | 4.311 | 1 | .038 | .152 | .026 | .900 |
|  | Taking Diuretics Yes:1 No:0(1) | .462 | .753 | .377 | 1 | .539 | 1.588 | .363 | 6.951 |
|  | Heart failure diagnosis (0=No HF, 1= History of HF, 2=New HF) |  |  | .719 | 2 | .698 |  |  |  |
|  | Heart failure diagnosis (0=No HF, 1= History of HF, 2=New HF)(1) | .238 | .913 | .068 | 1 | .794 | 1.269 | .212 | 7.591 |
|  | Heart failure diagnosis (0=No HF, 1= History of HF, 2=New HF)(2) | .632 | .755 | .701 | 1 | .402 | 1.882 | .428 | 8.268 |
|  | Left ventricular ejection fraction <50% Yes:1 No:0(1) | 1.331 | .772 | 2.973 | 1 | .085 | 3.784 | .834 | 17.181 |
|  | In patient diuretic use(1) | .885 | .759 | 1.359 | 1 | .244 | 2.423 | .547 | 10.723 |
|  | Hyponatremia on admission Yes:1 No:0(1) | 2.137 | .734 | 8.466 | 1 | .004 | 8.471 | 2.008 | 35.725 |
|  | Constant | -4.773 | 1.104 | 18.708 | 1 | <.001 | .008 |  |  |
| Step 4^a^ | SEX Female: 1 Male:0(1) | .563 | .640 | .773 | 1 | .379 | 1.756 | .501 | 6.158 |
|  | Age 1<65 2:≥ 65(1) | .756 | .809 | .871 | 1 | .351 | 2.129 | .436 | 10.402 |
|  | Current Smoker Yes:1 No: 0(1) | -2.401 | 1.207 | 3.956 | 1 | .047 | .091 | .009 | .965 |
|  | History of Diabetes Yes:1 No:0(1) | -.589 | .735 | .641 | 1 | .423 | .555 | .131 | 2.344 |
|  | History of Stroke Yes:1 No:0(1) | 2.185 | 1.093 | 3.994 | 1 | .046 | 8.891 | 1.043 | 75.783 |
|  | History of Peripheral Vascular Disease Yes:1 No:0(1) | .357 | 1.162 | .095 | 1 | .758 | 1.429 | .147 | 13.935 |
|  | History of Cancer Yes:1 No:0(1) | -.400 | .736 | .296 | 1 | .586 | .670 | .158 | 2.834 |
|  | History of Percutaneous Coronary Intervention Yes:1 No:0(1) | -1.052 | 1.016 | 1.072 | 1 | .300 | .349 | .048 | 2.559 |
|  | History of CABG Yes:1 No:0(1) | 1.409 | 1.115 | 1.597 | 1 | .206 | 4.093 | .460 | 36.425 |
|  | History of CKD Yes:1 No:0(1) | -.737 | .885 | .692 | 1 | .405 | .479 | .084 | 2.715 |
|  | Taking ACEi, ARNi, or ARB Yes:1 No:0(1) | -1.890 | .908 | 4.330 | 1 | .037 | .151 | .025 | .896 |
|  | Taking Diuretics Yes:1 No:0(1) | .458 | .754 | .368 | 1 | .544 | 1.580 | .360 | 6.929 |
|  | Heart failure diagnosis (0=No HF, 1= History of HF, 2=New HF) |  |  | .713 | 2 | .700 |  |  |  |
|  | Heart failure diagnosis (0=No HF, 1= History of HF, 2=New HF)(1) | .231 | .913 | .064 | 1 | .801 | 1.259 | .210 | 7.542 |
|  | Heart failure diagnosis (0=No HF, 1= History of HF, 2=New HF)(2) | .629 | .755 | .694 | 1 | .405 | 1.876 | .427 | 8.241 |
|  | Left ventricular ejection fraction <50% Yes:1 No:0(1) | 1.339 | .771 | 3.018 | 1 | .082 | 3.817 | .842 | 17.297 |
|  | In patient diuretic use(1) | .891 | .759 | 1.376 | 1 | .241 | 2.436 | .550 | 10.791 |
|  | Hyponatremia on admission Yes:1 No:0(1) | 2.142 | .735 | 8.499 | 1 | .004 | 8.514 | 2.017 | 35.933 |
|  | Constant | -4.784 | 1.105 | 18.759 | 1 | <.001 | .008 |  |  |
| Step 5^a^ | SEX Female: 1 Male:0(1) | .545 | .638 | .730 | 1 | .393 | 1.724 | .494 | 6.015 |
|  | Age 1<65 2:≥ 65(1) | .707 | .793 | .796 | 1 | .372 | 2.028 | .429 | 9.589 |
|  | Current Smoker Yes:1 No: 0(1) | -2.403 | 1.208 | 3.959 | 1 | .047 | .090 | .008 | .965 |
|  | History of Diabetes Yes:1 No:0(1) | -.513 | .688 | .554 | 1 | .457 | .599 | .155 | 2.309 |
|  | History of Stroke Yes:1 No:0(1) | 2.201 | 1.095 | 4.045 | 1 | .044 | 9.038 | 1.058 | 77.233 |
|  | History of Cancer Yes:1 No:0(1) | -.352 | .716 | .241 | 1 | .623 | .703 | .173 | 2.864 |
|  | History of Percutaneous Coronary Intervention Yes:1 No:0(1) | -1.065 | 1.015 | 1.101 | 1 | .294 | .345 | .047 | 2.520 |
|  | History of CABG Yes:1 No:0(1) | 1.517 | 1.054 | 2.069 | 1 | .150 | 4.557 | .577 | 35.982 |
|  | History of CKD Yes:1 No:0(1) | -.774 | .881 | .772 | 1 | .380 | .461 | .082 | 2.594 |
|  | Taking ACEi, ARNi, or ARB Yes:1 No:0(1) | -1.945 | .897 | 4.703 | 1 | .030 | .143 | .025 | .829 |
|  | Taking Diuretics Yes:1 No:0(1) | .505 | .741 | .466 | 1 | .495 | 1.658 | .388 | 7.081 |
|  | Heart failure diagnosis (0=No HF, 1= History of HF, 2=New HF) |  |  | .677 | 2 | .713 |  |  |  |
|  | Heart failure diagnosis (0=No HF, 1= History of HF, 2=New HF)(1) | .234 | .918 | .065 | 1 | .799 | 1.263 | .209 | 7.632 |
|  | Heart failure diagnosis (0=No HF, 1= History of HF, 2=New HF)(2) | .614 | .754 | .661 | 1 | .416 | 1.847 | .421 | 8.103 |
|  | Left ventricular ejection fraction <50% Yes:1 No:0(1) | 1.349 | .771 | 3.062 | 1 | .080 | 3.853 | .851 | 17.454 |
|  | In patient diuretic use(1) | .900 | .759 | 1.405 | 1 | .236 | 2.460 | .555 | 10.897 |
|  | Hyponatremia on admission Yes:1 No:0(1) | 2.156 | .735 | 8.610 | 1 | .003 | 8.634 | 2.046 | 36.440 |
|  | Constant | -4.782 | 1.109 | 18.599 | 1 | <.001 | .008 |  |  |
| Step 6^a^ | SEX Female: 1 Male:0(1) | .587 | .631 | .867 | 1 | .352 | 1.799 | .523 | 6.192 |
|  | Age 1<65 2:≥ 65(1) | .785 | .776 | 1.025 | 1 | .311 | 2.193 | .479 | 10.034 |
|  | Current Smoker Yes:1 No: 0(1) | -2.358 | 1.178 | 4.009 | 1 | .045 | .095 | .009 | .951 |
|  | History of Diabetes Yes:1 No:0(1) | -.508 | .687 | .548 | 1 | .459 | .602 | .157 | 2.311 |
|  | History of Stroke Yes:1 No:0(1) | 2.197 | 1.093 | 4.042 | 1 | .044 | 9.001 | 1.057 | 76.669 |
|  | History of Cancer Yes:1 No:0(1) | -.416 | .711 | .343 | 1 | .558 | .660 | .164 | 2.656 |
|  | History of Percutaneous Coronary Intervention Yes:1 No:0(1) | -.992 | 1.010 | .965 | 1 | .326 | .371 | .051 | 2.684 |
|  | History of CABG Yes:1 No:0(1) | 1.481 | 1.040 | 2.030 | 1 | .154 | 4.399 | .573 | 33.758 |
|  | History of CKD Yes:1 No:0(1) | -.830 | .860 | .932 | 1 | .334 | .436 | .081 | 2.352 |
|  | Taking ACEi, ARNi, or ARB Yes:1 No:0(1) | -1.999 | .896 | 4.972 | 1 | .026 | .135 | .023 | .785 |
|  | Taking Diuretics Yes:1 No:0(1) | .523 | .705 | .550 | 1 | .458 | 1.687 | .424 | 6.715 |
|  | Left ventricular ejection fraction <50% Yes:1 No:0(1) | 1.515 | .750 | 4.083 | 1 | .043 | 4.549 | 1.047 | 19.768 |
|  | In patient diuretic use(1) | .925 | .750 | 1.520 | 1 | .218 | 2.521 | .580 | 10.964 |
|  | Hyponatremia on admission Yes:1 No:0(1) | 2.193 | .728 | 9.064 | 1 | .003 | 8.959 | 2.149 | 37.339 |
|  | Constant | -4.706 | 1.094 | 18.514 | 1 | <.001 | .009 |  |  |
| Step 7^a^ | SEX Female: 1 Male:0(1) | .551 | .626 | .775 | 1 | .379 | 1.735 | .509 | 5.911 |
|  | Age 1<65 2:≥ 65(1) | .706 | .755 | .876 | 1 | .349 | 2.027 | .462 | 8.898 |
|  | Current Smoker Yes:1 No: 0(1) | -2.245 | 1.156 | 3.773 | 1 | .052 | .106 | .011 | 1.021 |
|  | History of Diabetes Yes:1 No:0(1) | -.418 | .667 | .393 | 1 | .531 | .658 | .178 | 2.434 |
|  | History of Stroke Yes:1 No:0(1) | 2.172 | 1.086 | 4.001 | 1 | .045 | 8.775 | 1.045 | 73.698 |
|  | History of Percutaneous Coronary Intervention Yes:1 No:0(1) | -.978 | 1.001 | .955 | 1 | .328 | .376 | .053 | 2.673 |
|  | History of CABG Yes:1 No:0(1) | 1.447 | 1.038 | 1.945 | 1 | .163 | 4.252 | .556 | 32.515 |
|  | History of CKD Yes:1 No:0(1) | -.777 | .851 | .834 | 1 | .361 | .460 | .087 | 2.437 |
|  | Taking ACEi, ARNi, or ARB Yes:1 No:0(1) | -1.917 | .882 | 4.726 | 1 | .030 | .147 | .026 | .828 |
|  | Taking Diuretics Yes:1 No:0(1) | .568 | .702 | .655 | 1 | .418 | 1.765 | .446 | 6.988 |
|  | Left ventricular ejection fraction <50% Yes:1 No:0(1) | 1.489 | .748 | 3.960 | 1 | .047 | 4.432 | 1.023 | 19.206 |
|  | In patient diuretic use(1) | .919 | .745 | 1.522 | 1 | .217 | 2.506 | .582 | 10.790 |
|  | Hyponatremia on admission Yes:1 No:0(1) | 2.114 | .713 | 8.793 | 1 | .003 | 8.280 | 2.048 | 33.480 |
|  | Constant | -4.800 | 1.084 | 19.607 | 1 | <.001 | .008 |  |  |
| Step 8^a^ | SEX Female: 1 Male:0(1) | .537 | .625 | .740 | 1 | .390 | 1.712 | .503 | 5.825 |
|  | Age 1<65 2:≥ 65(1) | .748 | .753 | .987 | 1 | .320 | 2.113 | .483 | 9.245 |
|  | Current Smoker Yes:1 No: 0(1) | -2.199 | 1.145 | 3.687 | 1 | .055 | .111 | .012 | 1.047 |
|  | History of Stroke Yes:1 No:0(1) | 2.092 | 1.080 | 3.755 | 1 | .053 | 8.103 | .976 | 67.242 |
|  | History of Percutaneous Coronary Intervention Yes:1 No:0(1) | -.944 | .967 | .953 | 1 | .329 | .389 | .058 | 2.590 |
|  | History of CABG Yes:1 No:0(1) | 1.297 | .978 | 1.759 | 1 | .185 | 3.657 | .538 | 24.851 |
|  | History of CKD Yes:1 No:0(1) | -.813 | .838 | .941 | 1 | .332 | .444 | .086 | 2.293 |
|  | Taking ACEi, ARNi, or ARB Yes:1 No:0(1) | -2.005 | .865 | 5.375 | 1 | .020 | .135 | .025 | .733 |
|  | Taking Diuretics Yes:1 No:0(1) | .569 | .700 | .660 | 1 | .417 | 1.766 | .448 | 6.965 |
|  | Left ventricular ejection fraction <50% Yes:1 No:0(1) | 1.450 | .751 | 3.729 | 1 | .053 | 4.262 | .978 | 18.567 |
|  | In patient diuretic use(1) | .902 | .746 | 1.461 | 1 | .227 | 2.465 | .571 | 10.645 |
|  | Hyponatremia on admission Yes:1 No:0(1) | 2.162 | .714 | 9.176 | 1 | .002 | 8.692 | 2.145 | 35.215 |
|  | Constant | -4.927 | 1.080 | 20.791 | 1 | <.001 | .007 |  |  |
| Step 9^a^ | SEX Female: 1 Male:0(1) | .586 | .624 | .881 | 1 | .348 | 1.796 | .529 | 6.105 |
|  | Age 1<65 2:≥ 65(1) | .823 | .750 | 1.205 | 1 | .272 | 2.278 | .524 | 9.903 |
|  | Current Smoker Yes:1 No: 0(1) | -2.224 | 1.143 | 3.789 | 1 | .052 | .108 | .012 | 1.015 |
|  | History of Stroke Yes:1 No:0(1) | 1.964 | 1.057 | 3.457 | 1 | .063 | 7.131 | .899 | 56.552 |
|  | History of Percutaneous Coronary Intervention Yes:1 No:0(1) | -.955 | .960 | .989 | 1 | .320 | .385 | .059 | 2.527 |
|  | History of CABG Yes:1 No:0(1) | 1.301 | .981 | 1.758 | 1 | .185 | 3.674 | .537 | 25.149 |
|  | History of CKD Yes:1 No:0(1) | -.762 | .833 | .836 | 1 | .360 | .467 | .091 | 2.389 |
|  | Taking ACEi, ARNi, or ARB Yes:1 No:0(1) | -1.864 | .831 | 5.030 | 1 | .025 | .155 | .030 | .791 |
|  | Left ventricular ejection fraction <50% Yes:1 No:0(1) | 1.456 | .758 | 3.692 | 1 | .055 | 4.288 | .971 | 18.929 |
|  | In patient diuretic use(1) | .905 | .746 | 1.473 | 1 | .225 | 2.472 | .573 | 10.663 |
|  | Hyponatremia on admission Yes:1 No:0(1) | 2.024 | .681 | 8.835 | 1 | .003 | 7.569 | 1.993 | 28.753 |
|  | Constant | -4.786 | 1.052 | 20.683 | 1 | <.001 | .008 |  |  |
| Step 10^a^ | Age 1<65 2:≥ 65(1) | .888 | .753 | 1.390 | 1 | .238 | 2.429 | .555 | 10.624 |
|  | Current Smoker Yes:1 No: 0(1) | -2.272 | 1.148 | 3.916 | 1 | .048 | .103 | .011 | .979 |
|  | History of Stroke Yes:1 No:0(1) | 2.083 | 1.058 | 3.876 | 1 | .049 | 8.027 | 1.009 | 63.835 |
|  | History of Percutaneous Coronary Intervention Yes:1 No:0(1) | -1.033 | .937 | 1.216 | 1 | .270 | .356 | .057 | 2.233 |
|  | History of CABG Yes:1 No:0(1) | 1.248 | .963 | 1.679 | 1 | .195 | 3.484 | .527 | 23.017 |
|  | History of CKD Yes:1 No:0(1) | -.764 | .829 | .849 | 1 | .357 | .466 | .092 | 2.365 |
|  | Taking ACEi, ARNi, or ARB Yes:1 No:0(1) | -1.837 | .832 | 4.879 | 1 | .027 | .159 | .031 | .813 |
|  | Left ventricular ejection fraction <50% Yes:1 No:0(1) | 1.435 | .759 | 3.576 | 1 | .059 | 4.200 | .949 | 18.585 |
|  | In patient diuretic use(1) | .879 | .735 | 1.430 | 1 | .232 | 2.408 | .570 | 10.166 |
|  | Hyponatremia on admission Yes:1 No:0(1) | 2.044 | .677 | 9.109 | 1 | .003 | 7.720 | 2.047 | 29.108 |
|  | Constant | -4.521 | .995 | 20.650 | 1 | <.001 | .011 |  |  |
| Step 11^a^ | Age 1<65 2:≥ 65(1) | .666 | .705 | .892 | 1 | .345 | 1.947 | .489 | 7.756 |
|  | Current Smoker Yes:1 No: 0(1) | -2.161 | 1.148 | 3.540 | 1 | .060 | .115 | .012 | 1.094 |
|  | History of Stroke Yes:1 No:0(1) | 1.926 | 1.051 | 3.355 | 1 | .067 | 6.859 | .874 | 53.838 |
|  | History of Percutaneous Coronary Intervention Yes:1 No:0(1) | -.775 | .869 | .796 | 1 | .372 | .461 | .084 | 2.528 |
|  | History of CABG Yes:1 No:0(1) | .861 | .848 | 1.031 | 1 | .310 | 2.365 | .449 | 12.464 |
|  | Taking ACEi, ARNi, or ARB Yes:1 No:0(1) | -1.608 | .779 | 4.266 | 1 | .039 | .200 | .044 | .921 |
|  | Left ventricular ejection fraction <50% Yes:1 No:0(1) | 1.390 | .754 | 3.398 | 1 | .065 | 4.015 | .916 | 17.601 |
|  | In patient diuretic use(1) | .881 | .730 | 1.455 | 1 | .228 | 2.413 | .577 | 10.091 |
|  | Hyponatremia on admission Yes:1 No:0(1) | 1.977 | .670 | 8.722 | 1 | .003 | 7.224 | 1.945 | 26.837 |
|  | Constant | -4.536 | .973 | 21.735 | 1 | <.001 | .011 |  |  |
| Step 12^a^ | Age 1<65 2:≥ 65(1) | .771 | .695 | 1.230 | 1 | .267 | 2.163 | .553 | 8.451 |
|  | Current Smoker Yes:1 No: 0(1) | -2.039 | 1.124 | 3.292 | 1 | .070 | .130 | .014 | 1.178 |
|  | History of Stroke Yes:1 No:0(1) | 1.647 | .988 | 2.782 | 1 | .095 | 5.193 | .749 | 35.986 |
|  | History of CABG Yes:1 No:0(1) | .462 | .727 | .403 | 1 | .525 | 1.587 | .382 | 6.601 |
|  | Taking ACEi, ARNi, or ARB Yes:1 No:0(1) | -1.531 | .763 | 4.022 | 1 | .045 | .216 | .048 | .966 |
|  | Left ventricular ejection fraction <50% Yes:1 No:0(1) | 1.219 | .717 | 2.889 | 1 | .089 | 3.384 | .830 | 13.799 |
|  | In patient diuretic use(1) | .850 | .727 | 1.366 | 1 | .243 | 2.340 | .562 | 9.735 |
|  | Hyponatremia on admission Yes:1 No:0(1) | 2.086 | .667 | 9.783 | 1 | .002 | 8.051 | 2.179 | 29.752 |
|  | Constant | -4.630 | .974 | 22.596 | 1 | <.001 | .010 |  |  |
| Step 13^a^ | Age 1<65 2:≥ 65(1) | .767 | .689 | 1.240 | 1 | .265 | 2.154 | .558 | 8.309 |
|  | Current Smoker Yes:1 No: 0(1) | -2.123 | 1.120 | 3.593 | 1 | .058 | .120 | .013 | 1.075 |
|  | History of Stroke Yes:1 No:0(1) | 1.681 | .988 | 2.891 | 1 | .089 | 5.368 | .774 | 37.252 |
|  | Taking ACEi, ARNi, or ARB Yes:1 No:0(1) | -1.518 | .767 | 3.919 | 1 | .048 | .219 | .049 | .985 |
|  | Left ventricular ejection fraction <50% Yes:1 No:0(1) | 1.260 | .708 | 3.169 | 1 | .075 | 3.525 | .881 | 14.109 |
|  | In patient diuretic use(1) | .936 | .721 | 1.689 | 1 | .194 | 2.551 | .621 | 10.473 |
|  | Hyponatremia on admission Yes:1 No:0(1) | 2.094 | .665 | 9.920 | 1 | .002 | 8.118 | 2.205 | 29.882 |
|  | Constant | -4.626 | .976 | 22.462 | 1 | <.001 | .010 |  |  |
| Step 14^a^ | Current Smoker Yes:1 No: 0(1) | -2.384 | 1.108 | 4.630 | 1 | .031 | .092 | .011 | .809 |
|  | History of Stroke Yes:1 No:0(1) | 1.850 | .964 | 3.686 | 1 | .055 | 6.360 | .962 | 42.032 |
|  | Taking ACEi, ARNi, or ARB Yes:1 No:0(1) | -1.371 | .744 | 3.392 | 1 | .066 | .254 | .059 | 1.092 |
|  | Left ventricular ejection fraction <50% Yes:1 No:0(1) | 1.283 | .704 | 3.321 | 1 | .068 | 3.606 | .908 | 14.323 |
|  | In patient diuretic use(1) | .985 | .709 | 1.930 | 1 | .165 | 2.679 | .667 | 10.759 |
|  | Hyponatremia on admission Yes:1 No:0(1) | 1.914 | .632 | 9.180 | 1 | .002 | 6.781 | 1.966 | 23.391 |
|  | Constant | -4.158 | .823 | 25.533 | 1 | <.001 | .016 |  |  |
| Step 15^a^ | Current Smoker Yes:1 No: 0(1) | -2.295 | 1.090 | 4.437 | 1 | .035 | .101 | .012 | .853 |
|  | History of Stroke Yes:1 No:0(1) | 1.628 | .940 | 2.999 | 1 | .083 | 5.094 | .807 | 32.160 |
|  | Taking ACEi, ARNi, or ARB Yes:1 No:0(1) | -1.165 | .709 | 2.701 | 1 | .100 | .312 | .078 | 1.252 |
|  | Left ventricular ejection fraction <50% Yes:1 No:0(1) | 1.524 | .680 | 5.017 | 1 | .025 | 4.592 | 1.210 | 17.428 |
|  | Hyponatremia on admission Yes:1 No:0(1) | 1.726 | .601 | 8.259 | 1 | .004 | 5.618 | 1.731 | 18.231 |
|  | Constant | -3.612 | .657 | 30.200 | 1 | <.001 | .027 |  |  |
| Step 16^a^ | Current Smoker Yes:1 No: 0(1) | -2.289 | 1.089 | 4.418 | 1 | .036 | .101 | .012 | .857 |
|  | Taking ACEi, ARNi, or ARB Yes:1 No:0(1) | -1.071 | .701 | 2.335 | 1 | .126 | .343 | .087 | 1.353 |
|  | Left ventricular ejection fraction <50% Yes:1 No:0(1) | 1.263 | .631 | 4.004 | 1 | .045 | 3.538 | 1.026 | 12.196 |
|  | Hyponatremia on admission Yes:1 No:0(1) | 1.661 | .594 | 7.804 | 1 | .005 | 5.262 | 1.641 | 16.870 |
|  | Constant | -3.283 | .570 | 33.176 | 1 | <.001 | .038 |  |  |
| Step 17^a^ | Current Smoker Yes:1 No: 0(1) | -2.098 | 1.074 | 3.815 | 1 | .051 | .123 | .015 | 1.007 |
|  | Left ventricular ejection fraction <50% Yes:1 No:0(1) | 1.270 | .625 | 4.130 | 1 | .042 | 3.559 | 1.046 | 12.111 |
|  | Hyponatremia on admission Yes:1 No:0(1) | 1.577 | .579 | 7.415 | 1 | .006 | 4.840 | 1.556 | 15.059 |
|  | Constant | -3.579 | .557 | 41.317 | 1 | <.001 | .028 |  |  |
| a. Variable(s) entered on step 1: SEX Female: 1 Male:0, Age 1<65 2:≥ 65, Current Smoker Yes:1 No: 0, History of HTN Yes:1 No:0, History of Diabetes Yes:1 No:0, History of Stroke Yes:1 No:0, History of Transient Ischemic Attack Yes:1 No:0, History of Peripheral Vascular Disease Yes:1 No:0, History of Cancer Yes:1 No:0, History of Myocardial Infarction Yes:1 No:0, History of Percutaneous Coronary Intervention Yes:1 No:0, History of CABG Yes:1 No:0, History of CKD Yes:1 No:0, Taking ACEi, ARNi, or ARB Yes:1 No:0, Taking Diuretics Yes:1 No:0, Heart failure diagnosis (0=No HF, 1= History of HF, 2=New HF), Left ventricular ejection fraction <50% Yes:1 No:0, In patient diuretic use, Hyponatremia on admission Yes:1 No:0. | | | | | | | | | |

**Dependent variable: 30-day mortality**

**Independent variable: Hyponatremia on admission**

| **Omnibus Tests of Model Coefficients** | | | | |
| --- | --- | --- | --- | --- |
|  | | Chi-square | df | Sig. |
| Step 1 | Step | 37.350 | 20 | .011 |
|  | Block | 37.350 | 20 | .011 |
|  | Model | 37.350 | 20 | .011 |
| Step 2^a^ | Step | .000 | 1 | .987 |
|  | Block | 37.349 | 19 | .007 |
|  | Model | 37.349 | 19 | .007 |
| Step 3^a^ | Step | -.042 | 1 | .837 |
|  | Block | 37.307 | 18 | .005 |
|  | Model | 37.307 | 18 | .005 |
| Step 4^a^ | Step | -.058 | 1 | .809 |
|  | Block | 37.249 | 17 | .003 |
|  | Model | 37.249 | 17 | .003 |
| Step 5^a^ | Step | -.061 | 1 | .805 |
|  | Block | 37.188 | 16 | .002 |
|  | Model | 37.188 | 16 | .002 |
| Step 6^a^ | Step | -.089 | 1 | .766 |
|  | Block | 37.100 | 15 | .001 |
|  | Model | 37.100 | 15 | .001 |
| Step 7^a^ | Step | -.854 | 2 | .652 |
|  | Block | 36.246 | 13 | <.001 |
|  | Model | 36.246 | 14 | <.001 |
| Step 8^a^ | Step | -.257 | 1 | .612 |
|  | Block | 35.988 | 12 | <.001 |
|  | Model | 35.988 | 12 | <.001 |
| Step 9^a^ | Step | -.279 | 1 | .598 |
|  | Block | 35.710 | 11 | <.001 |
|  | Model | 35.710 | 11 | <.001 |
| Step 10^a^ | Step | -.456 | 1 | .499 |
|  | Block | 35.253 | 10 | <.001 |
|  | Model | 35.253 | 10 | <.001 |
| Step 11^a^ | Step | -.771 | 1 | .380 |
|  | Block | 34.482 | 9 | <.001 |
|  | Model | 34.482 | 9 | <.001 |
| Step 12^a^ | Step | -1.731 | 1 | .188 |
|  | Block | 32.751 | 8 | <.001 |
|  | Model | 32.751 | 8 | <.001 |
| Step 13^a^ | Step | -2.655 | 1 | .103 |
|  | Block | 30.096 | 7 | <.001 |
|  | Model | 30.096 | 7 | <.001 |
| Step 14^a^ | Step | -2.456 | 1 | .117 |
|  | Block | 27.639 | 6 | <.001 |
|  | Model | 27.639 | 6 | <.001 |
| a. A negative Chi-squares value indicates that the Chi-squares value has decreased from the previous step. | | | | |

| **Hosmer and Lemeshow Test** | | | |
| --- | --- | --- | --- |
| Step | Chi-square | df | Sig. |
| 1 | 5.608 | 8 | .691 |
| 2 | 5.601 | 8 | .692 |
| 3 | 5.610 | 8 | .691 |
| 4 | 4.794 | 8 | .779 |
| 5 | 5.441 | 8 | .710 |
| 6 | 5.455 | 8 | .708 |
| 7 | 6.073 | 8 | .639 |
| 8 | 3.777 | 8 | .877 |
| 9 | 7.813 | 8 | .452 |
| 10 | 9.248 | 8 | .322 |
| 11 | 6.928 | 8 | .544 |
| 12 | 4.303 | 8 | .829 |
| 13 | 4.696 | 8 | .789 |
| 14 | 4.898 | 8 | .768 |

| **Classification Table**^a^ | | | | | |
| --- | --- | --- | --- | --- | --- |
|  | Observed | | Predicted | | |
|  |  |  | 30 day Mortality Yes:1 No:0 | | Percentage Correct |
|  |  |  | no | yes |  |
| Step 1 | 30 day Mortality Yes:1 No:0 | no | 197 | 4 | 98.0 |
|  |  | yes | 16 | 4 | 20.0 |
|  | Overall Percentage | |  |  | 91.0 |
| Step 2 | 30 day Mortality Yes:1 No:0 | no | 197 | 4 | 98.0 |
|  |  | yes | 16 | 4 | 20.0 |
|  | Overall Percentage | |  |  | 91.0 |
| Step 3 | 30 day Mortality Yes:1 No:0 | no | 197 | 4 | 98.0 |
|  |  | yes | 16 | 4 | 20.0 |
|  | Overall Percentage | |  |  | 91.0 |
| Step 4 | 30 day Mortality Yes:1 No:0 | no | 196 | 5 | 97.5 |
|  |  | yes | 16 | 4 | 20.0 |
|  | Overall Percentage | |  |  | 90.5 |
| Step 5 | 30 day Mortality Yes:1 No:0 | no | 196 | 5 | 97.5 |
|  |  | yes | 17 | 3 | 15.0 |
|  | Overall Percentage | |  |  | 90.0 |
| Step 6 | 30 day Mortality Yes:1 No:0 | no | 196 | 5 | 97.5 |
|  |  | yes | 17 | 3 | 15.0 |
|  | Overall Percentage | |  |  | 90.0 |
| Step 7 | 30 day Mortality Yes:1 No:0 | no | 199 | 2 | 99.0 |
|  |  | yes | 16 | 4 | 20.0 |
|  | Overall Percentage | |  |  | 91.9 |
| Step 8 | 30 day Mortality Yes:1 No:0 | no | 196 | 5 | 97.5 |
|  |  | yes | 16 | 4 | 20.0 |
|  | Overall Percentage | |  |  | 90.5 |
| Step 9 | 30 day Mortality Yes:1 No:0 | no | 198 | 3 | 98.5 |
|  |  | yes | 17 | 3 | 15.0 |
|  | Overall Percentage | |  |  | 91.0 |
| Step 10 | 30 day Mortality Yes:1 No:0 | no | 196 | 5 | 97.5 |
|  |  | yes | 16 | 4 | 20.0 |
|  | Overall Percentage | |  |  | 90.5 |
| Step 11 | 30 day Mortality Yes:1 No:0 | no | 197 | 4 | 98.0 |
|  |  | yes | 16 | 4 | 20.0 |
|  | Overall Percentage | |  |  | 91.0 |
| Step 12 | 30 day Mortality Yes:1 No:0 | no | 196 | 5 | 97.5 |
|  |  | yes | 17 | 3 | 15.0 |
|  | Overall Percentage | |  |  | 90.0 |
| Step 13 | 30 day Mortality Yes:1 No:0 | no | 198 | 3 | 98.5 |
|  |  | yes | 19 | 1 | 5.0 |
|  | Overall Percentage | |  |  | 90.0 |
| Step 14 | 30 day Mortality Yes:1 No:0 | no | 197 | 4 | 98.0 |
|  |  | yes | 18 | 2 | 10.0 |
|  | Overall Percentage | |  |  | 90.0 |
| a. The cut value is .500 | | | | | |

| **Variables in the Equation** | | | | | | | | | |
| --- | --- | --- | --- | --- | --- | --- | --- | --- | --- |
|  | | B | S.E. | Wald | df | Sig. | Exp(B) | 95% C.I.for EXP(B) | |
|  |  |  |  |  |  |  |  | Lower | Upper |
| Step 1^a^ | SEX Female: 1 Male:0(1) | .660 | .598 | 1.219 | 1 | .270 | 1.935 | .599 | 6.245 |
|  | Age 1<65 2:≥ 65(1) | .136 | .682 | .040 | 1 | .842 | 1.146 | .301 | 4.364 |
|  | Current Smoker Yes:1 No: 0(1) | -1.815 | .947 | 3.669 | 1 | .055 | .163 | .025 | 1.043 |
|  | History of HTN Yes:1 No:0(1) | -.242 | .875 | .076 | 1 | .782 | .785 | .141 | 4.365 |
|  | History of Diabetes Yes:1 No:0(1) | -1.120 | .695 | 2.594 | 1 | .107 | .326 | .084 | 1.275 |
|  | History of Stroke Yes:1 No:0(1) | 2.404 | .885 | 7.382 | 1 | .007 | 11.067 | 1.954 | 62.683 |
|  | History of Transient Ischemic Attack Yes:1 No:0(1) | -18.466 | 26054.387 | .000 | 1 | .999 | .000 | .000 | . |
|  | History of Peripheral Vascular Disease Yes:1 No:0(1) | -.017 | 1.026 | .000 | 1 | .987 | .983 | .132 | 7.345 |
|  | History of Cancer Yes:1 No:0(1) | -.164 | .633 | .067 | 1 | .796 | .849 | .246 | 2.934 |
|  | History of Myocardial Infarction Yes:1 No:0(1) | .287 | .868 | .109 | 1 | .741 | 1.332 | .243 | 7.298 |
|  | History of Percutaneous Coronary Intervention Yes:1 No:0(1) | -.571 | .936 | .372 | 1 | .542 | .565 | .090 | 3.538 |
|  | History of CABG Yes:1 No:0(1) | 1.566 | .938 | 2.789 | 1 | .095 | 4.786 | .762 | 30.065 |
|  | History of CKD Yes:1 No:0(1) | .746 | .734 | 1.035 | 1 | .309 | 2.109 | .501 | 8.883 |
|  | Taking ACEi, ARNi, or ARB Yes:1 No:0(1) | -1.265 | .747 | 2.871 | 1 | .090 | .282 | .065 | 1.219 |
|  | Taking Diuretics Yes:1 No:0(1) | .648 | .657 | .975 | 1 | .323 | 1.913 | .528 | 6.928 |
|  | Heart failure diagnosis (0=No HF, 1= History of HF, 2=New HF) |  |  | .936 | 2 | .626 |  |  |  |
|  | Heart failure diagnosis (0=No HF, 1= History of HF, 2=New HF)(1) | -.279 | .810 | .119 | 1 | .731 | .756 | .155 | 3.703 |
|  | Heart failure diagnosis (0=No HF, 1= History of HF, 2=New HF)(2) | .508 | .696 | .533 | 1 | .465 | 1.663 | .425 | 6.507 |
|  | Left ventricular ejection fraction <50% Yes:1 No:0(1) | 1.141 | .702 | 2.641 | 1 | .104 | 3.131 | .790 | 12.400 |
|  | In patient diuretic use(1) | .309 | .614 | .253 | 1 | .615 | 1.362 | .409 | 4.540 |
|  | Hyponatremia on admission Yes:1 No:0(1) | 1.772 | .653 | 7.359 | 1 | .007 | 5.885 | 1.635 | 21.176 |
|  | Constant | -3.764 | 1.013 | 13.809 | 1 | <.001 | .023 |  |  |
| Step 2^a^ | SEX Female: 1 Male:0(1) | .661 | .597 | 1.226 | 1 | .268 | 1.936 | .601 | 6.233 |
|  | Age 1<65 2:≥ 65(1) | .138 | .673 | .042 | 1 | .837 | 1.148 | .307 | 4.295 |
|  | Current Smoker Yes:1 No: 0(1) | -1.816 | .946 | 3.682 | 1 | .055 | .163 | .025 | 1.040 |
|  | History of HTN Yes:1 No:0(1) | -.244 | .864 | .080 | 1 | .778 | .783 | .144 | 4.264 |
|  | History of Diabetes Yes:1 No:0(1) | -1.123 | .671 | 2.802 | 1 | .094 | .325 | .087 | 1.211 |
|  | History of Stroke Yes:1 No:0(1) | 2.404 | .885 | 7.384 | 1 | .007 | 11.066 | 1.954 | 62.662 |
|  | History of Transient Ischemic Attack Yes:1 No:0(1) | -18.480 | 26052.579 | .000 | 1 | .999 | .000 | .000 | . |
|  | History of Cancer Yes:1 No:0(1) | -.165 | .626 | .070 | 1 | .792 | .848 | .248 | 2.892 |
|  | History of Myocardial Infarction Yes:1 No:0(1) | .286 | .865 | .109 | 1 | .741 | 1.330 | .244 | 7.247 |
|  | History of Percutaneous Coronary Intervention Yes:1 No:0(1) | -.569 | .932 | .373 | 1 | .541 | .566 | .091 | 3.514 |
|  | History of CABG Yes:1 No:0(1) | 1.562 | .904 | 2.986 | 1 | .084 | 4.766 | .811 | 28.014 |
|  | History of CKD Yes:1 No:0(1) | .748 | .725 | 1.066 | 1 | .302 | 2.113 | .511 | 8.747 |
|  | Taking ACEi, ARNi, or ARB Yes:1 No:0(1) | -1.264 | .738 | 2.931 | 1 | .087 | .283 | .067 | 1.201 |
|  | Taking Diuretics Yes:1 No:0(1) | .647 | .651 | .987 | 1 | .320 | 1.910 | .533 | 6.845 |
|  | Heart failure diagnosis (0=No HF, 1= History of HF, 2=New HF) |  |  | .942 | 2 | .624 |  |  |  |
|  | Heart failure diagnosis (0=No HF, 1= History of HF, 2=New HF)(1) | -.278 | .809 | .118 | 1 | .731 | .757 | .155 | 3.694 |
|  | Heart failure diagnosis (0=No HF, 1= History of HF, 2=New HF)(2) | .509 | .693 | .540 | 1 | .462 | 1.664 | .428 | 6.477 |
|  | Left ventricular ejection fraction <50% Yes:1 No:0(1) | 1.140 | .698 | 2.665 | 1 | .103 | 3.127 | .795 | 12.292 |
|  | In patient diuretic use(1) | .310 | .613 | .256 | 1 | .613 | 1.363 | .410 | 4.531 |
|  | Hyponatremia on admission Yes:1 No:0(1) | 1.772 | .653 | 7.361 | 1 | .007 | 5.883 | 1.635 | 21.164 |
|  | Constant | -3.763 | 1.012 | 13.825 | 1 | <.001 | .023 |  |  |
| Step 3^a^ | SEX Female: 1 Male:0(1) | .672 | .593 | 1.281 | 1 | .258 | 1.957 | .612 | 6.263 |
|  | Current Smoker Yes:1 No: 0(1) | -1.857 | .932 | 3.967 | 1 | .046 | .156 | .025 | .971 |
|  | History of HTN Yes:1 No:0(1) | -.228 | .860 | .070 | 1 | .791 | .796 | .147 | 4.300 |
|  | History of Diabetes Yes:1 No:0(1) | -1.126 | .672 | 2.807 | 1 | .094 | .324 | .087 | 1.211 |
|  | History of Stroke Yes:1 No:0(1) | 2.425 | .879 | 7.611 | 1 | .006 | 11.298 | 2.018 | 63.258 |
|  | History of Transient Ischemic Attack Yes:1 No:0(1) | -18.550 | 26015.863 | .000 | 1 | .999 | .000 | .000 | . |
|  | History of Cancer Yes:1 No:0(1) | -.149 | .620 | .058 | 1 | .810 | .862 | .256 | 2.905 |
|  | History of Myocardial Infarction Yes:1 No:0(1) | .265 | .861 | .095 | 1 | .758 | 1.304 | .241 | 7.047 |
|  | History of Percutaneous Coronary Intervention Yes:1 No:0(1) | -.583 | .929 | .395 | 1 | .530 | .558 | .090 | 3.445 |
|  | History of CABG Yes:1 No:0(1) | 1.584 | .901 | 3.092 | 1 | .079 | 4.875 | .834 | 28.494 |
|  | History of CKD Yes:1 No:0(1) | .787 | .699 | 1.267 | 1 | .260 | 2.196 | .558 | 8.643 |
|  | Taking ACEi, ARNi, or ARB Yes:1 No:0(1) | -1.231 | .719 | 2.929 | 1 | .087 | .292 | .071 | 1.196 |
|  | Taking Diuretics Yes:1 No:0(1) | .660 | .648 | 1.038 | 1 | .308 | 1.935 | .543 | 6.891 |
|  | Heart failure diagnosis (0=No HF, 1= History of HF, 2=New HF) |  |  | .997 | 2 | .608 |  |  |  |
|  | Heart failure diagnosis (0=No HF, 1= History of HF, 2=New HF)(1) | -.287 | .809 | .126 | 1 | .723 | .751 | .154 | 3.666 |
|  | Heart failure diagnosis (0=No HF, 1= History of HF, 2=New HF)(2) | .520 | .690 | .569 | 1 | .451 | 1.682 | .435 | 6.504 |
|  | Left ventricular ejection fraction <50% Yes:1 No:0(1) | 1.153 | .696 | 2.745 | 1 | .098 | 3.167 | .810 | 12.389 |
|  | In patient diuretic use(1) | .318 | .611 | .270 | 1 | .603 | 1.374 | .415 | 4.548 |
|  | Hyponatremia on admission Yes:1 No:0(1) | 1.752 | .645 | 7.394 | 1 | .007 | 5.769 | 1.631 | 20.403 |
|  | Constant | -3.732 | .998 | 13.977 | 1 | <.001 | .024 |  |  |
| Step 4^a^ | SEX Female: 1 Male:0(1) | .661 | .591 | 1.249 | 1 | .264 | 1.936 | .608 | 6.167 |
|  | Current Smoker Yes:1 No: 0(1) | -1.808 | .909 | 3.955 | 1 | .047 | .164 | .028 | .974 |
|  | History of HTN Yes:1 No:0(1) | -.212 | .856 | .061 | 1 | .804 | .809 | .151 | 4.329 |
|  | History of Diabetes Yes:1 No:0(1) | -1.097 | .661 | 2.756 | 1 | .097 | .334 | .091 | 1.219 |
|  | History of Stroke Yes:1 No:0(1) | 2.404 | .874 | 7.577 | 1 | .006 | 11.072 | 1.998 | 61.341 |
|  | History of Transient Ischemic Attack Yes:1 No:0(1) | -18.504 | 26064.768 | .000 | 1 | .999 | .000 | .000 | . |
|  | History of Myocardial Infarction Yes:1 No:0(1) | .241 | .852 | .080 | 1 | .777 | 1.272 | .240 | 6.760 |
|  | History of Percutaneous Coronary Intervention Yes:1 No:0(1) | -.584 | .926 | .398 | 1 | .528 | .558 | .091 | 3.424 |
|  | History of CABG Yes:1 No:0(1) | 1.578 | .898 | 3.087 | 1 | .079 | 4.846 | .833 | 28.185 |
|  | History of CKD Yes:1 No:0(1) | .788 | .699 | 1.272 | 1 | .259 | 2.200 | .559 | 8.655 |
|  | Taking ACEi, ARNi, or ARB Yes:1 No:0(1) | -1.222 | .719 | 2.889 | 1 | .089 | .295 | .072 | 1.206 |
|  | Taking Diuretics Yes:1 No:0(1) | .658 | .648 | 1.030 | 1 | .310 | 1.930 | .542 | 6.875 |
|  | Heart failure diagnosis (0=No HF, 1= History of HF, 2=New HF) |  |  | .993 | 2 | .609 |  |  |  |
|  | Heart failure diagnosis (0=No HF, 1= History of HF, 2=New HF)(1) | -.278 | .808 | .119 | 1 | .730 | .757 | .155 | 3.688 |
|  | Heart failure diagnosis (0=No HF, 1= History of HF, 2=New HF)(2) | .525 | .691 | .576 | 1 | .448 | 1.690 | .436 | 6.550 |
|  | Left ventricular ejection fraction <50% Yes:1 No:0(1) | 1.152 | .696 | 2.739 | 1 | .098 | 3.165 | .809 | 12.387 |
|  | In patient diuretic use(1) | .321 | .611 | .276 | 1 | .599 | 1.378 | .416 | 4.562 |
|  | Hyponatremia on admission Yes:1 No:0(1) | 1.731 | .638 | 7.355 | 1 | .007 | 5.647 | 1.616 | 19.733 |
|  | Constant | -3.789 | .973 | 15.168 | 1 | <.001 | .023 |  |  |
| Step 5^a^ | SEX Female: 1 Male:0(1) | .694 | .576 | 1.451 | 1 | .228 | 2.001 | .647 | 6.188 |
|  | Current Smoker Yes:1 No: 0(1) | -1.764 | .888 | 3.950 | 1 | .047 | .171 | .030 | .976 |
|  | History of Diabetes Yes:1 No:0(1) | -1.110 | .660 | 2.833 | 1 | .092 | .329 | .090 | 1.200 |
|  | History of Stroke Yes:1 No:0(1) | 2.432 | .868 | 7.842 | 1 | .005 | 11.383 | 2.075 | 62.446 |
|  | History of Transient Ischemic Attack Yes:1 No:0(1) | -18.478 | 26072.248 | .000 | 1 | .999 | .000 | .000 | . |
|  | History of Myocardial Infarction Yes:1 No:0(1) | .256 | .856 | .089 | 1 | .765 | 1.292 | .241 | 6.907 |
|  | History of Percutaneous Coronary Intervention Yes:1 No:0(1) | -.641 | .901 | .507 | 1 | .477 | .527 | .090 | 3.077 |
|  | History of CABG Yes:1 No:0(1) | 1.573 | .899 | 3.062 | 1 | .080 | 4.820 | .828 | 28.065 |
|  | History of CKD Yes:1 No:0(1) | .733 | .663 | 1.224 | 1 | .269 | 2.081 | .568 | 7.627 |
|  | Taking ACEi, ARNi, or ARB Yes:1 No:0(1) | -1.252 | .708 | 3.127 | 1 | .077 | .286 | .071 | 1.145 |
|  | Taking Diuretics Yes:1 No:0(1) | .616 | .623 | .977 | 1 | .323 | 1.851 | .546 | 6.278 |
|  | Heart failure diagnosis (0=No HF, 1= History of HF, 2=New HF) |  |  | .935 | 2 | .626 |  |  |  |
|  | Heart failure diagnosis (0=No HF, 1= History of HF, 2=New HF)(1) | -.250 | .801 | .098 | 1 | .755 | .778 | .162 | 3.742 |
|  | Heart failure diagnosis (0=No HF, 1= History of HF, 2=New HF)(2) | .497 | .681 | .533 | 1 | .465 | 1.644 | .433 | 6.243 |
|  | Left ventricular ejection fraction <50% Yes:1 No:0(1) | 1.202 | .668 | 3.243 | 1 | .072 | 3.327 | .899 | 12.310 |
|  | In patient diuretic use(1) | .301 | .604 | .248 | 1 | .619 | 1.351 | .413 | 4.414 |
|  | Hyponatremia on admission Yes:1 No:0(1) | 1.710 | .630 | 7.370 | 1 | .007 | 5.530 | 1.609 | 19.008 |
|  | Constant | -3.932 | .796 | 24.417 | 1 | <.001 | .020 |  |  |
| Step 6^a^ | SEX Female: 1 Male:0(1) | .694 | .576 | 1.451 | 1 | .228 | 2.001 | .647 | 6.184 |
|  | Current Smoker Yes:1 No: 0(1) | -1.748 | .886 | 3.891 | 1 | .049 | .174 | .031 | .989 |
|  | History of Diabetes Yes:1 No:0(1) | -1.077 | .646 | 2.776 | 1 | .096 | .341 | .096 | 1.209 |
|  | History of Stroke Yes:1 No:0(1) | 2.380 | .847 | 7.885 | 1 | .005 | 10.800 | 2.052 | 56.851 |
|  | History of Transient Ischemic Attack Yes:1 No:0(1) | -18.347 | 26134.893 | .000 | 1 | .999 | .000 | .000 | . |
|  | History of Percutaneous Coronary Intervention Yes:1 No:0(1) | -.536 | .827 | .420 | 1 | .517 | .585 | .116 | 2.960 |
|  | History of CABG Yes:1 No:0(1) | 1.617 | .892 | 3.285 | 1 | .070 | 5.037 | .877 | 28.937 |
|  | History of CKD Yes:1 No:0(1) | .755 | .658 | 1.316 | 1 | .251 | 2.128 | .586 | 7.736 |
|  | Taking ACEi, ARNi, or ARB Yes:1 No:0(1) | -1.258 | .712 | 3.118 | 1 | .077 | .284 | .070 | 1.148 |
|  | Taking Diuretics Yes:1 No:0(1) | .613 | .625 | .962 | 1 | .327 | 1.847 | .542 | 6.291 |
|  | Heart failure diagnosis (0=No HF, 1= History of HF, 2=New HF) |  |  | .865 | 2 | .649 |  |  |  |
|  | Heart failure diagnosis (0=No HF, 1= History of HF, 2=New HF)(1) | -.249 | .803 | .096 | 1 | .757 | .780 | .161 | 3.763 |
|  | Heart failure diagnosis (0=No HF, 1= History of HF, 2=New HF)(2) | .464 | .671 | .478 | 1 | .489 | 1.590 | .427 | 5.918 |
|  | Left ventricular ejection fraction <50% Yes:1 No:0(1) | 1.191 | .663 | 3.227 | 1 | .072 | 3.292 | .897 | 12.076 |
|  | In patient diuretic use(1) | .313 | .602 | .269 | 1 | .604 | 1.367 | .420 | 4.451 |
|  | Hyponatremia on admission Yes:1 No:0(1) | 1.722 | .630 | 7.478 | 1 | .006 | 5.594 | 1.629 | 19.214 |
|  | Constant | -3.923 | .794 | 24.397 | 1 | <.001 | .020 |  |  |
| Step 7^a^ | SEX Female: 1 Male:0(1) | .760 | .573 | 1.764 | 1 | .184 | 2.139 | .696 | 6.572 |
|  | Current Smoker Yes:1 No: 0(1) | -1.713 | .873 | 3.856 | 1 | .050 | .180 | .033 | .997 |
|  | History of Diabetes Yes:1 No:0(1) | -1.101 | .647 | 2.894 | 1 | .089 | .333 | .094 | 1.182 |
|  | History of Stroke Yes:1 No:0(1) | 2.359 | .840 | 7.892 | 1 | .005 | 10.580 | 2.041 | 54.852 |
|  | History of Transient Ischemic Attack Yes:1 No:0(1) | -18.557 | 25958.268 | .000 | 1 | .999 | .000 | .000 | . |
|  | History of Percutaneous Coronary Intervention Yes:1 No:0(1) | -.439 | .805 | .298 | 1 | .585 | .645 | .133 | 3.120 |
|  | History of CABG Yes:1 No:0(1) | 1.483 | .855 | 3.004 | 1 | .083 | 4.405 | .824 | 23.556 |
|  | History of CKD Yes:1 No:0(1) | .681 | .649 | 1.101 | 1 | .294 | 1.976 | .554 | 7.046 |
|  | Taking ACEi, ARNi, or ARB Yes:1 No:0(1) | -1.313 | .712 | 3.398 | 1 | .065 | .269 | .067 | 1.087 |
|  | Taking Diuretics Yes:1 No:0(1) | .537 | .604 | .789 | 1 | .374 | 1.710 | .523 | 5.588 |
|  | Left ventricular ejection fraction <50% Yes:1 No:0(1) | 1.326 | .638 | 4.316 | 1 | .038 | 3.767 | 1.078 | 13.164 |
|  | In patient diuretic use(1) | .304 | .603 | .254 | 1 | .614 | 1.355 | .416 | 4.414 |
|  | Hyponatremia on admission Yes:1 No:0(1) | 1.652 | .618 | 7.143 | 1 | .008 | 5.216 | 1.553 | 17.516 |
|  | Constant | -3.843 | .775 | 24.590 | 1 | <.001 | .021 |  |  |
| Step 8^a^ | SEX Female: 1 Male:0(1) | .769 | .572 | 1.811 | 1 | .178 | 2.158 | .704 | 6.616 |
|  | Current Smoker Yes:1 No: 0(1) | -1.665 | .860 | 3.750 | 1 | .053 | .189 | .035 | 1.020 |
|  | History of Diabetes Yes:1 No:0(1) | -1.102 | .651 | 2.865 | 1 | .091 | .332 | .093 | 1.190 |
|  | History of Stroke Yes:1 No:0(1) | 2.346 | .836 | 7.869 | 1 | .005 | 10.447 | 2.028 | 53.823 |
|  | History of Transient Ischemic Attack Yes:1 No:0(1) | -18.723 | 25984.660 | .000 | 1 | .999 | .000 | .000 | . |
|  | History of Percutaneous Coronary Intervention Yes:1 No:0(1) | -.413 | .793 | .271 | 1 | .603 | .662 | .140 | 3.134 |
|  | History of CABG Yes:1 No:0(1) | 1.509 | .848 | 3.162 | 1 | .075 | 4.520 | .857 | 23.843 |
|  | History of CKD Yes:1 No:0(1) | .698 | .646 | 1.168 | 1 | .280 | 2.009 | .567 | 7.123 |
|  | Taking ACEi, ARNi, or ARB Yes:1 No:0(1) | -1.245 | .692 | 3.235 | 1 | .072 | .288 | .074 | 1.118 |
|  | Taking Diuretics Yes:1 No:0(1) | .558 | .605 | .850 | 1 | .357 | 1.746 | .534 | 5.713 |
|  | Left ventricular ejection fraction <50% Yes:1 No:0(1) | 1.396 | .625 | 4.986 | 1 | .026 | 4.037 | 1.186 | 13.743 |
|  | Hyponatremia on admission Yes:1 No:0(1) | 1.613 | .612 | 6.938 | 1 | .008 | 5.017 | 1.511 | 16.657 |
|  | Constant | -3.733 | .734 | 25.867 | 1 | <.001 | .024 |  |  |
| Step 9^a^ | SEX Female: 1 Male:0(1) | .810 | .570 | 2.023 | 1 | .155 | 2.249 | .736 | 6.868 |
|  | Current Smoker Yes:1 No: 0(1) | -1.626 | .849 | 3.666 | 1 | .056 | .197 | .037 | 1.039 |
|  | History of Diabetes Yes:1 No:0(1) | -1.084 | .644 | 2.833 | 1 | .092 | .338 | .096 | 1.195 |
|  | History of Stroke Yes:1 No:0(1) | 2.216 | .796 | 7.753 | 1 | .005 | 9.170 | 1.927 | 43.629 |
|  | History of Transient Ischemic Attack Yes:1 No:0(1) | -19.070 | 26091.436 | .000 | 1 | .999 | .000 | .000 | . |
|  | History of CABG Yes:1 No:0(1) | 1.273 | .703 | 3.279 | 1 | .070 | 3.570 | .900 | 14.156 |
|  | History of CKD Yes:1 No:0(1) | .765 | .629 | 1.478 | 1 | .224 | 2.148 | .626 | 7.373 |
|  | Taking ACEi, ARNi, or ARB Yes:1 No:0(1) | -1.266 | .690 | 3.366 | 1 | .067 | .282 | .073 | 1.090 |
|  | Taking Diuretics Yes:1 No:0(1) | .573 | .603 | .904 | 1 | .342 | 1.774 | .544 | 5.788 |
|  | Left ventricular ejection fraction <50% Yes:1 No:0(1) | 1.314 | .602 | 4.757 | 1 | .029 | 3.721 | 1.143 | 12.122 |
|  | Hyponatremia on admission Yes:1 No:0(1) | 1.655 | .608 | 7.408 | 1 | .006 | 5.231 | 1.589 | 17.222 |
|  | Constant | -3.786 | .729 | 26.980 | 1 | <.001 | .023 |  |  |
| Step 10^a^ | SEX Female: 1 Male:0(1) | .859 | .564 | 2.318 | 1 | .128 | 2.360 | .781 | 7.130 |
|  | Current Smoker Yes:1 No: 0(1) | -1.620 | .846 | 3.667 | 1 | .055 | .198 | .038 | 1.039 |
|  | History of Diabetes Yes:1 No:0(1) | -1.133 | .642 | 3.108 | 1 | .078 | .322 | .091 | 1.135 |
|  | History of Stroke Yes:1 No:0(1) | 2.143 | .784 | 7.474 | 1 | .006 | 8.521 | 1.834 | 39.593 |
|  | History of CABG Yes:1 No:0(1) | 1.331 | .697 | 3.649 | 1 | .056 | 3.785 | .966 | 14.835 |
|  | History of CKD Yes:1 No:0(1) | .741 | .631 | 1.380 | 1 | .240 | 2.098 | .609 | 7.223 |
|  | Taking ACEi, ARNi, or ARB Yes:1 No:0(1) | -1.237 | .688 | 3.234 | 1 | .072 | .290 | .075 | 1.117 |
|  | Taking Diuretics Yes:1 No:0(1) | .528 | .599 | .779 | 1 | .378 | 1.696 | .525 | 5.485 |
|  | Left ventricular ejection fraction <50% Yes:1 No:0(1) | 1.331 | .604 | 4.858 | 1 | .028 | 3.786 | 1.159 | 12.367 |
|  | Hyponatremia on admission Yes:1 No:0(1) | 1.652 | .607 | 7.397 | 1 | .007 | 5.215 | 1.586 | 17.148 |
|  | Constant | -3.802 | .731 | 27.065 | 1 | <.001 | .022 |  |  |
| Step 11^a^ | SEX Female: 1 Male:0(1) | .896 | .565 | 2.517 | 1 | .113 | 2.449 | .810 | 7.406 |
|  | Current Smoker Yes:1 No: 0(1) | -1.646 | .845 | 3.789 | 1 | .052 | .193 | .037 | 1.011 |
|  | History of Diabetes Yes:1 No:0(1) | -1.139 | .644 | 3.131 | 1 | .077 | .320 | .091 | 1.130 |
|  | History of Stroke Yes:1 No:0(1) | 2.119 | .780 | 7.379 | 1 | .007 | 8.323 | 1.804 | 38.392 |
|  | History of CABG Yes:1 No:0(1) | 1.357 | .698 | 3.780 | 1 | .052 | 3.886 | .989 | 15.268 |
|  | History of CKD Yes:1 No:0(1) | .822 | .617 | 1.775 | 1 | .183 | 2.275 | .679 | 7.624 |
|  | Taking ACEi, ARNi, or ARB Yes:1 No:0(1) | -1.129 | .673 | 2.813 | 1 | .094 | .323 | .086 | 1.210 |
|  | Left ventricular ejection fraction <50% Yes:1 No:0(1) | 1.314 | .601 | 4.778 | 1 | .029 | 3.719 | 1.145 | 12.078 |
|  | Hyponatremia on admission Yes:1 No:0(1) | 1.543 | .587 | 6.914 | 1 | .009 | 4.677 | 1.481 | 14.770 |
|  | Constant | -3.642 | .688 | 27.981 | 1 | <.001 | .026 |  |  |
| Step 12^a^ | SEX Female: 1 Male:0(1) | .900 | .557 | 2.604 | 1 | .107 | 2.459 | .824 | 7.331 |
|  | Current Smoker Yes:1 No: 0(1) | -1.769 | .839 | 4.445 | 1 | .035 | .171 | .033 | .883 |
|  | History of Diabetes Yes:1 No:0(1) | -.964 | .612 | 2.479 | 1 | .115 | .381 | .115 | 1.266 |
|  | History of Stroke Yes:1 No:0(1) | 2.195 | .764 | 8.252 | 1 | .004 | 8.982 | 2.009 | 40.167 |
|  | History of CABG Yes:1 No:0(1) | 1.516 | .672 | 5.089 | 1 | .024 | 4.556 | 1.220 | 17.010 |
|  | Taking ACEi, ARNi, or ARB Yes:1 No:0(1) | -1.255 | .669 | 3.521 | 1 | .061 | .285 | .077 | 1.058 |
|  | Left ventricular ejection fraction <50% Yes:1 No:0(1) | 1.279 | .592 | 4.667 | 1 | .031 | 3.594 | 1.126 | 11.475 |
|  | Hyponatremia on admission Yes:1 No:0(1) | 1.496 | .573 | 6.823 | 1 | .009 | 4.466 | 1.453 | 13.725 |
|  | Constant | -3.438 | .646 | 28.313 | 1 | <.001 | .032 |  |  |
| Step 13^a^ | SEX Female: 1 Male:0(1) | .848 | .546 | 2.413 | 1 | .120 | 2.334 | .801 | 6.802 |
|  | Current Smoker Yes:1 No: 0(1) | -1.581 | .818 | 3.736 | 1 | .053 | .206 | .041 | 1.022 |
|  | History of Stroke Yes:1 No:0(1) | 2.076 | .756 | 7.538 | 1 | .006 | 7.974 | 1.811 | 35.103 |
|  | History of CABG Yes:1 No:0(1) | 1.192 | .620 | 3.698 | 1 | .054 | 3.292 | .977 | 11.090 |
|  | Taking ACEi, ARNi, or ARB Yes:1 No:0(1) | -1.447 | .663 | 4.762 | 1 | .029 | .235 | .064 | .863 |
|  | Left ventricular ejection fraction <50% Yes:1 No:0(1) | 1.249 | .588 | 4.505 | 1 | .034 | 3.486 | 1.100 | 11.042 |
|  | Hyponatremia on admission Yes:1 No:0(1) | 1.511 | .568 | 7.062 | 1 | .008 | 4.530 | 1.487 | 13.803 |
|  | Constant | -3.689 | .641 | 33.094 | 1 | <.001 | .025 |  |  |
| Step 14^a^ | Current Smoker Yes:1 No: 0(1) | -1.627 | .814 | 3.999 | 1 | .046 | .196 | .040 | .968 |
|  | History of Stroke Yes:1 No:0(1) | 2.203 | .758 | 8.441 | 1 | .004 | 9.056 | 2.048 | 40.041 |
|  | History of CABG Yes:1 No:0(1) | 1.061 | .600 | 3.127 | 1 | .077 | 2.890 | .891 | 9.374 |
|  | Taking ACEi, ARNi, or ARB Yes:1 No:0(1) | -1.301 | .637 | 4.170 | 1 | .041 | .272 | .078 | .949 |
|  | Left ventricular ejection fraction <50% Yes:1 No:0(1) | 1.163 | .582 | 3.995 | 1 | .046 | 3.198 | 1.023 | 10.000 |
|  | Hyponatremia on admission Yes:1 No:0(1) | 1.503 | .556 | 7.319 | 1 | .007 | 4.496 | 1.513 | 13.361 |
|  | Constant | -3.259 | .555 | 34.431 | 1 | <.001 | .038 |  |  |
| a. Variable(s) entered on step 1: SEX Female: 1 Male:0, Age 1<65 2:≥ 65, Current Smoker Yes:1 No: 0, History of HTN Yes:1 No:0, History of Diabetes Yes:1 No:0, History of Stroke Yes:1 No:0, History of Transient Ischemic Attack Yes:1 No:0, History of Peripheral Vascular Disease Yes:1 No:0, History of Cancer Yes:1 No:0, History of Myocardial Infarction Yes:1 No:0, History of Percutaneous Coronary Intervention Yes:1 No:0, History of CABG Yes:1 No:0, History of CKD Yes:1 No:0, Taking ACEi, ARNi, or ARB Yes:1 No:0, Taking Diuretics Yes:1 No:0, Heart failure diagnosis (0=No HF, 1= History of HF, 2=New HF), Left ventricular ejection fraction <50% Yes:1 No:0, In patient diuretic use, Hyponatremia on admission Yes:1 No:0. | | | | | | | | | |

**Dependent variable: EF<50%**

**Independent variable: Hyponatremia on admission**

| **Omnibus Tests of Model Coefficients** | | | | |
| --- | --- | --- | --- | --- |
|  | | Chi-square | df | Sig. |
| Step 1 | Step | 91.922 | 19 | <.001 |
|  | Block | 91.922 | 19 | <.001 |
|  | Model | 91.922 | 19 | <.001 |
| Step 2^a^ | Step | -.001 | 1 | .979 |
|  | Block | 91.921 | 18 | <.001 |
|  | Model | 91.921 | 18 | <.001 |
| Step 3^a^ | Step | -.015 | 1 | .904 |
|  | Block | 91.907 | 17 | <.001 |
|  | Model | 91.907 | 17 | <.001 |
| Step 4^a^ | Step | -.042 | 1 | .838 |
|  | Block | 91.865 | 16 | <.001 |
|  | Model | 91.865 | 16 | <.001 |
| Step 5^a^ | Step | -.083 | 1 | .774 |
|  | Block | 91.782 | 15 | <.001 |
|  | Model | 91.782 | 15 | <.001 |
| Step 6^a^ | Step | -.261 | 1 | .610 |
|  | Block | 91.521 | 14 | <.001 |
|  | Model | 91.521 | 14 | <.001 |
| Step 7^a^ | Step | -.263 | 1 | .608 |
|  | Block | 91.258 | 13 | <.001 |
|  | Model | 91.258 | 13 | <.001 |
| Step 8^a^ | Step | -.480 | 1 | .488 |
|  | Block | 90.778 | 12 | <.001 |
|  | Model | 90.778 | 12 | <.001 |
| Step 9^a^ | Step | -.658 | 1 | .417 |
|  | Block | 90.121 | 11 | <.001 |
|  | Model | 90.121 | 11 | <.001 |
| Step 10^a^ | Step | -.859 | 1 | .354 |
|  | Block | 89.262 | 10 | <.001 |
|  | Model | 89.262 | 10 | <.001 |
| Step 11^a^ | Step | -1.462 | 1 | .227 |
|  | Block | 87.800 | 9 | <.001 |
|  | Model | 87.800 | 9 | <.001 |
| Step 12^a^ | Step | -1.636 | 1 | .201 |
|  | Block | 86.164 | 8 | <.001 |
|  | Model | 86.164 | 8 | <.001 |
| a. A negative Chi-squares value indicates that the Chi-squares value has decreased from the previous step. | | | | |

| **Hosmer and Lemeshow Test** | | | |
| --- | --- | --- | --- |
| Step | Chi-square | df | Sig. |
| 1 | 3.075 | 8 | .930 |
| 2 | 3.073 | 8 | .930 |
| 3 | 2.840 | 8 | .944 |
| 4 | 3.235 | 8 | .919 |
| 5 | 2.377 | 8 | .967 |
| 6 | 5.949 | 8 | .653 |
| 7 | 7.008 | 8 | .536 |
| 8 | 4.970 | 8 | .761 |
| 9 | 4.269 | 8 | .832 |
| 10 | 10.408 | 8 | .238 |
| 11 | 4.171 | 8 | .841 |
| 12 | 5.978 | 8 | .650 |

| **Classification Table**^a^ | | | | | |
| --- | --- | --- | --- | --- | --- |
|  | Observed | | Predicted | | |
|  |  |  | Left ventricular ejection fraction <50% Yes:1 No:0 | | Percentage Correct |
|  |  |  | ≥50 | <50 |  |
| Step 1 | Left ventricular ejection fraction <50% Yes:1 No:0 | ≥50 | 107 | 20 | 84.3 |
|  |  | <50 | 25 | 69 | 73.4 |
|  | Overall Percentage | |  |  | 79.6 |
| Step 2 | Left ventricular ejection fraction <50% Yes:1 No:0 | ≥50 | 107 | 20 | 84.3 |
|  |  | <50 | 25 | 69 | 73.4 |
|  | Overall Percentage | |  |  | 79.6 |
| Step 3 | Left ventricular ejection fraction <50% Yes:1 No:0 | ≥50 | 107 | 20 | 84.3 |
|  |  | <50 | 26 | 68 | 72.3 |
|  | Overall Percentage | |  |  | 79.2 |
| Step 4 | Left ventricular ejection fraction <50% Yes:1 No:0 | ≥50 | 107 | 20 | 84.3 |
|  |  | <50 | 25 | 69 | 73.4 |
|  | Overall Percentage | |  |  | 79.6 |
| Step 5 | Left ventricular ejection fraction <50% Yes:1 No:0 | ≥50 | 108 | 19 | 85.0 |
|  |  | <50 | 26 | 68 | 72.3 |
|  | Overall Percentage | |  |  | 79.6 |
| Step 6 | Left ventricular ejection fraction <50% Yes:1 No:0 | ≥50 | 107 | 20 | 84.3 |
|  |  | <50 | 26 | 68 | 72.3 |
|  | Overall Percentage | |  |  | 79.2 |
| Step 7 | Left ventricular ejection fraction <50% Yes:1 No:0 | ≥50 | 107 | 20 | 84.3 |
|  |  | <50 | 26 | 68 | 72.3 |
|  | Overall Percentage | |  |  | 79.2 |
| Step 8 | Left ventricular ejection fraction <50% Yes:1 No:0 | ≥50 | 108 | 19 | 85.0 |
|  |  | <50 | 26 | 68 | 72.3 |
|  | Overall Percentage | |  |  | 79.6 |
| Step 9 | Left ventricular ejection fraction <50% Yes:1 No:0 | ≥50 | 108 | 19 | 85.0 |
|  |  | <50 | 27 | 67 | 71.3 |
|  | Overall Percentage | |  |  | 79.2 |
| Step 10 | Left ventricular ejection fraction <50% Yes:1 No:0 | ≥50 | 108 | 19 | 85.0 |
|  |  | <50 | 24 | 70 | 74.5 |
|  | Overall Percentage | |  |  | 80.5 |
| Step 11 | Left ventricular ejection fraction <50% Yes:1 No:0 | ≥50 | 106 | 21 | 83.5 |
|  |  | <50 | 29 | 65 | 69.1 |
|  | Overall Percentage | |  |  | 77.4 |
| Step 12 | Left ventricular ejection fraction <50% Yes:1 No:0 | ≥50 | 105 | 22 | 82.7 |
|  |  | <50 | 27 | 67 | 71.3 |
|  | Overall Percentage | |  |  | 77.8 |
| a. The cut value is .500 | | | | | |

| **Variables in the Equation** | | | | | | | | | |
| --- | --- | --- | --- | --- | --- | --- | --- | --- | --- |
|  | | B | S.E. | Wald | df | Sig. | Exp(B) | 95% C.I.for EXP(B) | |
|  |  |  |  |  |  |  |  | Lower | Upper |
| Step 1^a^ | SEX Female: 1 Male:0(1) | -.990 | .382 | 6.711 | 1 | .010 | .372 | .176 | .786 |
|  | Age 1<65 2:≥ 65(1) | .389 | .380 | 1.049 | 1 | .306 | 1.476 | .701 | 3.111 |
|  | Current Smoker Yes:1 No: 0(1) | .665 | .436 | 2.323 | 1 | .128 | 1.944 | .827 | 4.569 |
|  | History of HTN Yes:1 No:0(1) | -1.426 | .487 | 8.585 | 1 | .003 | .240 | .093 | .624 |
|  | History of Diabetes Yes:1 No:0(1) | .195 | .383 | .258 | 1 | .611 | 1.215 | .573 | 2.574 |
|  | History of Stroke Yes:1 No:0(1) | -1.777 | .830 | 4.588 | 1 | .032 | .169 | .033 | .860 |
|  | History of Transient Ischemic Attack Yes:1 No:0(1) | -18.548 | 28165.165 | .000 | 1 | .999 | .000 | .000 | . |
|  | History of Peripheral Vascular Disease Yes:1 No:0(1) | .016 | .610 | .001 | 1 | .979 | 1.016 | .307 | 3.360 |
|  | History of Cancer Yes:1 No:0(1) | .324 | .437 | .552 | 1 | .458 | 1.383 | .588 | 3.255 |
|  | History of Myocardial Infarction Yes:1 No:0(1) | .103 | .496 | .043 | 1 | .835 | 1.109 | .419 | 2.932 |
|  | History of Percutaneous Coronary Intervention Yes:1 No:0(1) | .518 | .477 | 1.180 | 1 | .277 | 1.678 | .659 | 4.271 |
|  | History of CABG Yes:1 No:0(1) | .547 | .593 | .849 | 1 | .357 | 1.727 | .540 | 5.526 |
|  | History of CKD Yes:1 No:0(1) | -.138 | .488 | .080 | 1 | .777 | .871 | .335 | 2.267 |
|  | Taking ACEi, ARNi, or ARB Yes:1 No:0(1) | .048 | .391 | .015 | 1 | .903 | 1.049 | .488 | 2.255 |
|  | Taking Diuretics Yes:1 No:0(1) | -.362 | .429 | .711 | 1 | .399 | .696 | .300 | 1.615 |
|  | Heart failure diagnosis (0=No HF, 1= History of HF, 2=New HF) |  |  | 30.518 | 2 | <.001 |  |  |  |
|  | Heart failure diagnosis (0=No HF, 1= History of HF, 2=New HF)(1) | .753 | .492 | 2.346 | 1 | .126 | 2.123 | .810 | 5.565 |
|  | Heart failure diagnosis (0=No HF, 1= History of HF, 2=New HF)(2) | 2.680 | .485 | 30.517 | 1 | <.001 | 14.581 | 5.635 | 37.731 |
|  | In patient diuretic use(1) | 1.292 | .384 | 11.340 | 1 | <.001 | 3.641 | 1.716 | 7.725 |
|  | Hyponatremia on admission Yes:1 No:0(1) | .852 | .429 | 3.935 | 1 | .047 | 2.344 | 1.010 | 5.437 |
|  | Constant | -.928 | .493 | 3.540 | 1 | .060 | .395 |  |  |
| Step 2^a^ | SEX Female: 1 Male:0(1) | -.990 | .381 | 6.758 | 1 | .009 | .371 | .176 | .784 |
|  | Age 1<65 2:≥ 65(1) | .388 | .378 | 1.055 | 1 | .304 | 1.475 | .703 | 3.094 |
|  | Current Smoker Yes:1 No: 0(1) | .665 | .435 | 2.340 | 1 | .126 | 1.945 | .829 | 4.563 |
|  | History of HTN Yes:1 No:0(1) | -1.425 | .486 | 8.602 | 1 | .003 | .241 | .093 | .623 |
|  | History of Diabetes Yes:1 No:0(1) | .196 | .379 | .267 | 1 | .605 | 1.217 | .578 | 2.559 |
|  | History of Stroke Yes:1 No:0(1) | -1.777 | .829 | 4.592 | 1 | .032 | .169 | .033 | .859 |
|  | History of Transient Ischemic Attack Yes:1 No:0(1) | -18.536 | 28165.429 | .000 | 1 | .999 | .000 | .000 | . |
|  | History of Cancer Yes:1 No:0(1) | .326 | .434 | .561 | 1 | .454 | 1.385 | .591 | 3.245 |
|  | History of Myocardial Infarction Yes:1 No:0(1) | .103 | .496 | .043 | 1 | .836 | 1.108 | .419 | 2.929 |
|  | History of Percutaneous Coronary Intervention Yes:1 No:0(1) | .519 | .474 | 1.201 | 1 | .273 | 1.681 | .664 | 4.253 |
|  | History of CABG Yes:1 No:0(1) | .549 | .585 | .882 | 1 | .348 | 1.732 | .550 | 5.451 |
|  | History of CKD Yes:1 No:0(1) | -.138 | .488 | .081 | 1 | .777 | .871 | .335 | 2.266 |
|  | Taking ACEi, ARNi, or ARB Yes:1 No:0(1) | .047 | .390 | .015 | 1 | .904 | 1.048 | .488 | 2.251 |
|  | Taking Diuretics Yes:1 No:0(1) | -.361 | .429 | .711 | 1 | .399 | .697 | .301 | 1.614 |
|  | Heart failure diagnosis (0=No HF, 1= History of HF, 2=New HF) |  |  | 30.733 | 2 | <.001 |  |  |  |
|  | Heart failure diagnosis (0=No HF, 1= History of HF, 2=New HF)(1) | .754 | .491 | 2.357 | 1 | .125 | 2.125 | .812 | 5.562 |
|  | Heart failure diagnosis (0=No HF, 1= History of HF, 2=New HF)(2) | 2.681 | .484 | 30.733 | 1 | <.001 | 14.596 | 5.658 | 37.658 |
|  | In patient diuretic use(1) | 1.292 | .383 | 11.374 | 1 | <.001 | 3.639 | 1.718 | 7.709 |
|  | Hyponatremia on admission Yes:1 No:0(1) | .851 | .429 | 3.935 | 1 | .047 | 2.343 | 1.010 | 5.434 |
|  | Constant | -.927 | .493 | 3.541 | 1 | .060 | .396 |  |  |
| Step 3^a^ | SEX Female: 1 Male:0(1) | -.986 | .379 | 6.758 | 1 | .009 | .373 | .177 | .784 |
|  | Age 1<65 2:≥ 65(1) | .392 | .377 | 1.082 | 1 | .298 | 1.480 | .707 | 3.098 |
|  | Current Smoker Yes:1 No: 0(1) | .662 | .434 | 2.326 | 1 | .127 | 1.938 | .828 | 4.535 |
|  | History of HTN Yes:1 No:0(1) | -1.414 | .477 | 8.797 | 1 | .003 | .243 | .096 | .619 |
|  | History of Diabetes Yes:1 No:0(1) | .200 | .378 | .279 | 1 | .598 | 1.221 | .582 | 2.562 |
|  | History of Stroke Yes:1 No:0(1) | -1.775 | .830 | 4.572 | 1 | .033 | .169 | .033 | .863 |
|  | History of Transient Ischemic Attack Yes:1 No:0(1) | -18.557 | 28167.860 | .000 | 1 | .999 | .000 | .000 | . |
|  | History of Cancer Yes:1 No:0(1) | .319 | .431 | .548 | 1 | .459 | 1.375 | .591 | 3.199 |
|  | History of Myocardial Infarction Yes:1 No:0(1) | .102 | .496 | .042 | 1 | .838 | 1.107 | .419 | 2.925 |
|  | History of Percutaneous Coronary Intervention Yes:1 No:0(1) | .523 | .473 | 1.224 | 1 | .268 | 1.687 | .668 | 4.260 |
|  | History of CABG Yes:1 No:0(1) | .543 | .583 | .869 | 1 | .351 | 1.722 | .549 | 5.396 |
|  | History of CKD Yes:1 No:0(1) | -.147 | .484 | .092 | 1 | .762 | .864 | .335 | 2.229 |
|  | Taking Diuretics Yes:1 No:0(1) | -.354 | .424 | .697 | 1 | .404 | .702 | .306 | 1.612 |
|  | Heart failure diagnosis (0=No HF, 1= History of HF, 2=New HF) |  |  | 30.769 | 2 | <.001 |  |  |  |
|  | Heart failure diagnosis (0=No HF, 1= History of HF, 2=New HF)(1) | .758 | .490 | 2.398 | 1 | .122 | 2.135 | .817 | 5.573 |
|  | Heart failure diagnosis (0=No HF, 1= History of HF, 2=New HF)(2) | 2.682 | .484 | 30.769 | 1 | <.001 | 14.618 | 5.666 | 37.712 |
|  | In patient diuretic use(1) | 1.292 | .383 | 11.404 | 1 | <.001 | 3.641 | 1.720 | 7.710 |
|  | Hyponatremia on admission Yes:1 No:0(1) | .855 | .428 | 3.996 | 1 | .046 | 2.352 | 1.017 | 5.441 |
|  | Constant | -.925 | .493 | 3.526 | 1 | .060 | .397 |  |  |
| Step 4^a^ | SEX Female: 1 Male:0(1) | -.997 | .376 | 7.020 | 1 | .008 | .369 | .177 | .771 |
|  | Age 1<65 2:≥ 65(1) | .392 | .377 | 1.085 | 1 | .298 | 1.481 | .707 | 3.099 |
|  | Current Smoker Yes:1 No: 0(1) | .675 | .429 | 2.479 | 1 | .115 | 1.964 | .848 | 4.549 |
|  | History of HTN Yes:1 No:0(1) | -1.422 | .475 | 8.964 | 1 | .003 | .241 | .095 | .612 |
|  | History of Diabetes Yes:1 No:0(1) | .207 | .377 | .302 | 1 | .582 | 1.230 | .588 | 2.573 |
|  | History of Stroke Yes:1 No:0(1) | -1.804 | .821 | 4.832 | 1 | .028 | .165 | .033 | .822 |
|  | History of Transient Ischemic Attack Yes:1 No:0(1) | -18.514 | 28159.584 | .000 | 1 | .999 | .000 | .000 | . |
|  | History of Cancer Yes:1 No:0(1) | .321 | .431 | .556 | 1 | .456 | 1.379 | .593 | 3.206 |
|  | History of Percutaneous Coronary Intervention Yes:1 No:0(1) | .565 | .427 | 1.752 | 1 | .186 | 1.759 | .762 | 4.058 |
|  | History of CABG Yes:1 No:0(1) | .557 | .579 | .927 | 1 | .336 | 1.746 | .561 | 5.433 |
|  | History of CKD Yes:1 No:0(1) | -.138 | .482 | .082 | 1 | .774 | .871 | .339 | 2.239 |
|  | Taking Diuretics Yes:1 No:0(1) | -.348 | .423 | .678 | 1 | .410 | .706 | .308 | 1.617 |
|  | Heart failure diagnosis (0=No HF, 1= History of HF, 2=New HF) |  |  | 30.801 | 2 | <.001 |  |  |  |
|  | Heart failure diagnosis (0=No HF, 1= History of HF, 2=New HF)(1) | .775 | .483 | 2.576 | 1 | .108 | 2.170 | .843 | 5.586 |
|  | Heart failure diagnosis (0=No HF, 1= History of HF, 2=New HF)(2) | 2.685 | .484 | 30.796 | 1 | <.001 | 14.655 | 5.678 | 37.828 |
|  | In patient diuretic use(1) | 1.295 | .383 | 11.448 | 1 | <.001 | 3.649 | 1.724 | 7.725 |
|  | Hyponatremia on admission Yes:1 No:0(1) | .853 | .427 | 3.982 | 1 | .046 | 2.346 | 1.015 | 5.421 |
|  | Constant | -.919 | .492 | 3.493 | 1 | .062 | .399 |  |  |
| Step 5^a^ | SEX Female: 1 Male:0(1) | -.996 | .376 | 7.006 | 1 | .008 | .369 | .177 | .772 |
|  | Age 1<65 2:≥ 65(1) | .385 | .376 | 1.050 | 1 | .306 | 1.470 | .704 | 3.070 |
|  | Current Smoker Yes:1 No: 0(1) | .687 | .427 | 2.591 | 1 | .107 | 1.988 | .861 | 4.587 |
|  | History of HTN Yes:1 No:0(1) | -1.431 | .474 | 9.111 | 1 | .003 | .239 | .094 | .605 |
|  | History of Diabetes Yes:1 No:0(1) | .190 | .372 | .261 | 1 | .609 | 1.209 | .583 | 2.506 |
|  | History of Stroke Yes:1 No:0(1) | -1.804 | .819 | 4.856 | 1 | .028 | .165 | .033 | .819 |
|  | History of Transient Ischemic Attack Yes:1 No:0(1) | -18.626 | 28131.373 | .000 | 1 | .999 | .000 | .000 | . |
|  | History of Cancer Yes:1 No:0(1) | .320 | .430 | .553 | 1 | .457 | 1.377 | .593 | 3.199 |
|  | History of Percutaneous Coronary Intervention Yes:1 No:0(1) | .582 | .422 | 1.896 | 1 | .168 | 1.789 | .782 | 4.093 |
|  | History of CABG Yes:1 No:0(1) | .522 | .564 | .856 | 1 | .355 | 1.686 | .558 | 5.096 |
|  | Taking Diuretics Yes:1 No:0(1) | -.356 | .422 | .713 | 1 | .398 | .700 | .307 | 1.601 |
|  | Heart failure diagnosis (0=No HF, 1= History of HF, 2=New HF) |  |  | 30.766 | 2 | <.001 |  |  |  |
|  | Heart failure diagnosis (0=No HF, 1= History of HF, 2=New HF)(1) | .746 | .470 | 2.511 | 1 | .113 | 2.108 | .838 | 5.299 |
|  | Heart failure diagnosis (0=No HF, 1= History of HF, 2=New HF)(2) | 2.677 | .483 | 30.757 | 1 | <.001 | 14.536 | 5.645 | 37.435 |
|  | In patient diuretic use(1) | 1.296 | .382 | 11.475 | 1 | <.001 | 3.653 | 1.726 | 7.731 |
|  | Hyponatremia on admission Yes:1 No:0(1) | .862 | .426 | 4.097 | 1 | .043 | 2.368 | 1.028 | 5.454 |
|  | Constant | -.925 | .491 | 3.544 | 1 | .060 | .397 |  |  |
| Step 6^a^ | SEX Female: 1 Male:0(1) | -.976 | .374 | 6.805 | 1 | .009 | .377 | .181 | .785 |
|  | Age 1<65 2:≥ 65(1) | .389 | .375 | 1.076 | 1 | .300 | 1.475 | .707 | 3.077 |
|  | Current Smoker Yes:1 No: 0(1) | .655 | .421 | 2.421 | 1 | .120 | 1.925 | .844 | 4.395 |
|  | History of HTN Yes:1 No:0(1) | -1.399 | .469 | 8.905 | 1 | .003 | .247 | .099 | .619 |
|  | History of Stroke Yes:1 No:0(1) | -1.818 | .824 | 4.875 | 1 | .027 | .162 | .032 | .815 |
|  | History of Transient Ischemic Attack Yes:1 No:0(1) | -18.527 | 28135.077 | .000 | 1 | .999 | .000 | .000 | . |
|  | History of Cancer Yes:1 No:0(1) | .291 | .427 | .462 | 1 | .497 | 1.337 | .579 | 3.090 |
|  | History of Percutaneous Coronary Intervention Yes:1 No:0(1) | .597 | .422 | 2.001 | 1 | .157 | 1.816 | .795 | 4.150 |
|  | History of CABG Yes:1 No:0(1) | .550 | .562 | .958 | 1 | .328 | 1.733 | .576 | 5.213 |
|  | Taking Diuretics Yes:1 No:0(1) | -.345 | .421 | .671 | 1 | .413 | .708 | .311 | 1.616 |
|  | Heart failure diagnosis (0=No HF, 1= History of HF, 2=New HF) |  |  | 30.860 | 2 | <.001 |  |  |  |
|  | Heart failure diagnosis (0=No HF, 1= History of HF, 2=New HF)(1) | .758 | .470 | 2.605 | 1 | .107 | 2.135 | .850 | 5.364 |
|  | Heart failure diagnosis (0=No HF, 1= History of HF, 2=New HF)(2) | 2.673 | .481 | 30.848 | 1 | <.001 | 14.483 | 5.639 | 37.198 |
|  | In patient diuretic use(1) | 1.305 | .382 | 11.680 | 1 | <.001 | 3.687 | 1.745 | 7.793 |
|  | Hyponatremia on admission Yes:1 No:0(1) | .868 | .426 | 4.147 | 1 | .042 | 2.381 | 1.033 | 5.489 |
|  | Constant | -.886 | .484 | 3.346 | 1 | .067 | .412 |  |  |
| Step 7^a^ | SEX Female: 1 Male:0(1) | -.984 | .374 | 6.913 | 1 | .009 | .374 | .180 | .778 |
|  | Age 1<65 2:≥ 65(1) | .399 | .375 | 1.137 | 1 | .286 | 1.491 | .716 | 3.107 |
|  | Current Smoker Yes:1 No: 0(1) | .651 | .421 | 2.397 | 1 | .122 | 1.918 | .841 | 4.374 |
|  | History of HTN Yes:1 No:0(1) | -1.406 | .469 | 8.979 | 1 | .003 | .245 | .098 | .615 |
|  | History of Stroke Yes:1 No:0(1) | -1.844 | .817 | 5.091 | 1 | .024 | .158 | .032 | .785 |
|  | History of Cancer Yes:1 No:0(1) | .296 | .428 | .480 | 1 | .488 | 1.345 | .582 | 3.110 |
|  | History of Percutaneous Coronary Intervention Yes:1 No:0(1) | .585 | .421 | 1.926 | 1 | .165 | 1.795 | .786 | 4.100 |
|  | History of CABG Yes:1 No:0(1) | .563 | .562 | 1.005 | 1 | .316 | 1.756 | .584 | 5.281 |
|  | Taking Diuretics Yes:1 No:0(1) | -.362 | .419 | .748 | 1 | .387 | .696 | .306 | 1.582 |
|  | Heart failure diagnosis (0=No HF, 1= History of HF, 2=New HF) |  |  | 31.063 | 2 | <.001 |  |  |  |
|  | Heart failure diagnosis (0=No HF, 1= History of HF, 2=New HF)(1) | .757 | .470 | 2.600 | 1 | .107 | 2.132 | .849 | 5.353 |
|  | Heart failure diagnosis (0=No HF, 1= History of HF, 2=New HF)(2) | 2.684 | .482 | 31.053 | 1 | <.001 | 14.637 | 5.696 | 37.614 |
|  | In patient diuretic use(1) | 1.320 | .381 | 12.014 | 1 | <.001 | 3.742 | 1.774 | 7.893 |
|  | Hyponatremia on admission Yes:1 No:0(1) | .871 | .427 | 4.173 | 1 | .041 | 2.390 | 1.036 | 5.516 |
|  | Constant | -.889 | .484 | 3.370 | 1 | .066 | .411 |  |  |
| Step 8^a^ | SEX Female: 1 Male:0(1) | -.962 | .373 | 6.665 | 1 | .010 | .382 | .184 | .793 |
|  | Age 1<65 2:≥ 65(1) | .430 | .371 | 1.342 | 1 | .247 | 1.537 | .743 | 3.180 |
|  | Current Smoker Yes:1 No: 0(1) | .581 | .407 | 2.031 | 1 | .154 | 1.787 | .804 | 3.973 |
|  | History of HTN Yes:1 No:0(1) | -1.425 | .468 | 9.279 | 1 | .002 | .241 | .096 | .602 |
|  | History of Stroke Yes:1 No:0(1) | -1.805 | .820 | 4.846 | 1 | .028 | .164 | .033 | .820 |
|  | History of Percutaneous Coronary Intervention Yes:1 No:0(1) | .597 | .419 | 2.025 | 1 | .155 | 1.817 | .798 | 4.133 |
|  | History of CABG Yes:1 No:0(1) | .525 | .559 | .881 | 1 | .348 | 1.690 | .565 | 5.055 |
|  | Taking Diuretics Yes:1 No:0(1) | -.336 | .417 | .651 | 1 | .420 | .714 | .316 | 1.617 |
|  | Heart failure diagnosis (0=No HF, 1= History of HF, 2=New HF) |  |  | 30.924 | 2 | <.001 |  |  |  |
|  | Heart failure diagnosis (0=No HF, 1= History of HF, 2=New HF)(1) | .748 | .470 | 2.539 | 1 | .111 | 2.114 | .842 | 5.307 |
|  | Heart failure diagnosis (0=No HF, 1= History of HF, 2=New HF)(2) | 2.673 | .481 | 30.915 | 1 | <.001 | 14.488 | 5.646 | 37.177 |
|  | In patient diuretic use(1) | 1.308 | .379 | 11.930 | 1 | <.001 | 3.698 | 1.761 | 7.766 |
|  | Hyponatremia on admission Yes:1 No:0(1) | .916 | .422 | 4.710 | 1 | .030 | 2.499 | 1.093 | 5.715 |
|  | Constant | -.819 | .472 | 3.010 | 1 | .083 | .441 |  |  |
| Step 9^a^ | SEX Female: 1 Male:0(1) | -.999 | .369 | 7.322 | 1 | .007 | .368 | .179 | .759 |
|  | Age 1<65 2:≥ 65(1) | .430 | .371 | 1.346 | 1 | .246 | 1.538 | .743 | 3.181 |
|  | Current Smoker Yes:1 No: 0(1) | .611 | .406 | 2.266 | 1 | .132 | 1.843 | .831 | 4.083 |
|  | History of HTN Yes:1 No:0(1) | -1.484 | .464 | 10.209 | 1 | .001 | .227 | .091 | .563 |
|  | History of Stroke Yes:1 No:0(1) | -1.803 | .808 | 4.981 | 1 | .026 | .165 | .034 | .803 |
|  | History of Percutaneous Coronary Intervention Yes:1 No:0(1) | .607 | .417 | 2.123 | 1 | .145 | 1.835 | .811 | 4.153 |
|  | History of CABG Yes:1 No:0(1) | .509 | .550 | .855 | 1 | .355 | 1.663 | .566 | 4.888 |
|  | Heart failure diagnosis (0=No HF, 1= History of HF, 2=New HF) |  |  | 30.919 | 2 | <.001 |  |  |  |
|  | Heart failure diagnosis (0=No HF, 1= History of HF, 2=New HF)(1) | .655 | .455 | 2.074 | 1 | .150 | 1.924 | .790 | 4.690 |
|  | Heart failure diagnosis (0=No HF, 1= History of HF, 2=New HF)(2) | 2.655 | .477 | 30.919 | 1 | <.001 | 14.219 | 5.578 | 36.245 |
|  | In patient diuretic use(1) | 1.270 | .374 | 11.528 | 1 | <.001 | 3.560 | 1.711 | 7.409 |
|  | Hyponatremia on admission Yes:1 No:0(1) | .946 | .420 | 5.082 | 1 | .024 | 2.576 | 1.132 | 5.866 |
|  | Constant | -.826 | .471 | 3.075 | 1 | .080 | .438 |  |  |
| Step 10^a^ | SEX Female: 1 Male:0(1) | -1.038 | .367 | 8.016 | 1 | .005 | .354 | .173 | .726 |
|  | Age 1<65 2:≥ 65(1) | .444 | .369 | 1.447 | 1 | .229 | 1.559 | .756 | 3.213 |
|  | Current Smoker Yes:1 No: 0(1) | .590 | .406 | 2.112 | 1 | .146 | 1.805 | .814 | 4.001 |
|  | History of HTN Yes:1 No:0(1) | -1.451 | .464 | 9.762 | 1 | .002 | .234 | .094 | .582 |
|  | History of Stroke Yes:1 No:0(1) | -1.765 | .802 | 4.842 | 1 | .028 | .171 | .036 | .825 |
|  | History of Percutaneous Coronary Intervention Yes:1 No:0(1) | .709 | .399 | 3.157 | 1 | .076 | 2.032 | .929 | 4.444 |
|  | Heart failure diagnosis (0=No HF, 1= History of HF, 2=New HF) |  |  | 31.407 | 2 | <.001 |  |  |  |
|  | Heart failure diagnosis (0=No HF, 1= History of HF, 2=New HF)(1) | .753 | .442 | 2.905 | 1 | .088 | 2.124 | .893 | 5.052 |
|  | Heart failure diagnosis (0=No HF, 1= History of HF, 2=New HF)(2) | 2.662 | .475 | 31.373 | 1 | <.001 | 14.328 | 5.644 | 36.373 |
|  | In patient diuretic use(1) | 1.279 | .375 | 11.628 | 1 | <.001 | 3.594 | 1.723 | 7.498 |
|  | Hyponatremia on admission Yes:1 No:0(1) | .976 | .417 | 5.482 | 1 | .019 | 2.655 | 1.172 | 6.010 |
|  | Constant | -.837 | .472 | 3.150 | 1 | .076 | .433 |  |  |
| Step 11^a^ | SEX Female: 1 Male:0(1) | -.978 | .361 | 7.354 | 1 | .007 | .376 | .185 | .762 |
|  | Current Smoker Yes:1 No: 0(1) | .512 | .401 | 1.631 | 1 | .202 | 1.669 | .760 | 3.663 |
|  | History of HTN Yes:1 No:0(1) | -1.356 | .452 | 9.007 | 1 | .003 | .258 | .106 | .625 |
|  | History of Stroke Yes:1 No:0(1) | -1.672 | .799 | 4.380 | 1 | .036 | .188 | .039 | .899 |
|  | History of Percutaneous Coronary Intervention Yes:1 No:0(1) | .681 | .398 | 2.931 | 1 | .087 | 1.975 | .906 | 4.306 |
|  | Heart failure diagnosis (0=No HF, 1= History of HF, 2=New HF) |  |  | 31.876 | 2 | <.001 |  |  |  |
|  | Heart failure diagnosis (0=No HF, 1= History of HF, 2=New HF)(1) | .843 | .433 | 3.797 | 1 | .051 | 2.324 | .995 | 5.428 |
|  | Heart failure diagnosis (0=No HF, 1= History of HF, 2=New HF)(2) | 2.677 | .475 | 31.728 | 1 | <.001 | 14.545 | 5.730 | 36.924 |
|  | In patient diuretic use(1) | 1.293 | .374 | 11.986 | 1 | <.001 | 3.645 | 1.753 | 7.579 |
|  | Hyponatremia on admission Yes:1 No:0(1) | .932 | .416 | 5.010 | 1 | .025 | 2.539 | 1.123 | 5.743 |
|  | Constant | -.697 | .453 | 2.366 | 1 | .124 | .498 |  |  |
| Step 12^a^ | SEX Female: 1 Male:0(1) | -.945 | .358 | 6.957 | 1 | .008 | .389 | .193 | .784 |
|  | History of HTN Yes:1 No:0(1) | -1.424 | .445 | 10.245 | 1 | .001 | .241 | .101 | .576 |
|  | History of Stroke Yes:1 No:0(1) | -1.647 | .781 | 4.450 | 1 | .035 | .193 | .042 | .890 |
|  | History of Percutaneous Coronary Intervention Yes:1 No:0(1) | .701 | .398 | 3.112 | 1 | .078 | 2.017 | .925 | 4.396 |
|  | Heart failure diagnosis (0=No HF, 1= History of HF, 2=New HF) |  |  | 31.594 | 2 | <.001 |  |  |  |
|  | Heart failure diagnosis (0=No HF, 1= History of HF, 2=New HF)(1) | .801 | .428 | 3.498 | 1 | .061 | 2.227 | .962 | 5.152 |
|  | Heart failure diagnosis (0=No HF, 1= History of HF, 2=New HF)(2) | 2.647 | .472 | 31.491 | 1 | <.001 | 14.108 | 5.597 | 35.556 |
|  | In patient diuretic use(1) | 1.239 | .367 | 11.385 | 1 | <.001 | 3.451 | 1.681 | 7.087 |
|  | Hyponatremia on admission Yes:1 No:0(1) | .980 | .415 | 5.574 | 1 | .018 | 2.666 | 1.181 | 6.015 |
|  | Constant | -.499 | .420 | 1.410 | 1 | .235 | .607 |  |  |
| a. Variable(s) entered on step 1: SEX Female: 1 Male:0, Age 1<65 2:≥ 65, Current Smoker Yes:1 No: 0, History of HTN Yes:1 No:0, History of Diabetes Yes:1 No:0, History of Stroke Yes:1 No:0, History of Transient Ischemic Attack Yes:1 No:0, History of Peripheral Vascular Disease Yes:1 No:0, History of Cancer Yes:1 No:0, History of Myocardial Infarction Yes:1 No:0, History of Percutaneous Coronary Intervention Yes:1 No:0, History of CABG Yes:1 No:0, History of CKD Yes:1 No:0, Taking ACEi, ARNi, or ARB Yes:1 No:0, Taking Diuretics Yes:1 No:0, Heart failure diagnosis (0=No HF, 1= History of HF, 2=New HF), In patient diuretic use, Hyponatremia on admission Yes:1 No:0. | | | | | | | | | |
