# Supplementary material for: The association of hyponatremia and clinical outcomes in patients with acute myocardial infarction: a cross-sectional study
Source: BMC Cardiovasc Disord. 2022 Jun 18;22:276. doi: 10.1186/s12872-022-02700-y (PMC9206366; doi:10.1186/s12872-022-02700-y)
Supplement: Supplementary file 2 — Additional file 2. Regression and sensitivity analysis cont'd. [file 12872_2022_2700_MOESM2_ESM.docx]

**Dependent variable: In-hospital mortality**

**Independent variable: Hyponatremia up to 7 days of admission**

| **Omnibus Tests of Model Coefficients** | | | | |
| --- | --- | --- | --- | --- |
|  | | Chi-square | df | Sig. |
| Step 1 | Step | 28.795 | 20 | .092 |
|  | Block | 28.795 | 20 | .092 |
|  | Model | 28.795 | 20 | .092 |
| Step 2^a^ | Step | -.060 | 1 | .806 |
|  | Block | 28.734 | 19 | .070 |
|  | Model | 28.734 | 19 | .070 |
| Step 3^a^ | Step | -.049 | 1 | .824 |
|  | Block | 28.685 | 18 | .052 |
|  | Model | 28.685 | 18 | .052 |
| Step 4^a^ | Step | -.150 | 1 | .698 |
|  | Block | 28.535 | 17 | .039 |
|  | Model | 28.535 | 17 | .039 |
| Step 5^a^ | Step | -.138 | 1 | .710 |
|  | Block | 28.396 | 16 | .028 |
|  | Model | 28.396 | 16 | .028 |
| Step 6^a^ | Step | -.128 | 1 | .720 |
|  | Block | 28.268 | 15 | .020 |
|  | Model | 28.268 | 15 | .020 |
| Step 7^a^ | Step | -.106 | 1 | .744 |
|  | Block | 28.161 | 14 | .014 |
|  | Model | 28.161 | 14 | .014 |
| Step 8^a^ | Step | -.126 | 1 | .723 |
|  | Block | 28.036 | 13 | .009 |
|  | Model | 28.036 | 13 | .009 |
| Step 9^a^ | Step | -.166 | 1 | .683 |
|  | Block | 27.869 | 12 | .006 |
|  | Model | 27.869 | 12 | .006 |
| Step 10^a^ | Step | -.939 | 2 | .625 |
|  | Block | 26.930 | 10 | .003 |
|  | Model | 26.930 | 11 | .005 |
| Step 11^a^ | Step | -.728 | 1 | .394 |
|  | Block | 26.202 | 9 | .002 |
|  | Model | 26.202 | 9 | .002 |
| Step 12^a^ | Step | -.684 | 1 | .408 |
|  | Block | 25.519 | 8 | .001 |
|  | Model | 25.519 | 8 | .001 |
| Step 13^a^ | Step | -1.466 | 1 | .226 |
|  | Block | 24.052 | 7 | .001 |
|  | Model | 24.052 | 7 | .001 |
| Step 14^a^ | Step | -.685 | 1 | .408 |
|  | Block | 23.367 | 6 | <.001 |
|  | Model | 23.367 | 6 | <.001 |
| Step 15^a^ | Step | -1.434 | 1 | .231 |
|  | Block | 21.933 | 5 | <.001 |
|  | Model | 21.933 | 5 | <.001 |
| Step 16^a^ | Step | -2.211 | 1 | .137 |
|  | Block | 19.722 | 4 | <.001 |
|  | Model | 19.722 | 4 | <.001 |
| Step 17^a^ | Step | -2.024 | 1 | .155 |
|  | Block | 17.698 | 3 | <.001 |
|  | Model | 17.698 | 3 | <.001 |
| a. A negative Chi-squares value indicates that the Chi-squares value has decreased from the previous step. | | | | |

| **Hosmer and Lemeshow Test** | | | |
| --- | --- | --- | --- |
| Step | Chi-square | df | Sig. |
| 1 | 2.839 | 8 | .944 |
| 2 | 2.026 | 8 | .980 |
| 3 | 2.127 | 8 | .977 |
| 4 | 2.005 | 8 | .981 |
| 5 | 3.809 | 8 | .874 |
| 6 | 6.269 | 8 | .617 |
| 7 | 6.511 | 8 | .590 |
| 8 | 3.310 | 8 | .913 |
| 9 | 5.687 | 8 | .682 |
| 10 | 2.111 | 8 | .977 |
| 11 | 6.613 | 8 | .579 |
| 12 | 6.228 | 8 | .622 |
| 13 | 5.491 | 8 | .704 |
| 14 | 3.665 | 8 | .886 |
| 15 | 2.866 | 7 | .897 |
| 16 | 1.969 | 7 | .962 |
| 17 | 1.653 | 5 | .895 |

| **Classification Table**^a^ | | | | | |
| --- | --- | --- | --- | --- | --- |
|  | Observed | | Predicted | | |
|  |  |  | In Hospital mortality Yes:1 No:0 | | Percentage Correct |
|  |  |  | no | yes |  |
| Step 1 | In Hospital mortality Yes:1 No:0 | no | 206 | 0 | 100.0 |
|  |  | yes | 15 | 0 | .0 |
|  | Overall Percentage | |  |  | 93.2 |
| Step 2 | In Hospital mortality Yes:1 No:0 | no | 206 | 0 | 100.0 |
|  |  | yes | 15 | 0 | .0 |
|  | Overall Percentage | |  |  | 93.2 |
| Step 3 | In Hospital mortality Yes:1 No:0 | no | 206 | 0 | 100.0 |
|  |  | yes | 15 | 0 | .0 |
|  | Overall Percentage | |  |  | 93.2 |
| Step 4 | In Hospital mortality Yes:1 No:0 | no | 206 | 0 | 100.0 |
|  |  | yes | 15 | 0 | .0 |
|  | Overall Percentage | |  |  | 93.2 |
| Step 5 | In Hospital mortality Yes:1 No:0 | no | 206 | 0 | 100.0 |
|  |  | yes | 15 | 0 | .0 |
|  | Overall Percentage | |  |  | 93.2 |
| Step 6 | In Hospital mortality Yes:1 No:0 | no | 206 | 0 | 100.0 |
|  |  | yes | 15 | 0 | .0 |
|  | Overall Percentage | |  |  | 93.2 |
| Step 7 | In Hospital mortality Yes:1 No:0 | no | 206 | 0 | 100.0 |
|  |  | yes | 15 | 0 | .0 |
|  | Overall Percentage | |  |  | 93.2 |
| Step 8 | In Hospital mortality Yes:1 No:0 | no | 206 | 0 | 100.0 |
|  |  | yes | 15 | 0 | .0 |
|  | Overall Percentage | |  |  | 93.2 |
| Step 9 | In Hospital mortality Yes:1 No:0 | no | 206 | 0 | 100.0 |
|  |  | yes | 14 | 1 | 6.7 |
|  | Overall Percentage | |  |  | 93.7 |
| Step 10 | In Hospital mortality Yes:1 No:0 | no | 206 | 0 | 100.0 |
|  |  | yes | 15 | 0 | .0 |
|  | Overall Percentage | |  |  | 93.2 |
| Step 11 | In Hospital mortality Yes:1 No:0 | no | 206 | 0 | 100.0 |
|  |  | yes | 15 | 0 | .0 |
|  | Overall Percentage | |  |  | 93.2 |
| Step 12 | In Hospital mortality Yes:1 No:0 | no | 206 | 0 | 100.0 |
|  |  | yes | 15 | 0 | .0 |
|  | Overall Percentage | |  |  | 93.2 |
| Step 13 | In Hospital mortality Yes:1 No:0 | no | 206 | 0 | 100.0 |
|  |  | yes | 15 | 0 | .0 |
|  | Overall Percentage | |  |  | 93.2 |
| Step 14 | In Hospital mortality Yes:1 No:0 | no | 206 | 0 | 100.0 |
|  |  | yes | 15 | 0 | .0 |
|  | Overall Percentage | |  |  | 93.2 |
| Step 15 | In Hospital mortality Yes:1 No:0 | no | 206 | 0 | 100.0 |
|  |  | yes | 15 | 0 | .0 |
|  | Overall Percentage | |  |  | 93.2 |
| Step 16 | In Hospital mortality Yes:1 No:0 | no | 206 | 0 | 100.0 |
|  |  | yes | 15 | 0 | .0 |
|  | Overall Percentage | |  |  | 93.2 |
| Step 17 | In Hospital mortality Yes:1 No:0 | no | 206 | 0 | 100.0 |
|  |  | yes | 15 | 0 | .0 |
|  | Overall Percentage | |  |  | 93.2 |
| a. The cut value is .500 | | | | | |

| **Variables in the Equation** | | | | | | | | | |
| --- | --- | --- | --- | --- | --- | --- | --- | --- | --- |
|  | | B | S.E. | Wald | df | Sig. | Exp(B) | 95% C.I.for EXP(B) | |
|  |  |  |  |  |  |  |  | Lower | Upper |
| Step 1^a^ | SEX Female: 1 Male:0(1) | .805 | .646 | 1.552 | 1 | .213 | 2.237 | .630 | 7.937 |
|  | Age 1<65 2:≥ 65(1) | .305 | .749 | .166 | 1 | .684 | 1.357 | .312 | 5.893 |
|  | Current Smoker Yes:1 No: 0(1) | -2.548 | 1.315 | 3.755 | 1 | .053 | .078 | .006 | 1.030 |
|  | History of HTN Yes:1 No:0(1) | .234 | .940 | .062 | 1 | .803 | 1.264 | .200 | 7.975 |
|  | History of Diabetes Yes:1 No:0(1) | -.717 | .725 | .978 | 1 | .323 | .488 | .118 | 2.022 |
|  | History of Stroke Yes:1 No:0(1) | 1.859 | 1.087 | 2.921 | 1 | .087 | 6.415 | .761 | 54.058 |
|  | History of Transient Ischemic Attack Yes:1 No:0(1) | -17.659 | 24220.293 | .000 | 1 | .999 | .000 | .000 | . |
|  | History of Peripheral Vascular Disease Yes:1 No:0(1) | .499 | 1.133 | .194 | 1 | .660 | 1.647 | .179 | 15.183 |
|  | History of Cancer Yes:1 No:0(1) | -.366 | .717 | .260 | 1 | .610 | .694 | .170 | 2.827 |
|  | History of Myocardial Infarction Yes:1 No:0(1) | .432 | 1.050 | .170 | 1 | .680 | 1.541 | .197 | 12.067 |
|  | History of Percutaneous Coronary Intervention Yes:1 No:0(1) | -1.436 | 1.101 | 1.702 | 1 | .192 | .238 | .027 | 2.057 |
|  | History of CABG Yes:1 No:0(1) | .952 | 1.027 | .859 | 1 | .354 | 2.590 | .346 | 19.365 |
|  | History of CKD Yes:1 No:0(1) | -.440 | .887 | .246 | 1 | .620 | .644 | .113 | 3.663 |
|  | Taking ACEi, ARNi, or ARB Yes:1 No:0(1) | -1.346 | .825 | 2.664 | 1 | .103 | .260 | .052 | 1.310 |
|  | Taking Diuretics Yes:1 No:0(1) | -.172 | .703 | .059 | 1 | .807 | .842 | .212 | 3.343 |
|  | Left ventricular ejection fraction <50% Yes:1 No:0(1) | 1.416 | .798 | 3.147 | 1 | .076 | 4.120 | .862 | 19.688 |
|  | Heart failure diagnosis (0=No HF, 1= History of HF, 2=New HF) |  |  | .836 | 2 | .658 |  |  |  |
|  | Heart failure diagnosis (0=No HF, 1= History of HF, 2=New HF)(1) | .540 | .922 | .343 | 1 | .558 | 1.716 | .282 | 10.448 |
|  | Heart failure diagnosis (0=No HF, 1= History of HF, 2=New HF)(2) | .695 | .791 | .773 | 1 | .379 | 2.004 | .425 | 9.437 |
|  | In patient diuretic use(1) | .539 | .722 | .557 | 1 | .455 | 1.715 | .416 | 7.064 |
|  | Hyponatremia present up to 7 days of hospitalization Yes:1 No:0(1) | 2.134 | 1.101 | 3.759 | 1 | .053 | 8.451 | .977 | 73.106 |
|  | Constant | -5.465 | 1.470 | 13.829 | 1 | <.001 | .004 |  |  |
| Step 2^a^ | SEX Female: 1 Male:0(1) | .792 | .644 | 1.514 | 1 | .219 | 2.209 | .625 | 7.805 |
|  | Age 1<65 2:≥ 65(1) | .270 | .735 | .135 | 1 | .714 | 1.309 | .310 | 5.528 |
|  | Current Smoker Yes:1 No: 0(1) | -2.516 | 1.308 | 3.699 | 1 | .054 | .081 | .006 | 1.049 |
|  | History of HTN Yes:1 No:0(1) | .205 | .931 | .049 | 1 | .825 | 1.228 | .198 | 7.610 |
|  | History of Diabetes Yes:1 No:0(1) | -.714 | .724 | .971 | 1 | .324 | .490 | .118 | 2.026 |
|  | History of Stroke Yes:1 No:0(1) | 1.853 | 1.090 | 2.892 | 1 | .089 | 6.377 | .754 | 53.952 |
|  | History of Transient Ischemic Attack Yes:1 No:0(1) | -17.720 | 24257.565 | .000 | 1 | .999 | .000 | .000 | . |
|  | History of Peripheral Vascular Disease Yes:1 No:0(1) | .446 | 1.111 | .161 | 1 | .688 | 1.561 | .177 | 13.786 |
|  | History of Cancer Yes:1 No:0(1) | -.343 | .707 | .236 | 1 | .627 | .709 | .177 | 2.837 |
|  | History of Myocardial Infarction Yes:1 No:0(1) | .414 | 1.040 | .158 | 1 | .691 | 1.512 | .197 | 11.599 |
|  | History of Percutaneous Coronary Intervention Yes:1 No:0(1) | -1.408 | 1.095 | 1.654 | 1 | .198 | .245 | .029 | 2.091 |
|  | History of CABG Yes:1 No:0(1) | .973 | 1.019 | .912 | 1 | .340 | 2.646 | .359 | 19.501 |
|  | History of CKD Yes:1 No:0(1) | -.416 | .882 | .222 | 1 | .637 | .660 | .117 | 3.714 |
|  | Taking ACEi, ARNi, or ARB Yes:1 No:0(1) | -1.340 | .820 | 2.674 | 1 | .102 | .262 | .053 | 1.305 |
|  | Left ventricular ejection fraction <50% Yes:1 No:0(1) | 1.422 | .795 | 3.200 | 1 | .074 | 4.146 | .873 | 19.692 |
|  | Heart failure diagnosis (0=No HF, 1= History of HF, 2=New HF) |  |  | .782 | 2 | .676 |  |  |  |
|  | Heart failure diagnosis (0=No HF, 1= History of HF, 2=New HF)(1) | .486 | .893 | .295 | 1 | .587 | 1.625 | .282 | 9.359 |
|  | Heart failure diagnosis (0=No HF, 1= History of HF, 2=New HF)(2) | .666 | .782 | .726 | 1 | .394 | 1.947 | .420 | 9.020 |
|  | In patient diuretic use(1) | .545 | .722 | .569 | 1 | .451 | 1.724 | .419 | 7.098 |
|  | Hyponatremia present up to 7 days of hospitalization Yes:1 No:0(1) | 2.120 | 1.098 | 3.730 | 1 | .053 | 8.332 | .969 | 71.643 |
|  | Constant | -5.460 | 1.471 | 13.780 | 1 | <.001 | .004 |  |  |
| Step 3^a^ | SEX Female: 1 Male:0(1) | .764 | .631 | 1.466 | 1 | .226 | 2.147 | .623 | 7.392 |
|  | Age 1<65 2:≥ 65(1) | .289 | .729 | .157 | 1 | .692 | 1.335 | .320 | 5.572 |
|  | Current Smoker Yes:1 No: 0(1) | -2.533 | 1.308 | 3.750 | 1 | .053 | .079 | .006 | 1.031 |
|  | History of Diabetes Yes:1 No:0(1) | -.701 | .721 | .945 | 1 | .331 | .496 | .121 | 2.038 |
|  | History of Stroke Yes:1 No:0(1) | 1.810 | 1.064 | 2.893 | 1 | .089 | 6.113 | .759 | 49.236 |
|  | History of Transient Ischemic Attack Yes:1 No:0(1) | -17.774 | 24261.780 | .000 | 1 | .999 | .000 | .000 | . |
|  | History of Peripheral Vascular Disease Yes:1 No:0(1) | .498 | 1.084 | .211 | 1 | .646 | 1.646 | .197 | 13.775 |
|  | History of Cancer Yes:1 No:0(1) | -.340 | .706 | .232 | 1 | .630 | .712 | .179 | 2.838 |
|  | History of Myocardial Infarction Yes:1 No:0(1) | .414 | 1.029 | .162 | 1 | .687 | 1.513 | .201 | 11.360 |
|  | History of Percutaneous Coronary Intervention Yes:1 No:0(1) | -1.360 | 1.059 | 1.648 | 1 | .199 | .257 | .032 | 2.047 |
|  | History of CABG Yes:1 No:0(1) | .969 | 1.014 | .913 | 1 | .339 | 2.635 | .361 | 19.223 |
|  | History of CKD Yes:1 No:0(1) | -.352 | .826 | .182 | 1 | .670 | .703 | .139 | 3.551 |
|  | Taking ACEi, ARNi, or ARB Yes:1 No:0(1) | -1.289 | .784 | 2.705 | 1 | .100 | .275 | .059 | 1.280 |
|  | Left ventricular ejection fraction <50% Yes:1 No:0(1) | 1.367 | .751 | 3.312 | 1 | .069 | 3.922 | .900 | 17.089 |
|  | Heart failure diagnosis (0=No HF, 1= History of HF, 2=New HF) |  |  | .824 | 2 | .662 |  |  |  |
|  | Heart failure diagnosis (0=No HF, 1= History of HF, 2=New HF)(1) | .448 | .875 | .262 | 1 | .609 | 1.565 | .282 | 8.689 |
|  | Heart failure diagnosis (0=No HF, 1= History of HF, 2=New HF)(2) | .694 | .775 | .804 | 1 | .370 | 2.003 | .439 | 9.140 |
|  | In patient diuretic use(1) | .543 | .721 | .568 | 1 | .451 | 1.722 | .419 | 7.078 |
|  | Hyponatremia present up to 7 days of hospitalization Yes:1 No:0(1) | 2.132 | 1.098 | 3.770 | 1 | .052 | 8.434 | .980 | 72.569 |
|  | Constant | -5.312 | 1.298 | 16.753 | 1 | <.001 | .005 |  |  |
| Step 4^a^ | SEX Female: 1 Male:0(1) | .775 | .631 | 1.511 | 1 | .219 | 2.171 | .631 | 7.477 |
|  | Age 1<65 2:≥ 65(1) | .308 | .728 | .179 | 1 | .673 | 1.360 | .326 | 5.671 |
|  | Current Smoker Yes:1 No: 0(1) | -2.514 | 1.305 | 3.714 | 1 | .054 | .081 | .006 | 1.044 |
|  | History of Diabetes Yes:1 No:0(1) | -.703 | .723 | .946 | 1 | .331 | .495 | .120 | 2.042 |
|  | History of Stroke Yes:1 No:0(1) | 1.774 | 1.067 | 2.767 | 1 | .096 | 5.896 | .729 | 47.704 |
|  | History of Peripheral Vascular Disease Yes:1 No:0(1) | .435 | 1.072 | .165 | 1 | .685 | 1.545 | .189 | 12.629 |
|  | History of Cancer Yes:1 No:0(1) | -.322 | .704 | .209 | 1 | .648 | .725 | .182 | 2.883 |
|  | History of Myocardial Infarction Yes:1 No:0(1) | .386 | 1.032 | .140 | 1 | .708 | 1.471 | .195 | 11.121 |
|  | History of Percutaneous Coronary Intervention Yes:1 No:0(1) | -1.395 | 1.062 | 1.725 | 1 | .189 | .248 | .031 | 1.987 |
|  | History of CABG Yes:1 No:0(1) | 1.038 | .992 | 1.096 | 1 | .295 | 2.824 | .404 | 19.724 |
|  | History of CKD Yes:1 No:0(1) | -.385 | .825 | .218 | 1 | .641 | .680 | .135 | 3.426 |
|  | Taking ACEi, ARNi, or ARB Yes:1 No:0(1) | -1.293 | .787 | 2.701 | 1 | .100 | .274 | .059 | 1.283 |
|  | Left ventricular ejection fraction <50% Yes:1 No:0(1) | 1.389 | .750 | 3.428 | 1 | .064 | 4.011 | .922 | 17.450 |
|  | Heart failure diagnosis (0=No HF, 1= History of HF, 2=New HF) |  |  | .796 | 2 | .672 |  |  |  |
|  | Heart failure diagnosis (0=No HF, 1= History of HF, 2=New HF)(1) | .423 | .876 | .233 | 1 | .629 | 1.527 | .274 | 8.506 |
|  | Heart failure diagnosis (0=No HF, 1= History of HF, 2=New HF)(2) | .685 | .774 | .782 | 1 | .376 | 1.983 | .435 | 9.041 |
|  | In patient diuretic use(1) | .555 | .722 | .591 | 1 | .442 | 1.742 | .423 | 7.176 |
|  | Hyponatremia present up to 7 days of hospitalization Yes:1 No:0(1) | 2.117 | 1.097 | 3.723 | 1 | .054 | 8.308 | .967 | 71.370 |
|  | Constant | -5.323 | 1.301 | 16.748 | 1 | <.001 | .005 |  |  |
| Step 5^a^ | SEX Female: 1 Male:0(1) | .774 | .631 | 1.503 | 1 | .220 | 2.169 | .629 | 7.477 |
|  | Age 1<65 2:≥ 65(1) | .255 | .714 | .128 | 1 | .721 | 1.291 | .319 | 5.230 |
|  | Current Smoker Yes:1 No: 0(1) | -2.476 | 1.294 | 3.660 | 1 | .056 | .084 | .007 | 1.063 |
|  | History of Diabetes Yes:1 No:0(1) | -.664 | .708 | .880 | 1 | .348 | .515 | .128 | 2.062 |
|  | History of Stroke Yes:1 No:0(1) | 1.700 | 1.044 | 2.648 | 1 | .104 | 5.471 | .707 | 42.367 |
|  | History of Peripheral Vascular Disease Yes:1 No:0(1) | .418 | 1.072 | .152 | 1 | .697 | 1.519 | .186 | 12.423 |
|  | History of Cancer Yes:1 No:0(1) | -.280 | .690 | .165 | 1 | .685 | .756 | .195 | 2.923 |
|  | History of Percutaneous Coronary Intervention Yes:1 No:0(1) | -1.203 | .917 | 1.720 | 1 | .190 | .300 | .050 | 1.812 |
|  | History of CABG Yes:1 No:0(1) | 1.077 | .991 | 1.181 | 1 | .277 | 2.935 | .421 | 20.460 |
|  | History of CKD Yes:1 No:0(1) | -.331 | .810 | .167 | 1 | .683 | .718 | .147 | 3.514 |
|  | Taking ACEi, ARNi, or ARB Yes:1 No:0(1) | -1.294 | .790 | 2.683 | 1 | .101 | .274 | .058 | 1.290 |
|  | Left ventricular ejection fraction <50% Yes:1 No:0(1) | 1.384 | .744 | 3.464 | 1 | .063 | 3.992 | .929 | 17.146 |
|  | Heart failure diagnosis (0=No HF, 1= History of HF, 2=New HF) |  |  | .735 | 2 | .692 |  |  |  |
|  | Heart failure diagnosis (0=No HF, 1= History of HF, 2=New HF)(1) | .465 | .871 | .285 | 1 | .593 | 1.593 | .289 | 8.779 |
|  | Heart failure diagnosis (0=No HF, 1= History of HF, 2=New HF)(2) | .645 | .767 | .706 | 1 | .401 | 1.905 | .424 | 8.567 |
|  | In patient diuretic use(1) | .567 | .720 | .620 | 1 | .431 | 1.762 | .430 | 7.224 |
|  | Hyponatremia present up to 7 days of hospitalization Yes:1 No:0(1) | 2.080 | 1.094 | 3.618 | 1 | .057 | 8.005 | .939 | 68.278 |
|  | Constant | -5.276 | 1.291 | 16.700 | 1 | <.001 | .005 |  |  |
| Step 6^a^ | SEX Female: 1 Male:0(1) | .785 | .630 | 1.553 | 1 | .213 | 2.193 | .638 | 7.542 |
|  | Current Smoker Yes:1 No: 0(1) | -2.585 | 1.282 | 4.068 | 1 | .044 | .075 | .006 | .930 |
|  | History of Diabetes Yes:1 No:0(1) | -.669 | .711 | .884 | 1 | .347 | .512 | .127 | 2.064 |
|  | History of Stroke Yes:1 No:0(1) | 1.745 | 1.040 | 2.819 | 1 | .093 | 5.728 | .747 | 43.942 |
|  | History of Peripheral Vascular Disease Yes:1 No:0(1) | .345 | 1.045 | .109 | 1 | .741 | 1.412 | .182 | 10.941 |
|  | History of Cancer Yes:1 No:0(1) | -.281 | .690 | .166 | 1 | .684 | .755 | .195 | 2.919 |
|  | History of Percutaneous Coronary Intervention Yes:1 No:0(1) | -1.205 | .916 | 1.732 | 1 | .188 | .300 | .050 | 1.803 |
|  | History of CABG Yes:1 No:0(1) | 1.104 | .988 | 1.248 | 1 | .264 | 3.016 | .435 | 20.917 |
|  | History of CKD Yes:1 No:0(1) | -.258 | .784 | .108 | 1 | .742 | .773 | .166 | 3.595 |
|  | Taking ACEi, ARNi, or ARB Yes:1 No:0(1) | -1.277 | .788 | 2.626 | 1 | .105 | .279 | .060 | 1.307 |
|  | Left ventricular ejection fraction <50% Yes:1 No:0(1) | 1.389 | .741 | 3.516 | 1 | .061 | 4.012 | .939 | 17.138 |
|  | Heart failure diagnosis (0=No HF, 1= History of HF, 2=New HF) |  |  | .877 | 2 | .645 |  |  |  |
|  | Heart failure diagnosis (0=No HF, 1= History of HF, 2=New HF)(1) | .499 | .866 | .332 | 1 | .564 | 1.647 | .302 | 8.993 |
|  | Heart failure diagnosis (0=No HF, 1= History of HF, 2=New HF)(2) | .692 | .754 | .842 | 1 | .359 | 1.997 | .456 | 8.747 |
|  | In patient diuretic use(1) | .623 | .705 | .781 | 1 | .377 | 1.864 | .468 | 7.419 |
|  | Hyponatremia present up to 7 days of hospitalization Yes:1 No:0(1) | 2.082 | 1.095 | 3.612 | 1 | .057 | 8.019 | .937 | 68.632 |
|  | Constant | -5.210 | 1.275 | 16.701 | 1 | <.001 | .005 |  |  |
| Step 7^a^ | SEX Female: 1 Male:0(1) | .763 | .625 | 1.490 | 1 | .222 | 2.145 | .630 | 7.309 |
|  | Current Smoker Yes:1 No: 0(1) | -2.564 | 1.276 | 4.040 | 1 | .044 | .077 | .006 | .938 |
|  | History of Diabetes Yes:1 No:0(1) | -.600 | .675 | .792 | 1 | .373 | .549 | .146 | 2.058 |
|  | History of Stroke Yes:1 No:0(1) | 1.745 | 1.039 | 2.823 | 1 | .093 | 5.726 | .748 | 43.838 |
|  | History of Cancer Yes:1 No:0(1) | -.237 | .675 | .124 | 1 | .725 | .789 | .210 | 2.960 |
|  | History of Percutaneous Coronary Intervention Yes:1 No:0(1) | -1.209 | .915 | 1.748 | 1 | .186 | .298 | .050 | 1.792 |
|  | History of CABG Yes:1 No:0(1) | 1.165 | .967 | 1.452 | 1 | .228 | 3.207 | .482 | 21.334 |
|  | History of CKD Yes:1 No:0(1) | -.302 | .778 | .151 | 1 | .698 | .739 | .161 | 3.397 |
|  | Taking ACEi, ARNi, or ARB Yes:1 No:0(1) | -1.328 | .776 | 2.930 | 1 | .087 | .265 | .058 | 1.212 |
|  | Left ventricular ejection fraction <50% Yes:1 No:0(1) | 1.396 | .742 | 3.540 | 1 | .060 | 4.039 | .943 | 17.291 |
|  | Heart failure diagnosis (0=No HF, 1= History of HF, 2=New HF) |  |  | .931 | 2 | .628 |  |  |  |
|  | Heart failure diagnosis (0=No HF, 1= History of HF, 2=New HF)(1) | .541 | .857 | .398 | 1 | .528 | 1.718 | .320 | 9.225 |
|  | Heart failure diagnosis (0=No HF, 1= History of HF, 2=New HF)(2) | .705 | .754 | .873 | 1 | .350 | 2.023 | .461 | 8.875 |
|  | In patient diuretic use(1) | .607 | .701 | .750 | 1 | .387 | 1.835 | .464 | 7.249 |
|  | Hyponatremia present up to 7 days of hospitalization Yes:1 No:0(1) | 2.085 | 1.094 | 3.633 | 1 | .057 | 8.048 | .943 | 68.711 |
|  | Constant | -5.207 | 1.275 | 16.686 | 1 | <.001 | .005 |  |  |
| Step 8^a^ | SEX Female: 1 Male:0(1) | .731 | .618 | 1.400 | 1 | .237 | 2.078 | .619 | 6.979 |
|  | Current Smoker Yes:1 No: 0(1) | -2.475 | 1.243 | 3.963 | 1 | .047 | .084 | .007 | .963 |
|  | History of Diabetes Yes:1 No:0(1) | -.552 | .660 | .697 | 1 | .404 | .576 | .158 | 2.102 |
|  | History of Stroke Yes:1 No:0(1) | 1.747 | 1.034 | 2.856 | 1 | .091 | 5.739 | .756 | 43.540 |
|  | History of Percutaneous Coronary Intervention Yes:1 No:0(1) | -1.221 | .912 | 1.790 | 1 | .181 | .295 | .049 | 1.763 |
|  | History of CABG Yes:1 No:0(1) | 1.172 | .966 | 1.471 | 1 | .225 | 3.229 | .486 | 21.460 |
|  | History of CKD Yes:1 No:0(1) | -.315 | .782 | .162 | 1 | .687 | .730 | .157 | 3.381 |
|  | Taking ACEi, ARNi, or ARB Yes:1 No:0(1) | -1.291 | .768 | 2.830 | 1 | .093 | .275 | .061 | 1.238 |
|  | Left ventricular ejection fraction <50% Yes:1 No:0(1) | 1.370 | .740 | 3.431 | 1 | .064 | 3.936 | .924 | 16.777 |
|  | Heart failure diagnosis (0=No HF, 1= History of HF, 2=New HF) |  |  | .957 | 2 | .620 |  |  |  |
|  | Heart failure diagnosis (0=No HF, 1= History of HF, 2=New HF)(1) | .554 | .859 | .416 | 1 | .519 | 1.740 | .323 | 9.370 |
|  | Heart failure diagnosis (0=No HF, 1= History of HF, 2=New HF)(2) | .714 | .755 | .895 | 1 | .344 | 2.043 | .465 | 8.971 |
|  | In patient diuretic use(1) | .613 | .700 | .767 | 1 | .381 | 1.846 | .468 | 7.282 |
|  | Hyponatremia present up to 7 days of hospitalization Yes:1 No:0(1) | 2.037 | 1.084 | 3.530 | 1 | .060 | 7.665 | .916 | 64.163 |
|  | Constant | -5.249 | 1.267 | 17.177 | 1 | <.001 | .005 |  |  |
| Step 9^a^ | SEX Female: 1 Male:0(1) | .736 | .618 | 1.422 | 1 | .233 | 2.089 | .622 | 7.008 |
|  | Current Smoker Yes:1 No: 0(1) | -2.418 | 1.226 | 3.890 | 1 | .049 | .089 | .008 | .985 |
|  | History of Diabetes Yes:1 No:0(1) | -.569 | .656 | .753 | 1 | .386 | .566 | .157 | 2.047 |
|  | History of Stroke Yes:1 No:0(1) | 1.667 | 1.011 | 2.718 | 1 | .099 | 5.298 | .730 | 38.452 |
|  | History of Percutaneous Coronary Intervention Yes:1 No:0(1) | -1.146 | .885 | 1.679 | 1 | .195 | .318 | .056 | 1.800 |
|  | History of CABG Yes:1 No:0(1) | 1.058 | .916 | 1.333 | 1 | .248 | 2.879 | .478 | 17.340 |
|  | Taking ACEi, ARNi, or ARB Yes:1 No:0(1) | -1.220 | .743 | 2.692 | 1 | .101 | .295 | .069 | 1.268 |
|  | Left ventricular ejection fraction <50% Yes:1 No:0(1) | 1.377 | .736 | 3.498 | 1 | .061 | 3.965 | .936 | 16.792 |
|  | Heart failure diagnosis (0=No HF, 1= History of HF, 2=New HF) |  |  | .931 | 2 | .628 |  |  |  |
|  | Heart failure diagnosis (0=No HF, 1= History of HF, 2=New HF)(1) | .464 | .832 | .312 | 1 | .577 | 1.591 | .312 | 8.120 |
|  | Heart failure diagnosis (0=No HF, 1= History of HF, 2=New HF)(2) | .721 | .754 | .914 | 1 | .339 | 2.056 | .469 | 9.003 |
|  | In patient diuretic use(1) | .622 | .695 | .801 | 1 | .371 | 1.863 | .477 | 7.276 |
|  | Hyponatremia present up to 7 days of hospitalization Yes:1 No:0(1) | 2.057 | 1.081 | 3.621 | 1 | .057 | 7.821 | .940 | 65.043 |
|  | Constant | -5.340 | 1.261 | 17.933 | 1 | <.001 | .005 |  |  |
| Step 10^a^ | SEX Female: 1 Male:0(1) | .824 | .609 | 1.832 | 1 | .176 | 2.279 | .691 | 7.516 |
|  | Current Smoker Yes:1 No: 0(1) | -2.362 | 1.186 | 3.965 | 1 | .046 | .094 | .009 | .964 |
|  | History of Diabetes Yes:1 No:0(1) | -.541 | .642 | .709 | 1 | .400 | .582 | .165 | 2.050 |
|  | History of Stroke Yes:1 No:0(1) | 1.688 | .998 | 2.859 | 1 | .091 | 5.406 | .764 | 38.231 |
|  | History of Percutaneous Coronary Intervention Yes:1 No:0(1) | -1.030 | .874 | 1.389 | 1 | .239 | .357 | .064 | 1.980 |
|  | History of CABG Yes:1 No:0(1) | 1.135 | .893 | 1.614 | 1 | .204 | 3.112 | .540 | 17.927 |
|  | Taking ACEi, ARNi, or ARB Yes:1 No:0(1) | -1.166 | .740 | 2.487 | 1 | .115 | .311 | .073 | 1.328 |
|  | Left ventricular ejection fraction <50% Yes:1 No:0(1) | 1.594 | .703 | 5.141 | 1 | .023 | 4.925 | 1.241 | 19.540 |
|  | In patient diuretic use(1) | .596 | .684 | .759 | 1 | .384 | 1.815 | .475 | 6.936 |
|  | Hyponatremia present up to 7 days of hospitalization Yes:1 No:0(1) | 2.097 | 1.080 | 3.770 | 1 | .052 | 8.145 | .980 | 67.669 |
|  | Constant | -5.272 | 1.252 | 17.739 | 1 | <.001 | .005 |  |  |
| Step 11^a^ | SEX Female: 1 Male:0(1) | .811 | .607 | 1.785 | 1 | .182 | 2.251 | .685 | 7.404 |
|  | Current Smoker Yes:1 No: 0(1) | -2.221 | 1.150 | 3.732 | 1 | .053 | .108 | .011 | 1.033 |
|  | History of Stroke Yes:1 No:0(1) | 1.598 | .981 | 2.651 | 1 | .103 | 4.943 | .722 | 33.841 |
|  | History of Percutaneous Coronary Intervention Yes:1 No:0(1) | -.975 | .834 | 1.366 | 1 | .242 | .377 | .073 | 1.935 |
|  | History of CABG Yes:1 No:0(1) | .964 | .834 | 1.337 | 1 | .248 | 2.623 | .512 | 13.443 |
|  | Taking ACEi, ARNi, or ARB Yes:1 No:0(1) | -1.253 | .736 | 2.897 | 1 | .089 | .286 | .067 | 1.209 |
|  | Left ventricular ejection fraction <50% Yes:1 No:0(1) | 1.553 | .702 | 4.888 | 1 | .027 | 4.726 | 1.193 | 18.724 |
|  | In patient diuretic use(1) | .552 | .682 | .657 | 1 | .418 | 1.737 | .457 | 6.608 |
|  | Hyponatremia present up to 7 days of hospitalization Yes:1 No:0(1) | 2.114 | 1.078 | 3.849 | 1 | .050 | 8.280 | 1.002 | 68.432 |
|  | Constant | -5.411 | 1.242 | 18.980 | 1 | <.001 | .004 |  |  |
| Step 12^a^ | SEX Female: 1 Male:0(1) | .839 | .605 | 1.925 | 1 | .165 | 2.315 | .707 | 7.576 |
|  | Current Smoker Yes:1 No: 0(1) | -2.168 | 1.127 | 3.702 | 1 | .054 | .114 | .013 | 1.041 |
|  | History of Stroke Yes:1 No:0(1) | 1.548 | .973 | 2.533 | 1 | .111 | 4.704 | .699 | 31.664 |
|  | History of Percutaneous Coronary Intervention Yes:1 No:0(1) | -.963 | .827 | 1.356 | 1 | .244 | .382 | .075 | 1.931 |
|  | History of CABG Yes:1 No:0(1) | 1.079 | .815 | 1.750 | 1 | .186 | 2.940 | .595 | 14.535 |
|  | Taking ACEi, ARNi, or ARB Yes:1 No:0(1) | -1.167 | .725 | 2.594 | 1 | .107 | .311 | .075 | 1.288 |
|  | Left ventricular ejection fraction <50% Yes:1 No:0(1) | 1.661 | .693 | 5.750 | 1 | .016 | 5.264 | 1.354 | 20.460 |
|  | Hyponatremia present up to 7 days of hospitalization Yes:1 No:0(1) | 2.134 | 1.080 | 3.907 | 1 | .048 | 8.452 | 1.018 | 70.166 |
|  | Constant | -5.194 | 1.197 | 18.829 | 1 | <.001 | .006 |  |  |
| Step 13^a^ | SEX Female: 1 Male:0(1) | .909 | .599 | 2.303 | 1 | .129 | 2.481 | .767 | 8.026 |
|  | Current Smoker Yes:1 No: 0(1) | -2.014 | 1.093 | 3.397 | 1 | .065 | .133 | .016 | 1.136 |
|  | History of Stroke Yes:1 No:0(1) | 1.171 | .908 | 1.665 | 1 | .197 | 3.226 | .545 | 19.107 |
|  | History of CABG Yes:1 No:0(1) | .589 | .697 | .716 | 1 | .397 | 1.803 | .460 | 7.063 |
|  | Taking ACEi, ARNi, or ARB Yes:1 No:0(1) | -1.157 | .724 | 2.559 | 1 | .110 | .314 | .076 | 1.298 |
|  | Left ventricular ejection fraction <50% Yes:1 No:0(1) | 1.500 | .667 | 5.064 | 1 | .024 | 4.483 | 1.214 | 16.559 |
|  | Hyponatremia present up to 7 days of hospitalization Yes:1 No:0(1) | 2.079 | 1.069 | 3.778 | 1 | .052 | 7.994 | .983 | 65.020 |
|  | Constant | -5.203 | 1.172 | 19.692 | 1 | <.001 | .006 |  |  |
| Step 14^a^ | SEX Female: 1 Male:0(1) | .844 | .589 | 2.051 | 1 | .152 | 2.326 | .733 | 7.382 |
|  | Current Smoker Yes:1 No: 0(1) | -2.118 | 1.084 | 3.817 | 1 | .051 | .120 | .014 | 1.007 |
|  | History of Stroke Yes:1 No:0(1) | 1.169 | .911 | 1.648 | 1 | .199 | 3.218 | .540 | 19.173 |
|  | Taking ACEi, ARNi, or ARB Yes:1 No:0(1) | -1.069 | .709 | 2.273 | 1 | .132 | .343 | .086 | 1.378 |
|  | Left ventricular ejection fraction <50% Yes:1 No:0(1) | 1.552 | .659 | 5.544 | 1 | .019 | 4.721 | 1.297 | 17.181 |
|  | Hyponatremia present up to 7 days of hospitalization Yes:1 No:0(1) | 2.117 | 1.067 | 3.938 | 1 | .047 | 8.304 | 1.026 | 67.190 |
|  | Constant | -5.122 | 1.163 | 19.387 | 1 | <.001 | .006 |  |  |
| Step 15^a^ | SEX Female: 1 Male:0(1) | .864 | .585 | 2.187 | 1 | .139 | 2.374 | .755 | 7.465 |
|  | Current Smoker Yes:1 No: 0(1) | -2.028 | 1.065 | 3.628 | 1 | .057 | .132 | .016 | 1.060 |
|  | Taking ACEi, ARNi, or ARB Yes:1 No:0(1) | -.975 | .692 | 1.983 | 1 | .159 | .377 | .097 | 1.465 |
|  | Left ventricular ejection fraction <50% Yes:1 No:0(1) | 1.421 | .638 | 4.958 | 1 | .026 | 4.141 | 1.186 | 14.462 |
|  | Hyponatremia present up to 7 days of hospitalization Yes:1 No:0(1) | 2.144 | 1.063 | 4.066 | 1 | .044 | 8.531 | 1.062 | 68.528 |
|  | Constant | -4.986 | 1.151 | 18.752 | 1 | <.001 | .007 |  |  |
| Step 16^a^ | Current Smoker Yes:1 No: 0(1) | -1.996 | 1.062 | 3.534 | 1 | .060 | .136 | .017 | 1.089 |
|  | Taking ACEi, ARNi, or ARB Yes:1 No:0(1) | -.913 | .685 | 1.779 | 1 | .182 | .401 | .105 | 1.535 |
|  | Left ventricular ejection fraction <50% Yes:1 No:0(1) | 1.269 | .622 | 4.165 | 1 | .041 | 3.556 | 1.051 | 12.026 |
|  | Hyponatremia present up to 7 days of hospitalization Yes:1 No:0(1) | 2.096 | 1.059 | 3.918 | 1 | .048 | 8.130 | 1.021 | 64.739 |
|  | Constant | -4.473 | 1.076 | 17.287 | 1 | <.001 | .011 |  |  |
| Step 17^a^ | Current Smoker Yes:1 No: 0(1) | -1.932 | 1.058 | 3.330 | 1 | .068 | .145 | .018 | 1.154 |
|  | Left ventricular ejection fraction <50% Yes:1 No:0(1) | 1.250 | .617 | 4.097 | 1 | .043 | 3.489 | 1.040 | 11.701 |
|  | Hyponatremia present up to 7 days of hospitalization Yes:1 No:0(1) | 2.116 | 1.057 | 4.012 | 1 | .045 | 8.301 | 1.046 | 65.850 |
|  | Constant | -4.744 | 1.066 | 19.805 | 1 | <.001 | .009 |  |  |
| a. Variable(s) entered on step 1: SEX Female: 1 Male:0, Age 1<65 2:≥ 65, Current Smoker Yes:1 No: 0, History of HTN Yes:1 No:0, History of Diabetes Yes:1 No:0, History of Stroke Yes:1 No:0, History of Transient Ischemic Attack Yes:1 No:0, History of Peripheral Vascular Disease Yes:1 No:0, History of Cancer Yes:1 No:0, History of Myocardial Infarction Yes:1 No:0, History of Percutaneous Coronary Intervention Yes:1 No:0, History of CABG Yes:1 No:0, History of CKD Yes:1 No:0, Taking ACEi, ARNi, or ARB Yes:1 No:0, Taking Diuretics Yes:1 No:0, Left ventricular ejection fraction <50% Yes:1 No:0, Heart failure diagnosis (0=No HF, 1= History of HF, 2=New HF), In patient diuretic use, Hyponatremia present up to 7 days of hospitalization Yes:1 No:0. | | | | | | | | | |

**Dependent variable: 30-day mortality**

**Independent variable: Hyponatremia up to 7 days of admission**

| **Omnibus Tests of Model Coefficients** | | | | |
| --- | --- | --- | --- | --- |
|  | | Chi-square | df | Sig. |
| Step 1 | Step | 36.290 | 20 | .014 |
|  | Block | 36.290 | 20 | .014 |
|  | Model | 36.290 | 20 | .014 |
| Step 2^a^ | Step | -.002 | 1 | .962 |
|  | Block | 36.288 | 19 | .010 |
|  | Model | 36.288 | 19 | .010 |
| Step 3^a^ | Step | -.026 | 1 | .871 |
|  | Block | 36.262 | 18 | .007 |
|  | Model | 36.262 | 18 | .007 |
| Step 4^a^ | Step | -.025 | 1 | .874 |
|  | Block | 36.236 | 17 | .004 |
|  | Model | 36.236 | 17 | .004 |
| Step 5^a^ | Step | -.028 | 1 | .867 |
|  | Block | 36.208 | 16 | .003 |
|  | Model | 36.208 | 16 | .003 |
| Step 6^a^ | Step | -.052 | 1 | .820 |
|  | Block | 36.156 | 15 | .002 |
|  | Model | 36.156 | 15 | .002 |
| Step 7^a^ | Step | -.473 | 2 | .789 |
|  | Block | 35.683 | 13 | <.001 |
|  | Model | 35.683 | 14 | .001 |
| Step 8^a^ | Step | -.161 | 1 | .688 |
|  | Block | 35.523 | 12 | <.001 |
|  | Model | 35.523 | 12 | <.001 |
| Step 9^a^ | Step | -.429 | 1 | .512 |
|  | Block | 35.093 | 11 | <.001 |
|  | Model | 35.093 | 11 | <.001 |
| Step 10^a^ | Step | -.350 | 1 | .554 |
|  | Block | 34.743 | 10 | <.001 |
|  | Model | 34.743 | 10 | <.001 |
| Step 11^a^ | Step | -1.204 | 1 | .272 |
|  | Block | 33.539 | 9 | <.001 |
|  | Model | 33.539 | 9 | <.001 |
| Step 12^a^ | Step | -1.659 | 1 | .198 |
|  | Block | 31.879 | 8 | <.001 |
|  | Model | 31.879 | 8 | <.001 |
| Step 13^a^ | Step | -2.430 | 1 | .119 |
|  | Block | 29.449 | 7 | <.001 |
|  | Model | 29.449 | 7 | <.001 |
| Step 14^a^ | Step | -2.535 | 1 | .111 |
|  | Block | 26.914 | 6 | <.001 |
|  | Model | 26.914 | 6 | <.001 |
| Step 15^a^ | Step | -2.643 | 1 | .104 |
|  | Block | 24.270 | 5 | <.001 |
|  | Model | 24.270 | 5 | <.001 |
| a. A negative Chi-squares value indicates that the Chi-squares value has decreased from the previous step. | | | | |

| **Hosmer and Lemeshow Test** | | | |
| --- | --- | --- | --- |
| Step | Chi-square | df | Sig. |
| 1 | 11.045 | 8 | .199 |
| 2 | 11.079 | 8 | .197 |
| 3 | 7.858 | 8 | .447 |
| 4 | 4.583 | 8 | .801 |
| 5 | 5.233 | 8 | .732 |
| 6 | 3.318 | 8 | .913 |
| 7 | 6.870 | 8 | .551 |
| 8 | 3.008 | 8 | .934 |
| 9 | 7.812 | 8 | .452 |
| 10 | 4.370 | 8 | .822 |
| 11 | 11.133 | 8 | .194 |
| 12 | 9.216 | 8 | .324 |
| 13 | 9.085 | 8 | .335 |
| 14 | 7.062 | 8 | .530 |
| 15 | 6.681 | 7 | .463 |

| **Contingency Table for Hosmer and Lemeshow Test** | | | | | | |
| --- | --- | --- | --- | --- | --- | --- |
|  | | 30 day Mortality Yes:1 No:0 = no | | 30 day Mortality Yes:1 No:0 = yes | | Total |
|  |  | Observed | Expected | Observed | Expected |  |
| Step 1 | 1 | 22 | 21.963 | 0 | .037 | 22 |
|  | 2 | 22 | 21.886 | 0 | .114 | 22 |
|  | 3 | 22 | 21.815 | 0 | .185 | 22 |
|  | 4 | 22 | 21.674 | 0 | .326 | 22 |
|  | 5 | 22 | 21.441 | 0 | .559 | 22 |
|  | 6 | 22 | 21.094 | 0 | .906 | 22 |
|  | 7 | 17 | 20.481 | 5 | 1.519 | 22 |
|  | 8 | 21 | 20.219 | 2 | 2.781 | 23 |
|  | 9 | 18 | 17.674 | 4 | 4.326 | 22 |
|  | 10 | 13 | 12.754 | 9 | 9.246 | 22 |
| Step 2 | 1 | 22 | 21.963 | 0 | .037 | 22 |
|  | 2 | 22 | 21.886 | 0 | .114 | 22 |
|  | 3 | 22 | 21.815 | 0 | .185 | 22 |
|  | 4 | 22 | 21.672 | 0 | .328 | 22 |
|  | 5 | 22 | 21.443 | 0 | .557 | 22 |
|  | 6 | 22 | 21.095 | 0 | .905 | 22 |
|  | 7 | 17 | 20.484 | 5 | 1.516 | 22 |
|  | 8 | 21 | 20.212 | 2 | 2.788 | 23 |
|  | 9 | 18 | 17.671 | 4 | 4.329 | 22 |
|  | 10 | 13 | 12.759 | 9 | 9.241 | 22 |
| Step 3 | 1 | 22 | 21.963 | 0 | .037 | 22 |
|  | 2 | 22 | 21.885 | 0 | .115 | 22 |
|  | 3 | 22 | 21.815 | 0 | .185 | 22 |
|  | 4 | 22 | 21.672 | 0 | .328 | 22 |
|  | 5 | 22 | 21.434 | 0 | .566 | 22 |
|  | 6 | 22 | 21.086 | 0 | .914 | 22 |
|  | 7 | 18 | 20.470 | 4 | 1.530 | 22 |
|  | 8 | 18 | 19.412 | 4 | 2.588 | 22 |
|  | 9 | 19 | 17.762 | 3 | 4.238 | 22 |
|  | 10 | 14 | 13.501 | 9 | 9.499 | 23 |
| Step 4 | 1 | 22 | 21.963 | 0 | .037 | 22 |
|  | 2 | 22 | 21.883 | 0 | .117 | 22 |
|  | 3 | 25 | 24.784 | 0 | .216 | 25 |
|  | 4 | 23 | 22.611 | 0 | .389 | 23 |
|  | 5 | 22 | 21.385 | 0 | .615 | 22 |
|  | 6 | 21 | 21.942 | 2 | 1.058 | 23 |
|  | 7 | 19 | 20.288 | 3 | 1.712 | 22 |
|  | 8 | 18 | 19.052 | 4 | 2.948 | 22 |
|  | 9 | 19 | 17.262 | 3 | 4.738 | 22 |
|  | 10 | 10 | 9.830 | 8 | 8.170 | 18 |
| Step 5 | 1 | 22 | 21.962 | 0 | .038 | 22 |
|  | 2 | 21 | 20.888 | 0 | .112 | 21 |
|  | 3 | 20 | 19.837 | 0 | .163 | 20 |
|  | 4 | 22 | 21.698 | 0 | .302 | 22 |
|  | 5 | 23 | 22.433 | 0 | .567 | 23 |
|  | 6 | 21 | 21.124 | 1 | .876 | 22 |
|  | 7 | 19 | 20.535 | 3 | 1.465 | 22 |
|  | 8 | 18 | 19.542 | 4 | 2.458 | 22 |
|  | 9 | 20 | 18.021 | 2 | 3.979 | 22 |
|  | 10 | 15 | 14.960 | 10 | 10.040 | 25 |
| Step 6 | 1 | 22 | 21.960 | 0 | .040 | 22 |
|  | 2 | 23 | 22.874 | 0 | .126 | 23 |
|  | 3 | 25 | 24.779 | 0 | .221 | 25 |
|  | 4 | 21 | 20.637 | 0 | .363 | 21 |
|  | 5 | 23 | 22.367 | 0 | .633 | 23 |
|  | 6 | 22 | 22.863 | 2 | 1.137 | 24 |
|  | 7 | 19 | 20.265 | 3 | 1.735 | 22 |
|  | 8 | 19 | 19.009 | 3 | 2.991 | 22 |
|  | 9 | 18 | 17.098 | 4 | 4.902 | 22 |
|  | 10 | 9 | 9.150 | 8 | 7.850 | 17 |
| Step 7 | 1 | 23 | 22.955 | 0 | .045 | 23 |
|  | 2 | 22 | 21.876 | 0 | .124 | 22 |
|  | 3 | 18 | 17.841 | 0 | .159 | 18 |
|  | 4 | 22 | 21.711 | 0 | .289 | 22 |
|  | 5 | 22 | 21.437 | 0 | .563 | 22 |
|  | 6 | 22 | 21.158 | 0 | .842 | 22 |
|  | 7 | 19 | 21.513 | 4 | 1.487 | 23 |
|  | 8 | 19 | 19.546 | 3 | 2.454 | 22 |
|  | 9 | 18 | 17.721 | 4 | 4.279 | 22 |
|  | 10 | 16 | 15.242 | 9 | 9.758 | 25 |
| Step 8 | 1 | 22 | 21.957 | 0 | .043 | 22 |
|  | 2 | 21 | 20.888 | 0 | .112 | 21 |
|  | 3 | 22 | 21.798 | 0 | .202 | 22 |
|  | 4 | 22 | 21.681 | 0 | .319 | 22 |
|  | 5 | 23 | 22.368 | 0 | .632 | 23 |
|  | 6 | 23 | 22.974 | 1 | 1.026 | 24 |
|  | 7 | 19 | 20.462 | 3 | 1.538 | 22 |
|  | 8 | 19 | 19.229 | 3 | 2.771 | 22 |
|  | 9 | 17 | 17.401 | 5 | 4.599 | 22 |
|  | 10 | 13 | 12.241 | 8 | 8.759 | 21 |
| Step 9 | 1 | 22 | 21.954 | 0 | .046 | 22 |
|  | 2 | 23 | 22.873 | 0 | .127 | 23 |
|  | 3 | 19 | 18.827 | 0 | .173 | 19 |
|  | 4 | 22 | 21.687 | 0 | .313 | 22 |
|  | 5 | 23 | 22.375 | 0 | .625 | 23 |
|  | 6 | 21 | 20.162 | 0 | .838 | 21 |
|  | 7 | 18 | 20.576 | 4 | 1.424 | 22 |
|  | 8 | 20 | 20.266 | 3 | 2.734 | 23 |
|  | 9 | 19 | 17.623 | 3 | 4.377 | 22 |
|  | 10 | 14 | 14.657 | 10 | 9.343 | 24 |
| Step 10 | 1 | 23 | 22.947 | 0 | .053 | 23 |
|  | 2 | 22 | 21.880 | 0 | .120 | 22 |
|  | 3 | 18 | 17.832 | 0 | .168 | 18 |
|  | 4 | 23 | 22.651 | 0 | .349 | 23 |
|  | 5 | 22 | 21.416 | 0 | .584 | 22 |
|  | 6 | 22 | 21.094 | 0 | .906 | 22 |
|  | 7 | 19 | 20.531 | 3 | 1.469 | 22 |
|  | 8 | 21 | 21.961 | 4 | 3.039 | 25 |
|  | 9 | 19 | 18.513 | 4 | 4.487 | 23 |
|  | 10 | 12 | 12.174 | 9 | 8.826 | 21 |
| Step 11 | 1 | 23 | 22.931 | 0 | .069 | 23 |
|  | 2 | 23 | 22.824 | 0 | .176 | 23 |
|  | 3 | 23 | 22.730 | 0 | .270 | 23 |
|  | 4 | 23 | 22.525 | 0 | .475 | 23 |
|  | 5 | 25 | 24.146 | 0 | .854 | 25 |
|  | 6 | 19 | 20.894 | 3 | 1.106 | 22 |
|  | 7 | 19 | 20.225 | 3 | 1.775 | 22 |
|  | 8 | 21 | 18.066 | 0 | 2.934 | 21 |
|  | 9 | 15 | 17.282 | 7 | 4.718 | 22 |
|  | 10 | 10 | 9.377 | 7 | 7.623 | 17 |
| Step 12 | 1 | 22 | 21.929 | 0 | .071 | 22 |
|  | 2 | 21 | 20.839 | 0 | .161 | 21 |
|  | 3 | 22 | 21.708 | 0 | .292 | 22 |
|  | 4 | 22 | 21.578 | 0 | .422 | 22 |
|  | 5 | 26 | 25.132 | 0 | .868 | 26 |
|  | 6 | 20 | 21.876 | 3 | 1.124 | 23 |
|  | 7 | 19 | 19.398 | 2 | 1.602 | 21 |
|  | 8 | 24 | 21.647 | 1 | 3.353 | 25 |
|  | 9 | 14 | 16.597 | 7 | 4.403 | 21 |
|  | 10 | 11 | 10.295 | 7 | 7.705 | 18 |
| Step 13 | 1 | 20 | 19.922 | 0 | .078 | 20 |
|  | 2 | 18 | 17.851 | 0 | .149 | 18 |
|  | 3 | 20 | 19.788 | 0 | .212 | 20 |
|  | 4 | 24 | 23.470 | 0 | .530 | 24 |
|  | 5 | 19 | 18.399 | 0 | .601 | 19 |
|  | 6 | 20 | 20.160 | 1 | .840 | 21 |
|  | 7 | 19 | 21.718 | 4 | 1.282 | 23 |
|  | 8 | 14 | 14.274 | 2 | 1.726 | 16 |
|  | 9 | 19 | 17.244 | 1 | 2.756 | 20 |
|  | 10 | 28 | 28.174 | 12 | 11.826 | 40 |
| Step 14 | 1 | 21 | 20.909 | 0 | .091 | 21 |
|  | 2 | 27 | 26.706 | 0 | .294 | 27 |
|  | 3 | 23 | 22.600 | 0 | .400 | 23 |
|  | 4 | 20 | 21.372 | 2 | .628 | 22 |
|  | 5 | 24 | 23.000 | 0 | 1.000 | 24 |
|  | 6 | 23 | 24.387 | 3 | 1.613 | 26 |
|  | 7 | 18 | 17.373 | 1 | 1.627 | 19 |
|  | 8 | 19 | 17.825 | 2 | 3.175 | 21 |
|  | 9 | 17 | 17.323 | 5 | 4.677 | 22 |
|  | 10 | 9 | 9.504 | 7 | 6.496 | 16 |
| Step 15 | 1 | 24 | 23.865 | 0 | .135 | 24 |
|  | 2 | 11 | 10.888 | 0 | .112 | 11 |
|  | 3 | 27 | 28.416 | 2 | .584 | 29 |
|  | 4 | 19 | 18.592 | 0 | .408 | 19 |
|  | 5 | 21 | 21.285 | 1 | .715 | 22 |
|  | 6 | 28 | 26.306 | 0 | 1.694 | 28 |
|  | 7 | 27 | 26.084 | 2 | 2.916 | 29 |
|  | 8 | 20 | 20.169 | 3 | 2.831 | 23 |
|  | 9 | 24 | 25.395 | 12 | 10.605 | 36 |

| **Variables in the Equation** | | | | | | | | | |
| --- | --- | --- | --- | --- | --- | --- | --- | --- | --- |
|  | | B | S.E. | Wald | df | Sig. | Exp(B) | 95% C.I.for EXP(B) | |
|  |  |  |  |  |  |  |  | Lower | Upper |
| Step 1^a^ | SEX Female: 1 Male:0(1) | .868 | .591 | 2.161 | 1 | .142 | 2.382 | .749 | 7.580 |
|  | Age 1<65 2:≥ 65(1) | .031 | .656 | .002 | 1 | .962 | 1.031 | .285 | 3.733 |
|  | Current Smoker Yes:1 No: 0(1) | -1.972 | 1.008 | 3.831 | 1 | .050 | .139 | .019 | 1.003 |
|  | History of HTN Yes:1 No:0(1) | -.150 | .812 | .034 | 1 | .854 | .861 | .175 | 4.230 |
|  | History of Diabetes Yes:1 No:0(1) | -1.102 | .668 | 2.721 | 1 | .099 | .332 | .090 | 1.231 |
|  | History of Stroke Yes:1 No:0(1) | 2.253 | .898 | 6.296 | 1 | .012 | 9.519 | 1.638 | 55.331 |
|  | History of Transient Ischemic Attack Yes:1 No:0(1) | -19.125 | 24648.228 | .000 | 1 | .999 | .000 | .000 | . |
|  | History of Peripheral Vascular Disease Yes:1 No:0(1) | .171 | 1.013 | .029 | 1 | .866 | 1.187 | .163 | 8.636 |
|  | History of Cancer Yes:1 No:0(1) | -.158 | .616 | .066 | 1 | .798 | .854 | .256 | 2.854 |
|  | History of Myocardial Infarction Yes:1 No:0(1) | .634 | .821 | .596 | 1 | .440 | 1.885 | .377 | 9.426 |
|  | History of Percutaneous Coronary Intervention Yes:1 No:0(1) | -1.081 | .914 | 1.400 | 1 | .237 | .339 | .057 | 2.033 |
|  | History of CABG Yes:1 No:0(1) | 1.359 | .893 | 2.316 | 1 | .128 | 3.891 | .676 | 22.387 |
|  | History of CKD Yes:1 No:0(1) | .735 | .697 | 1.112 | 1 | .292 | 2.085 | .532 | 8.174 |
|  | Taking ACEi, ARNi, or ARB Yes:1 No:0(1) | -1.053 | .707 | 2.220 | 1 | .136 | .349 | .087 | 1.394 |
|  | Taking Diuretics Yes:1 No:0(1) | .210 | .604 | .120 | 1 | .729 | 1.233 | .377 | 4.031 |
|  | Left ventricular ejection fraction <50% Yes:1 No:0(1) | 1.262 | .692 | 3.322 | 1 | .068 | 3.531 | .909 | 13.713 |
|  | Heart failure diagnosis (0=No HF, 1= History of HF, 2=New HF) |  |  | .532 | 2 | .766 |  |  |  |
|  | Heart failure diagnosis (0=No HF, 1= History of HF, 2=New HF)(1) | -.104 | .804 | .017 | 1 | .897 | .902 | .186 | 4.358 |
|  | Heart failure diagnosis (0=No HF, 1= History of HF, 2=New HF)(2) | .447 | .719 | .386 | 1 | .535 | 1.563 | .382 | 6.397 |
|  | In patient diuretic use(1) | .104 | .599 | .030 | 1 | .862 | 1.110 | .343 | 3.592 |
|  | Hyponatremia present up to 7 days of hospitalization Yes:1 No:0(1) | 1.840 | .832 | 4.893 | 1 | .027 | 6.300 | 1.233 | 32.177 |
|  | Constant | -4.512 | 1.150 | 15.389 | 1 | <.001 | .011 |  |  |
| Step 2^a^ | SEX Female: 1 Male:0(1) | .870 | .588 | 2.188 | 1 | .139 | 2.388 | .754 | 7.567 |
|  | Current Smoker Yes:1 No: 0(1) | -1.981 | .991 | 3.995 | 1 | .046 | .138 | .020 | .962 |
|  | History of HTN Yes:1 No:0(1) | -.146 | .807 | .033 | 1 | .857 | .864 | .178 | 4.207 |
|  | History of Diabetes Yes:1 No:0(1) | -1.102 | .668 | 2.717 | 1 | .099 | .332 | .090 | 1.232 |
|  | History of Stroke Yes:1 No:0(1) | 2.258 | .894 | 6.380 | 1 | .012 | 9.560 | 1.658 | 55.105 |
|  | History of Transient Ischemic Attack Yes:1 No:0(1) | -19.138 | 24638.793 | .000 | 1 | .999 | .000 | .000 | . |
|  | History of Peripheral Vascular Disease Yes:1 No:0(1) | .163 | .999 | .027 | 1 | .870 | 1.178 | .166 | 8.343 |
|  | History of Cancer Yes:1 No:0(1) | -.157 | .615 | .065 | 1 | .799 | .855 | .256 | 2.854 |
|  | History of Myocardial Infarction Yes:1 No:0(1) | .629 | .814 | .597 | 1 | .440 | 1.875 | .380 | 9.242 |
|  | History of Percutaneous Coronary Intervention Yes:1 No:0(1) | -1.080 | .913 | 1.398 | 1 | .237 | .340 | .057 | 2.034 |
|  | History of CABG Yes:1 No:0(1) | 1.362 | .890 | 2.344 | 1 | .126 | 3.905 | .683 | 22.336 |
|  | History of CKD Yes:1 No:0(1) | .743 | .676 | 1.210 | 1 | .271 | 2.102 | .559 | 7.901 |
|  | Taking ACEi, ARNi, or ARB Yes:1 No:0(1) | -1.050 | .703 | 2.228 | 1 | .135 | .350 | .088 | 1.389 |
|  | Taking Diuretics Yes:1 No:0(1) | .213 | .601 | .125 | 1 | .723 | 1.237 | .381 | 4.016 |
|  | Left ventricular ejection fraction <50% Yes:1 No:0(1) | 1.266 | .685 | 3.416 | 1 | .065 | 3.548 | .926 | 13.586 |
|  | Heart failure diagnosis (0=No HF, 1= History of HF, 2=New HF) |  |  | .536 | 2 | .765 |  |  |  |
|  | Heart failure diagnosis (0=No HF, 1= History of HF, 2=New HF)(1) | -.101 | .803 | .016 | 1 | .900 | .904 | .187 | 4.357 |
|  | Heart failure diagnosis (0=No HF, 1= History of HF, 2=New HF)(2) | .449 | .717 | .392 | 1 | .531 | 1.566 | .384 | 6.388 |
|  | In patient diuretic use(1) | .109 | .590 | .034 | 1 | .853 | 1.115 | .351 | 3.549 |
|  | Hyponatremia present up to 7 days of hospitalization Yes:1 No:0(1) | 1.838 | .830 | 4.908 | 1 | .027 | 6.282 | 1.236 | 31.929 |
|  | Constant | -4.507 | 1.145 | 15.493 | 1 | <.001 | .011 |  |  |
| Step 3^a^ | SEX Female: 1 Male:0(1) | .862 | .586 | 2.160 | 1 | .142 | 2.367 | .750 | 7.467 |
|  | Current Smoker Yes:1 No: 0(1) | -1.962 | .981 | 3.998 | 1 | .046 | .141 | .021 | .962 |
|  | History of HTN Yes:1 No:0(1) | -.128 | .800 | .025 | 1 | .873 | .880 | .184 | 4.219 |
|  | History of Diabetes Yes:1 No:0(1) | -1.075 | .646 | 2.766 | 1 | .096 | .341 | .096 | 1.211 |
|  | History of Stroke Yes:1 No:0(1) | 2.251 | .894 | 6.340 | 1 | .012 | 9.497 | 1.647 | 54.773 |
|  | History of Transient Ischemic Attack Yes:1 No:0(1) | -18.999 | 24648.307 | .000 | 1 | .999 | .000 | .000 | . |
|  | History of Cancer Yes:1 No:0(1) | -.140 | .606 | .053 | 1 | .818 | .870 | .265 | 2.854 |
|  | History of Myocardial Infarction Yes:1 No:0(1) | .630 | .813 | .600 | 1 | .438 | 1.878 | .381 | 9.250 |
|  | History of Percutaneous Coronary Intervention Yes:1 No:0(1) | -1.083 | .914 | 1.405 | 1 | .236 | .339 | .056 | 2.030 |
|  | History of CABG Yes:1 No:0(1) | 1.389 | .874 | 2.530 | 1 | .112 | 4.012 | .724 | 22.231 |
|  | History of CKD Yes:1 No:0(1) | .724 | .668 | 1.175 | 1 | .278 | 2.062 | .557 | 7.635 |
|  | Taking ACEi, ARNi, or ARB Yes:1 No:0(1) | -1.073 | .690 | 2.417 | 1 | .120 | .342 | .088 | 1.323 |
|  | Taking Diuretics Yes:1 No:0(1) | .227 | .595 | .146 | 1 | .703 | 1.255 | .391 | 4.031 |
|  | Left ventricular ejection fraction <50% Yes:1 No:0(1) | 1.274 | .685 | 3.457 | 1 | .063 | 3.575 | .933 | 13.690 |
|  | Heart failure diagnosis (0=No HF, 1= History of HF, 2=New HF) |  |  | .530 | 2 | .767 |  |  |  |
|  | Heart failure diagnosis (0=No HF, 1= History of HF, 2=New HF)(1) | -.098 | .805 | .015 | 1 | .903 | .907 | .187 | 4.396 |
|  | Heart failure diagnosis (0=No HF, 1= History of HF, 2=New HF)(2) | .448 | .717 | .391 | 1 | .532 | 1.566 | .384 | 6.381 |
|  | In patient diuretic use(1) | .104 | .589 | .031 | 1 | .860 | 1.109 | .350 | 3.519 |
|  | Hyponatremia present up to 7 days of hospitalization Yes:1 No:0(1) | 1.830 | .827 | 4.904 | 1 | .027 | 6.236 | 1.234 | 31.516 |
|  | Constant | -4.512 | 1.147 | 15.472 | 1 | <.001 | .011 |  |  |
| Step 4^a^ | SEX Female: 1 Male:0(1) | .877 | .578 | 2.305 | 1 | .129 | 2.405 | .775 | 7.465 |
|  | Current Smoker Yes:1 No: 0(1) | -1.943 | .972 | 3.990 | 1 | .046 | .143 | .021 | .964 |
|  | History of Diabetes Yes:1 No:0(1) | -1.083 | .645 | 2.819 | 1 | .093 | .339 | .096 | 1.199 |
|  | History of Stroke Yes:1 No:0(1) | 2.269 | .888 | 6.528 | 1 | .011 | 9.673 | 1.696 | 55.159 |
|  | History of Transient Ischemic Attack Yes:1 No:0(1) | -18.992 | 24650.123 | .000 | 1 | .999 | .000 | .000 | . |
|  | History of Cancer Yes:1 No:0(1) | -.137 | .606 | .051 | 1 | .822 | .872 | .266 | 2.863 |
|  | History of Myocardial Infarction Yes:1 No:0(1) | .643 | .813 | .626 | 1 | .429 | 1.902 | .387 | 9.352 |
|  | History of Percutaneous Coronary Intervention Yes:1 No:0(1) | -1.111 | .899 | 1.528 | 1 | .216 | .329 | .057 | 1.917 |
|  | History of CABG Yes:1 No:0(1) | 1.382 | .873 | 2.506 | 1 | .113 | 3.983 | .719 | 22.046 |
|  | History of CKD Yes:1 No:0(1) | .690 | .633 | 1.187 | 1 | .276 | 1.993 | .576 | 6.892 |
|  | Taking ACEi, ARNi, or ARB Yes:1 No:0(1) | -1.094 | .677 | 2.614 | 1 | .106 | .335 | .089 | 1.261 |
|  | Taking Diuretics Yes:1 No:0(1) | .211 | .586 | .130 | 1 | .719 | 1.235 | .392 | 3.892 |
|  | Left ventricular ejection fraction <50% Yes:1 No:0(1) | 1.300 | .666 | 3.814 | 1 | .051 | 3.671 | .995 | 13.540 |
|  | Heart failure diagnosis (0=No HF, 1= History of HF, 2=New HF) |  |  | .506 | 2 | .776 |  |  |  |
|  | Heart failure diagnosis (0=No HF, 1= History of HF, 2=New HF)(1) | -.079 | .798 | .010 | 1 | .921 | .924 | .193 | 4.415 |
|  | Heart failure diagnosis (0=No HF, 1= History of HF, 2=New HF)(2) | .433 | .709 | .373 | 1 | .542 | 1.542 | .384 | 6.187 |
|  | In patient diuretic use(1) | .098 | .588 | .028 | 1 | .867 | 1.103 | .348 | 3.492 |
|  | Hyponatremia present up to 7 days of hospitalization Yes:1 No:0(1) | 1.821 | .824 | 4.885 | 1 | .027 | 6.179 | 1.229 | 31.068 |
|  | Constant | -4.600 | 1.009 | 20.776 | 1 | <.001 | .010 |  |  |
| Step 5^a^ | SEX Female: 1 Male:0(1) | .885 | .577 | 2.353 | 1 | .125 | 2.422 | .782 | 7.499 |
|  | Current Smoker Yes:1 No: 0(1) | -1.927 | .963 | 4.006 | 1 | .045 | .146 | .022 | .961 |
|  | History of Diabetes Yes:1 No:0(1) | -1.080 | .645 | 2.800 | 1 | .094 | .340 | .096 | 1.203 |
|  | History of Stroke Yes:1 No:0(1) | 2.270 | .886 | 6.569 | 1 | .010 | 9.683 | 1.706 | 54.955 |
|  | History of Transient Ischemic Attack Yes:1 No:0(1) | -19.044 | 24637.853 | .000 | 1 | .999 | .000 | .000 | . |
|  | History of Cancer Yes:1 No:0(1) | -.138 | .606 | .052 | 1 | .820 | .871 | .265 | 2.860 |
|  | History of Myocardial Infarction Yes:1 No:0(1) | .640 | .810 | .625 | 1 | .429 | 1.896 | .388 | 9.271 |
|  | History of Percutaneous Coronary Intervention Yes:1 No:0(1) | -1.106 | .896 | 1.524 | 1 | .217 | .331 | .057 | 1.915 |
|  | History of CABG Yes:1 No:0(1) | 1.397 | .869 | 2.585 | 1 | .108 | 4.042 | .736 | 22.183 |
|  | History of CKD Yes:1 No:0(1) | .688 | .633 | 1.183 | 1 | .277 | 1.990 | .576 | 6.873 |
|  | Taking ACEi, ARNi, or ARB Yes:1 No:0(1) | -1.083 | .674 | 2.585 | 1 | .108 | .338 | .090 | 1.268 |
|  | Taking Diuretics Yes:1 No:0(1) | .216 | .586 | .136 | 1 | .712 | 1.241 | .394 | 3.912 |
|  | Left ventricular ejection fraction <50% Yes:1 No:0(1) | 1.322 | .654 | 4.083 | 1 | .043 | 3.753 | 1.041 | 13.533 |
|  | Heart failure diagnosis (0=No HF, 1= History of HF, 2=New HF) |  |  | .487 | 2 | .784 |  |  |  |
|  | Heart failure diagnosis (0=No HF, 1= History of HF, 2=New HF)(1) | -.068 | .795 | .007 | 1 | .932 | .935 | .197 | 4.440 |
|  | Heart failure diagnosis (0=No HF, 1= History of HF, 2=New HF)(2) | .428 | .710 | .363 | 1 | .547 | 1.534 | .381 | 6.165 |
|  | Hyponatremia present up to 7 days of hospitalization Yes:1 No:0(1) | 1.823 | .824 | 4.891 | 1 | .027 | 6.188 | 1.230 | 31.126 |
|  | Constant | -4.570 | .990 | 21.308 | 1 | <.001 | .010 |  |  |
| Step 6^a^ | SEX Female: 1 Male:0(1) | .867 | .571 | 2.303 | 1 | .129 | 2.380 | .777 | 7.291 |
|  | Current Smoker Yes:1 No: 0(1) | -1.872 | .929 | 4.062 | 1 | .044 | .154 | .025 | .950 |
|  | History of Diabetes Yes:1 No:0(1) | -1.056 | .637 | 2.750 | 1 | .097 | .348 | .100 | 1.212 |
|  | History of Stroke Yes:1 No:0(1) | 2.258 | .883 | 6.544 | 1 | .011 | 9.566 | 1.696 | 53.966 |
|  | History of Transient Ischemic Attack Yes:1 No:0(1) | -19.009 | 24679.252 | .000 | 1 | .999 | .000 | .000 | . |
|  | History of Myocardial Infarction Yes:1 No:0(1) | .621 | .804 | .597 | 1 | .440 | 1.861 | .385 | 8.989 |
|  | History of Percutaneous Coronary Intervention Yes:1 No:0(1) | -1.100 | .892 | 1.520 | 1 | .218 | .333 | .058 | 1.913 |
|  | History of CABG Yes:1 No:0(1) | 1.393 | .867 | 2.582 | 1 | .108 | 4.028 | .736 | 22.039 |
|  | History of CKD Yes:1 No:0(1) | .695 | .634 | 1.202 | 1 | .273 | 2.003 | .579 | 6.937 |
|  | Taking ACEi, ARNi, or ARB Yes:1 No:0(1) | -1.063 | .667 | 2.542 | 1 | .111 | .345 | .093 | 1.276 |
|  | Taking Diuretics Yes:1 No:0(1) | .223 | .585 | .145 | 1 | .703 | 1.250 | .397 | 3.933 |
|  | Left ventricular ejection fraction <50% Yes:1 No:0(1) | 1.317 | .655 | 4.040 | 1 | .044 | 3.731 | 1.033 | 13.474 |
|  | Heart failure diagnosis (0=No HF, 1= History of HF, 2=New HF) |  |  | .481 | 2 | .786 |  |  |  |
|  | Heart failure diagnosis (0=No HF, 1= History of HF, 2=New HF)(1) | -.065 | .795 | .007 | 1 | .934 | .937 | .197 | 4.453 |
|  | Heart failure diagnosis (0=No HF, 1= History of HF, 2=New HF)(2) | .426 | .711 | .360 | 1 | .549 | 1.532 | .380 | 6.171 |
|  | Hyponatremia present up to 7 days of hospitalization Yes:1 No:0(1) | 1.801 | .818 | 4.840 | 1 | .028 | 6.053 | 1.217 | 30.102 |
|  | Constant | -4.601 | .982 | 21.949 | 1 | <.001 | .010 |  |  |
| Step 7^a^ | SEX Female: 1 Male:0(1) | .925 | .566 | 2.668 | 1 | .102 | 2.522 | .831 | 7.655 |
|  | Current Smoker Yes:1 No: 0(1) | -1.864 | .918 | 4.120 | 1 | .042 | .155 | .026 | .938 |
|  | History of Diabetes Yes:1 No:0(1) | -1.072 | .634 | 2.859 | 1 | .091 | .342 | .099 | 1.186 |
|  | History of Stroke Yes:1 No:0(1) | 2.256 | .876 | 6.628 | 1 | .010 | 9.547 | 1.714 | 53.191 |
|  | History of Transient Ischemic Attack Yes:1 No:0(1) | -19.083 | 24655.651 | .000 | 1 | .999 | .000 | .000 | . |
|  | History of Myocardial Infarction Yes:1 No:0(1) | .515 | .793 | .423 | 1 | .516 | 1.674 | .354 | 7.919 |
|  | History of Percutaneous Coronary Intervention Yes:1 No:0(1) | -.939 | .855 | 1.205 | 1 | .272 | .391 | .073 | 2.090 |
|  | History of CABG Yes:1 No:0(1) | 1.349 | .843 | 2.561 | 1 | .110 | 3.853 | .738 | 20.105 |
|  | History of CKD Yes:1 No:0(1) | .648 | .615 | 1.111 | 1 | .292 | 1.912 | .573 | 6.379 |
|  | Taking ACEi, ARNi, or ARB Yes:1 No:0(1) | -1.086 | .662 | 2.688 | 1 | .101 | .338 | .092 | 1.236 |
|  | Taking Diuretics Yes:1 No:0(1) | .229 | .568 | .162 | 1 | .687 | 1.257 | .413 | 3.829 |
|  | Left ventricular ejection fraction <50% Yes:1 No:0(1) | 1.454 | .613 | 5.633 | 1 | .018 | 4.280 | 1.288 | 14.222 |
|  | Hyponatremia present up to 7 days of hospitalization Yes:1 No:0(1) | 1.803 | .816 | 4.880 | 1 | .027 | 6.066 | 1.225 | 30.027 |
|  | Constant | -4.590 | .980 | 21.951 | 1 | <.001 | .010 |  |  |
| Step 8^a^ | SEX Female: 1 Male:0(1) | .941 | .566 | 2.759 | 1 | .097 | 2.562 | .844 | 7.776 |
|  | Current Smoker Yes:1 No: 0(1) | -1.879 | .914 | 4.231 | 1 | .040 | .153 | .025 | .915 |
|  | History of Diabetes Yes:1 No:0(1) | -1.058 | .634 | 2.785 | 1 | .095 | .347 | .100 | 1.203 |
|  | History of Stroke Yes:1 No:0(1) | 2.264 | .873 | 6.723 | 1 | .010 | 9.624 | 1.738 | 53.296 |
|  | History of Transient Ischemic Attack Yes:1 No:0(1) | -18.948 | 24639.328 | .000 | 1 | .999 | .000 | .000 | . |
|  | History of Myocardial Infarction Yes:1 No:0(1) | .539 | .796 | .460 | 1 | .498 | 1.715 | .361 | 8.156 |
|  | History of Percutaneous Coronary Intervention Yes:1 No:0(1) | -.964 | .851 | 1.283 | 1 | .257 | .381 | .072 | 2.021 |
|  | History of CABG Yes:1 No:0(1) | 1.373 | .845 | 2.641 | 1 | .104 | 3.947 | .754 | 20.668 |
|  | History of CKD Yes:1 No:0(1) | .662 | .613 | 1.166 | 1 | .280 | 1.938 | .583 | 6.440 |
|  | Taking ACEi, ARNi, or ARB Yes:1 No:0(1) | -1.067 | .662 | 2.597 | 1 | .107 | .344 | .094 | 1.259 |
|  | Left ventricular ejection fraction <50% Yes:1 No:0(1) | 1.440 | .611 | 5.557 | 1 | .018 | 4.219 | 1.275 | 13.967 |
|  | Hyponatremia present up to 7 days of hospitalization Yes:1 No:0(1) | 1.804 | .816 | 4.884 | 1 | .027 | 6.072 | 1.226 | 30.061 |
|  | Constant | -4.530 | .962 | 22.159 | 1 | <.001 | .011 |  |  |
| Step 9^a^ | SEX Female: 1 Male:0(1) | .982 | .563 | 3.047 | 1 | .081 | 2.670 | .886 | 8.044 |
|  | Current Smoker Yes:1 No: 0(1) | -1.864 | .912 | 4.184 | 1 | .041 | .155 | .026 | .925 |
|  | History of Diabetes Yes:1 No:0(1) | -1.101 | .633 | 3.020 | 1 | .082 | .333 | .096 | 1.151 |
|  | History of Stroke Yes:1 No:0(1) | 2.201 | .870 | 6.404 | 1 | .011 | 9.034 | 1.643 | 49.683 |
|  | History of Myocardial Infarction Yes:1 No:0(1) | .472 | .792 | .355 | 1 | .551 | 1.604 | .339 | 7.579 |
|  | History of Percutaneous Coronary Intervention Yes:1 No:0(1) | -1.026 | .856 | 1.438 | 1 | .230 | .358 | .067 | 1.918 |
|  | History of CABG Yes:1 No:0(1) | 1.485 | .826 | 3.231 | 1 | .072 | 4.415 | .874 | 22.299 |
|  | History of CKD Yes:1 No:0(1) | .629 | .614 | 1.049 | 1 | .306 | 1.876 | .563 | 6.254 |
|  | Taking ACEi, ARNi, or ARB Yes:1 No:0(1) | -1.046 | .662 | 2.502 | 1 | .114 | .351 | .096 | 1.284 |
|  | Left ventricular ejection fraction <50% Yes:1 No:0(1) | 1.474 | .610 | 5.831 | 1 | .016 | 4.367 | 1.320 | 14.450 |
|  | Hyponatremia present up to 7 days of hospitalization Yes:1 No:0(1) | 1.781 | .815 | 4.771 | 1 | .029 | 5.933 | 1.201 | 29.320 |
|  | Constant | -4.534 | .966 | 22.051 | 1 | <.001 | .011 |  |  |
| Step 10^a^ | SEX Female: 1 Male:0(1) | .968 | .561 | 2.976 | 1 | .085 | 2.632 | .877 | 7.903 |
|  | Current Smoker Yes:1 No: 0(1) | -1.820 | .905 | 4.042 | 1 | .044 | .162 | .028 | .955 |
|  | History of Diabetes Yes:1 No:0(1) | -1.059 | .623 | 2.887 | 1 | .089 | .347 | .102 | 1.177 |
|  | History of Stroke Yes:1 No:0(1) | 2.090 | .839 | 6.210 | 1 | .013 | 8.081 | 1.562 | 41.802 |
|  | History of Percutaneous Coronary Intervention Yes:1 No:0(1) | -.823 | .778 | 1.118 | 1 | .290 | .439 | .095 | 2.019 |
|  | History of CABG Yes:1 No:0(1) | 1.533 | .823 | 3.474 | 1 | .062 | 4.634 | .924 | 23.242 |
|  | History of CKD Yes:1 No:0(1) | .688 | .607 | 1.284 | 1 | .257 | 1.990 | .605 | 6.542 |
|  | Taking ACEi, ARNi, or ARB Yes:1 No:0(1) | -1.035 | .665 | 2.422 | 1 | .120 | .355 | .096 | 1.308 |
|  | Left ventricular ejection fraction <50% Yes:1 No:0(1) | 1.448 | .603 | 5.773 | 1 | .016 | 4.255 | 1.306 | 13.864 |
|  | Hyponatremia present up to 7 days of hospitalization Yes:1 No:0(1) | 1.740 | .812 | 4.594 | 1 | .032 | 5.699 | 1.160 | 27.992 |
|  | Constant | -4.469 | .955 | 21.891 | 1 | <.001 | .011 |  |  |
| Step 11^a^ | SEX Female: 1 Male:0(1) | 1.017 | .559 | 3.308 | 1 | .069 | 2.764 | .924 | 8.265 |
|  | Current Smoker Yes:1 No: 0(1) | -1.686 | .871 | 3.752 | 1 | .053 | .185 | .034 | 1.020 |
|  | History of Diabetes Yes:1 No:0(1) | -1.027 | .608 | 2.856 | 1 | .091 | .358 | .109 | 1.178 |
|  | History of Stroke Yes:1 No:0(1) | 1.780 | .777 | 5.246 | 1 | .022 | 5.932 | 1.293 | 27.216 |
|  | History of CABG Yes:1 No:0(1) | 1.086 | .682 | 2.539 | 1 | .111 | 2.964 | .779 | 11.278 |
|  | History of CKD Yes:1 No:0(1) | .776 | .593 | 1.714 | 1 | .190 | 2.174 | .680 | 6.951 |
|  | Taking ACEi, ARNi, or ARB Yes:1 No:0(1) | -1.079 | .665 | 2.633 | 1 | .105 | .340 | .092 | 1.251 |
|  | Left ventricular ejection fraction <50% Yes:1 No:0(1) | 1.333 | .585 | 5.191 | 1 | .023 | 3.793 | 1.205 | 11.940 |
|  | Hyponatremia present up to 7 days of hospitalization Yes:1 No:0(1) | 1.676 | .801 | 4.382 | 1 | .036 | 5.345 | 1.113 | 25.678 |
|  | Constant | -4.491 | .946 | 22.531 | 1 | <.001 | .011 |  |  |
| Step 12^a^ | SEX Female: 1 Male:0(1) | 1.019 | .553 | 3.399 | 1 | .065 | 2.770 | .938 | 8.183 |
|  | Current Smoker Yes:1 No: 0(1) | -1.792 | .864 | 4.303 | 1 | .038 | .167 | .031 | .906 |
|  | History of Diabetes Yes:1 No:0(1) | -.886 | .587 | 2.282 | 1 | .131 | .412 | .131 | 1.302 |
|  | History of Stroke Yes:1 No:0(1) | 1.889 | .764 | 6.110 | 1 | .013 | 6.612 | 1.479 | 29.565 |
|  | History of CABG Yes:1 No:0(1) | 1.251 | .658 | 3.618 | 1 | .057 | 3.493 | .963 | 12.674 |
|  | Taking ACEi, ARNi, or ARB Yes:1 No:0(1) | -1.189 | .663 | 3.219 | 1 | .073 | .305 | .083 | 1.116 |
|  | Left ventricular ejection fraction <50% Yes:1 No:0(1) | 1.262 | .579 | 4.749 | 1 | .029 | 3.532 | 1.135 | 10.987 |
|  | Hyponatremia present up to 7 days of hospitalization Yes:1 No:0(1) | 1.663 | .800 | 4.316 | 1 | .038 | 5.274 | 1.099 | 25.322 |
|  | Constant | -4.272 | .904 | 22.333 | 1 | <.001 | .014 |  |  |
| Step 13^a^ | SEX Female: 1 Male:0(1) | .990 | .545 | 3.301 | 1 | .069 | 2.691 | .925 | 7.826 |
|  | Current Smoker Yes:1 No: 0(1) | -1.565 | .828 | 3.573 | 1 | .059 | .209 | .041 | 1.060 |
|  | History of Stroke Yes:1 No:0(1) | 1.761 | .739 | 5.685 | 1 | .017 | 5.817 | 1.368 | 24.738 |
|  | History of CABG Yes:1 No:0(1) | 1.003 | .615 | 2.664 | 1 | .103 | 2.727 | .818 | 9.094 |
|  | Taking ACEi, ARNi, or ARB Yes:1 No:0(1) | -1.304 | .655 | 3.962 | 1 | .047 | .271 | .075 | .980 |
|  | Left ventricular ejection fraction <50% Yes:1 No:0(1) | 1.214 | .572 | 4.504 | 1 | .034 | 3.365 | 1.097 | 10.321 |
|  | Hyponatremia present up to 7 days of hospitalization Yes:1 No:0(1) | 1.714 | .796 | 4.636 | 1 | .031 | 5.550 | 1.166 | 26.410 |
|  | Constant | -4.565 | .899 | 25.801 | 1 | <.001 | .010 |  |  |
| Step 14^a^ | SEX Female: 1 Male:0(1) | .847 | .525 | 2.601 | 1 | .107 | 2.333 | .833 | 6.531 |
|  | Current Smoker Yes:1 No: 0(1) | -1.708 | .820 | 4.339 | 1 | .037 | .181 | .036 | .904 |
|  | History of Stroke Yes:1 No:0(1) | 1.795 | .738 | 5.912 | 1 | .015 | 6.018 | 1.416 | 25.576 |
|  | Taking ACEi, ARNi, or ARB Yes:1 No:0(1) | -1.157 | .633 | 3.338 | 1 | .068 | .315 | .091 | 1.088 |
|  | Left ventricular ejection fraction <50% Yes:1 No:0(1) | 1.279 | .563 | 5.154 | 1 | .023 | 3.593 | 1.191 | 10.838 |
|  | Hyponatremia present up to 7 days of hospitalization Yes:1 No:0(1) | 1.781 | .791 | 5.065 | 1 | .024 | 5.935 | 1.259 | 27.992 |
|  | Constant | -4.389 | .880 | 24.903 | 1 | <.001 | .012 |  |  |
| Step 15^a^ | Current Smoker Yes:1 No: 0(1) | -1.642 | .804 | 4.170 | 1 | .041 | .193 | .040 | .936 |
|  | History of Stroke Yes:1 No:0(1) | 1.885 | .743 | 6.435 | 1 | .011 | 6.586 | 1.535 | 28.257 |
|  | Taking ACEi, ARNi, or ARB Yes:1 No:0(1) | -1.071 | .620 | 2.984 | 1 | .084 | .343 | .102 | 1.155 |
|  | Left ventricular ejection fraction <50% Yes:1 No:0(1) | 1.158 | .556 | 4.338 | 1 | .037 | 3.184 | 1.071 | 9.467 |
|  | Hyponatremia present up to 7 days of hospitalization Yes:1 No:0(1) | 1.693 | .782 | 4.690 | 1 | .030 | 5.438 | 1.174 | 25.182 |
|  | Constant | -3.885 | .798 | 23.720 | 1 | <.001 | .021 |  |  |
| a. Variable(s) entered on step 1: SEX Female: 1 Male:0, Age 1<65 2:≥ 65, Current Smoker Yes:1 No: 0, History of HTN Yes:1 No:0, History of Diabetes Yes:1 No:0, History of Stroke Yes:1 No:0, History of Transient Ischemic Attack Yes:1 No:0, History of Peripheral Vascular Disease Yes:1 No:0, History of Cancer Yes:1 No:0, History of Myocardial Infarction Yes:1 No:0, History of Percutaneous Coronary Intervention Yes:1 No:0, History of CABG Yes:1 No:0, History of CKD Yes:1 No:0, Taking ACEi, ARNi, or ARB Yes:1 No:0, Taking Diuretics Yes:1 No:0, Left ventricular ejection fraction <50% Yes:1 No:0, Heart failure diagnosis (0=No HF, 1= History of HF, 2=New HF), In patient diuretic use, Hyponatremia present up to 7 days of hospitalization Yes:1 No:0. | | | | | | | | | |

**Dependent variable: ICU or CCU admission**

**Independent variable: Hyponatremia up to 7 days of admission**

| **Omnibus Tests of Model Coefficients** | | | | |
| --- | --- | --- | --- | --- |
|  | | Chi-square | df | Sig. |
| Step 1 | Step | 36.266 | 19 | .010 |
|  | Block | 36.266 | 19 | .010 |
|  | Model | 36.266 | 19 | .010 |
| Step 2^a^ | Step | -.080 | 1 | .777 |
|  | Block | 36.186 | 18 | .007 |
|  | Model | 36.186 | 18 | .007 |
| Step 3^a^ | Step | -.208 | 1 | .648 |
|  | Block | 35.978 | 17 | .005 |
|  | Model | 35.978 | 17 | .005 |
| Step 4^a^ | Step | -.547 | 1 | .460 |
|  | Block | 35.431 | 16 | .003 |
|  | Model | 35.431 | 16 | .003 |
| Step 5^a^ | Step | -.813 | 1 | .367 |
|  | Block | 34.618 | 15 | .003 |
|  | Model | 34.618 | 15 | .003 |
| Step 6^a^ | Step | -.644 | 1 | .422 |
|  | Block | 33.974 | 14 | .002 |
|  | Model | 33.974 | 14 | .002 |
| Step 7^a^ | Step | -1.041 | 1 | .308 |
|  | Block | 32.933 | 13 | .002 |
|  | Model | 32.933 | 13 | .002 |
| Step 8^a^ | Step | -.695 | 1 | .404 |
|  | Block | 32.238 | 12 | .001 |
|  | Model | 32.238 | 12 | .001 |
| Step 9^a^ | Step | -1.150 | 1 | .284 |
|  | Block | 31.088 | 11 | .001 |
|  | Model | 31.088 | 11 | .001 |
| Step 10^a^ | Step | -1.332 | 1 | .248 |
|  | Block | 29.757 | 10 | <.001 |
|  | Model | 29.757 | 10 | <.001 |
| Step 11^a^ | Step | -1.370 | 1 | .242 |
|  | Block | 28.386 | 9 | <.001 |
|  | Model | 28.386 | 9 | <.001 |
| Step 12^a^ | Step | -1.692 | 1 | .193 |
|  | Block | 26.695 | 8 | <.001 |
|  | Model | 26.695 | 8 | <.001 |
| Step 13^a^ | Step | -1.691 | 1 | .193 |
|  | Block | 25.003 | 7 | <.001 |
|  | Model | 25.003 | 7 | <.001 |
| Step 14^a^ | Step | -2.176 | 1 | .140 |
|  | Block | 22.828 | 6 | <.001 |
|  | Model | 22.828 | 6 | <.001 |
| Step 15^a^ | Step | -3.767 | 2 | .152 |
|  | Block | 19.061 | 4 | <.001 |
|  | Model | 19.061 | 5 | .002 |
| Step 16^a^ | Step | -1.766 | 1 | .184 |
|  | Block | 17.294 | 3 | <.001 |
|  | Model | 17.294 | 3 | <.001 |
| a. A negative Chi-squares value indicates that the Chi-squares value has decreased from the previous step. | | | | |

| **Hosmer and Lemeshow Test** | | | |
| --- | --- | --- | --- |
| Step | Chi-square | df | Sig. |
| 1 | 7.026 | 8 | .534 |
| 2 | 6.453 | 8 | .597 |
| 3 | 8.154 | 8 | .419 |
| 4 | 7.185 | 8 | .517 |
| 5 | 7.150 | 8 | .521 |
| 6 | 3.735 | 8 | .880 |
| 7 | 7.766 | 8 | .457 |
| 8 | 3.820 | 8 | .873 |
| 9 | 3.951 | 8 | .861 |
| 10 | 1.754 | 8 | .988 |
| 11 | 16.164 | 8 | .040 |
| 12 | 7.726 | 8 | .461 |
| 13 | 5.206 | 8 | .735 |
| 14 | 7.224 | 8 | .513 |
| 15 | 2.264 | 6 | .894 |
| 16 | 2.324 | 5 | .803 |

| **Classification Table**^a^ | | | | | |
| --- | --- | --- | --- | --- | --- |
|  | Observed | | Predicted | | |
|  |  |  | ICU or CCU Yes:1 No:0 | | Percentage Correct |
|  |  |  | no | yes |  |
| Step 1 | ICU or CCU Yes:1 No:0 | no | 35 | 50 | 41.2 |
|  |  | yes | 19 | 130 | 87.2 |
|  | Overall Percentage | |  |  | 70.5 |
| Step 2 | ICU or CCU Yes:1 No:0 | no | 36 | 49 | 42.4 |
|  |  | yes | 18 | 131 | 87.9 |
|  | Overall Percentage | |  |  | 71.4 |
| Step 3 | ICU or CCU Yes:1 No:0 | no | 35 | 50 | 41.2 |
|  |  | yes | 19 | 130 | 87.2 |
|  | Overall Percentage | |  |  | 70.5 |
| Step 4 | ICU or CCU Yes:1 No:0 | no | 35 | 50 | 41.2 |
|  |  | yes | 20 | 129 | 86.6 |
|  | Overall Percentage | |  |  | 70.1 |
| Step 5 | ICU or CCU Yes:1 No:0 | no | 34 | 51 | 40.0 |
|  |  | yes | 17 | 132 | 88.6 |
|  | Overall Percentage | |  |  | 70.9 |
| Step 6 | ICU or CCU Yes:1 No:0 | no | 35 | 50 | 41.2 |
|  |  | yes | 16 | 133 | 89.3 |
|  | Overall Percentage | |  |  | 71.8 |
| Step 7 | ICU or CCU Yes:1 No:0 | no | 35 | 50 | 41.2 |
|  |  | yes | 22 | 127 | 85.2 |
|  | Overall Percentage | |  |  | 69.2 |
| Step 8 | ICU or CCU Yes:1 No:0 | no | 33 | 52 | 38.8 |
|  |  | yes | 22 | 127 | 85.2 |
|  | Overall Percentage | |  |  | 68.4 |
| Step 9 | ICU or CCU Yes:1 No:0 | no | 34 | 51 | 40.0 |
|  |  | yes | 19 | 130 | 87.2 |
|  | Overall Percentage | |  |  | 70.1 |
| Step 10 | ICU or CCU Yes:1 No:0 | no | 33 | 52 | 38.8 |
|  |  | yes | 17 | 132 | 88.6 |
|  | Overall Percentage | |  |  | 70.5 |
| Step 11 | ICU or CCU Yes:1 No:0 | no | 34 | 51 | 40.0 |
|  |  | yes | 15 | 134 | 89.9 |
|  | Overall Percentage | |  |  | 71.8 |
| Step 12 | ICU or CCU Yes:1 No:0 | no | 32 | 53 | 37.6 |
|  |  | yes | 16 | 133 | 89.3 |
|  | Overall Percentage | |  |  | 70.5 |
| Step 13 | ICU or CCU Yes:1 No:0 | no | 29 | 56 | 34.1 |
|  |  | yes | 15 | 134 | 89.9 |
|  | Overall Percentage | |  |  | 69.7 |
| Step 14 | ICU or CCU Yes:1 No:0 | no | 29 | 56 | 34.1 |
|  |  | yes | 18 | 131 | 87.9 |
|  | Overall Percentage | |  |  | 68.4 |
| Step 15 | ICU or CCU Yes:1 No:0 | no | 22 | 63 | 25.9 |
|  |  | yes | 17 | 132 | 88.6 |
|  | Overall Percentage | |  |  | 65.8 |
| Step 16 | ICU or CCU Yes:1 No:0 | no | 29 | 56 | 34.1 |
|  |  | yes | 23 | 126 | 84.6 |
|  | Overall Percentage | |  |  | 66.2 |
| a. The cut value is .500 | | | | | |

| **Variables in the Equation** | | | | | | | | | |
| --- | --- | --- | --- | --- | --- | --- | --- | --- | --- |
|  | | B | S.E. | Wald | df | Sig. | Exp(B) | 95% C.I.for EXP(B) | |
|  |  |  |  |  |  |  |  | Lower | Upper |
| Step 1^a^ | SEX Female: 1 Male:0(1) | -1.006 | .315 | 10.173 | 1 | .001 | .366 | .197 | .679 |
|  | Age 1<65 2:≥ 65(1) | .392 | .326 | 1.447 | 1 | .229 | 1.480 | .781 | 2.805 |
|  | Current Smoker Yes:1 No: 0(1) | .351 | .388 | .816 | 1 | .366 | 1.420 | .663 | 3.041 |
|  | History of HTN Yes:1 No:0(1) | .801 | .408 | 3.858 | 1 | .049 | 2.227 | 1.002 | 4.950 |
|  | History of Diabetes Yes:1 No:0(1) | -.164 | .334 | .241 | 1 | .624 | .849 | .441 | 1.634 |
|  | History of Stroke Yes:1 No:0(1) | -.369 | .516 | .513 | 1 | .474 | .691 | .252 | 1.898 |
|  | History of Transient Ischemic Attack Yes:1 No:0(1) | 1.677 | 1.643 | 1.042 | 1 | .307 | 5.352 | .214 | 134.070 |
|  | History of Peripheral Vascular Disease Yes:1 No:0(1) | -.771 | .550 | 1.969 | 1 | .161 | .462 | .157 | 1.358 |
|  | History of Cancer Yes:1 No:0(1) | .421 | .385 | 1.200 | 1 | .273 | 1.524 | .717 | 3.239 |
|  | History of Myocardial Infarction Yes:1 No:0(1) | -.689 | .426 | 2.618 | 1 | .106 | .502 | .218 | 1.157 |
|  | History of Percutaneous Coronary Intervention Yes:1 No:0(1) | -.429 | .391 | 1.208 | 1 | .272 | .651 | .303 | 1.400 |
|  | History of CABG Yes:1 No:0(1) | .820 | .541 | 2.296 | 1 | .130 | 2.269 | .786 | 6.550 |
|  | History of CKD Yes:1 No:0(1) | .119 | .422 | .080 | 1 | .777 | 1.127 | .493 | 2.576 |
|  | Taking ACEi, ARNi, or ARB Yes:1 No:0(1) | -.324 | .332 | .951 | 1 | .329 | .723 | .377 | 1.387 |
|  | Taking Diuretics Yes:1 No:0(1) | -.335 | .360 | .865 | 1 | .352 | .715 | .353 | 1.449 |
|  | Heart failure diagnosis (0=No HF, 1= History of HF, 2=New HF) |  |  | 3.992 | 2 | .136 |  |  |  |
|  | Heart failure diagnosis (0=No HF, 1= History of HF, 2=New HF)(1) | -.300 | .451 | .444 | 1 | .505 | .740 | .306 | 1.793 |
|  | Heart failure diagnosis (0=No HF, 1= History of HF, 2=New HF)(2) | -.775 | .388 | 3.990 | 1 | .046 | .460 | .215 | .986 |
|  | In patient diuretic use(1) | .593 | .331 | 3.206 | 1 | .073 | 1.809 | .945 | 3.460 |
|  | Hyponatremia present up to 7 days of hospitalization Yes:1 No:0(1) | .536 | .314 | 2.913 | 1 | .088 | 1.709 | .924 | 3.160 |
|  | Constant | .187 | .452 | .171 | 1 | .680 | 1.205 |  |  |
| Step 2^a^ | SEX Female: 1 Male:0(1) | -1.004 | .315 | 10.140 | 1 | .001 | .366 | .198 | .680 |
|  | Age 1<65 2:≥ 65(1) | .402 | .324 | 1.536 | 1 | .215 | 1.495 | .792 | 2.822 |
|  | Current Smoker Yes:1 No: 0(1) | .344 | .388 | .786 | 1 | .375 | 1.410 | .659 | 3.015 |
|  | History of HTN Yes:1 No:0(1) | .815 | .404 | 4.068 | 1 | .044 | 2.260 | 1.023 | 4.992 |
|  | History of Diabetes Yes:1 No:0(1) | -.151 | .331 | .208 | 1 | .648 | .860 | .450 | 1.645 |
|  | History of Stroke Yes:1 No:0(1) | -.370 | .515 | .516 | 1 | .473 | .691 | .252 | 1.896 |
|  | History of Transient Ischemic Attack Yes:1 No:0(1) | 1.755 | 1.621 | 1.172 | 1 | .279 | 5.783 | .241 | 138.713 |
|  | History of Peripheral Vascular Disease Yes:1 No:0(1) | -.761 | .548 | 1.928 | 1 | .165 | .467 | .160 | 1.368 |
|  | History of Cancer Yes:1 No:0(1) | .425 | .384 | 1.220 | 1 | .269 | 1.529 | .720 | 3.248 |
|  | History of Myocardial Infarction Yes:1 No:0(1) | -.683 | .426 | 2.578 | 1 | .108 | .505 | .219 | 1.163 |
|  | History of Percutaneous Coronary Intervention Yes:1 No:0(1) | -.443 | .387 | 1.307 | 1 | .253 | .642 | .301 | 1.372 |
|  | History of CABG Yes:1 No:0(1) | .841 | .535 | 2.468 | 1 | .116 | 2.319 | .812 | 6.623 |
|  | Taking ACEi, ARNi, or ARB Yes:1 No:0(1) | -.338 | .329 | 1.055 | 1 | .304 | .713 | .374 | 1.359 |
|  | Taking Diuretics Yes:1 No:0(1) | -.327 | .359 | .830 | 1 | .362 | .721 | .357 | 1.457 |
|  | Heart failure diagnosis (0=No HF, 1= History of HF, 2=New HF) |  |  | 3.950 | 2 | .139 |  |  |  |
|  | Heart failure diagnosis (0=No HF, 1= History of HF, 2=New HF)(1) | -.286 | .447 | .408 | 1 | .523 | .752 | .313 | 1.806 |
|  | Heart failure diagnosis (0=No HF, 1= History of HF, 2=New HF)(2) | -.771 | .388 | 3.949 | 1 | .047 | .462 | .216 | .989 |
|  | In patient diuretic use(1) | .592 | .331 | 3.201 | 1 | .074 | 1.807 | .945 | 3.456 |
|  | Hyponatremia present up to 7 days of hospitalization Yes:1 No:0(1) | .537 | .314 | 2.922 | 1 | .087 | 1.710 | .924 | 3.164 |
|  | Constant | .185 | .452 | .167 | 1 | .683 | 1.203 |  |  |
| Step 3^a^ | SEX Female: 1 Male:0(1) | -1.014 | .314 | 10.389 | 1 | .001 | .363 | .196 | .672 |
|  | Age 1<65 2:≥ 65(1) | .375 | .318 | 1.385 | 1 | .239 | 1.455 | .779 | 2.715 |
|  | Current Smoker Yes:1 No: 0(1) | .370 | .384 | .926 | 1 | .336 | 1.447 | .682 | 3.072 |
|  | History of HTN Yes:1 No:0(1) | .805 | .404 | 3.975 | 1 | .046 | 2.237 | 1.014 | 4.937 |
|  | History of Stroke Yes:1 No:0(1) | -.381 | .514 | .551 | 1 | .458 | .683 | .249 | 1.870 |
|  | History of Transient Ischemic Attack Yes:1 No:0(1) | 1.708 | 1.616 | 1.117 | 1 | .291 | 5.517 | .232 | 131.038 |
|  | History of Peripheral Vascular Disease Yes:1 No:0(1) | -.802 | .541 | 2.200 | 1 | .138 | .448 | .155 | 1.294 |
|  | History of Cancer Yes:1 No:0(1) | .449 | .381 | 1.395 | 1 | .238 | 1.567 | .744 | 3.304 |
|  | History of Myocardial Infarction Yes:1 No:0(1) | -.703 | .424 | 2.750 | 1 | .097 | .495 | .216 | 1.136 |
|  | History of Percutaneous Coronary Intervention Yes:1 No:0(1) | -.449 | .387 | 1.350 | 1 | .245 | .638 | .299 | 1.362 |
|  | History of CABG Yes:1 No:0(1) | .825 | .533 | 2.393 | 1 | .122 | 2.281 | .802 | 6.485 |
|  | Taking ACEi, ARNi, or ARB Yes:1 No:0(1) | -.349 | .328 | 1.129 | 1 | .288 | .706 | .371 | 1.342 |
|  | Taking Diuretics Yes:1 No:0(1) | -.324 | .358 | .816 | 1 | .366 | .723 | .358 | 1.460 |
|  | Heart failure diagnosis (0=No HF, 1= History of HF, 2=New HF) |  |  | 3.952 | 2 | .139 |  |  |  |
|  | Heart failure diagnosis (0=No HF, 1= History of HF, 2=New HF)(1) | -.293 | .446 | .431 | 1 | .511 | .746 | .311 | 1.789 |
|  | Heart failure diagnosis (0=No HF, 1= History of HF, 2=New HF)(2) | -.771 | .388 | 3.950 | 1 | .047 | .462 | .216 | .989 |
|  | In patient diuretic use(1) | .579 | .329 | 3.090 | 1 | .079 | 1.784 | .936 | 3.402 |
|  | Hyponatremia present up to 7 days of hospitalization Yes:1 No:0(1) | .531 | .313 | 2.872 | 1 | .090 | 1.701 | .920 | 3.144 |
|  | Constant | .159 | .448 | .126 | 1 | .723 | 1.172 |  |  |
| Step 4^a^ | SEX Female: 1 Male:0(1) | -1.039 | .313 | 11.014 | 1 | <.001 | .354 | .192 | .654 |
|  | Age 1<65 2:≥ 65(1) | .361 | .317 | 1.291 | 1 | .256 | 1.434 | .770 | 2.671 |
|  | Current Smoker Yes:1 No: 0(1) | .379 | .384 | .976 | 1 | .323 | 1.461 | .688 | 3.101 |
|  | History of HTN Yes:1 No:0(1) | .801 | .403 | 3.956 | 1 | .047 | 2.228 | 1.012 | 4.908 |
|  | History of Transient Ischemic Attack Yes:1 No:0(1) | 1.533 | 1.619 | .896 | 1 | .344 | 4.632 | .194 | 110.670 |
|  | History of Peripheral Vascular Disease Yes:1 No:0(1) | -.777 | .541 | 2.061 | 1 | .151 | .460 | .159 | 1.328 |
|  | History of Cancer Yes:1 No:0(1) | .445 | .381 | 1.368 | 1 | .242 | 1.561 | .740 | 3.293 |
|  | History of Myocardial Infarction Yes:1 No:0(1) | -.693 | .424 | 2.670 | 1 | .102 | .500 | .218 | 1.148 |
|  | History of Percutaneous Coronary Intervention Yes:1 No:0(1) | -.477 | .384 | 1.541 | 1 | .214 | .621 | .292 | 1.318 |
|  | History of CABG Yes:1 No:0(1) | .823 | .532 | 2.389 | 1 | .122 | 2.277 | .802 | 6.465 |
|  | Taking ACEi, ARNi, or ARB Yes:1 No:0(1) | -.349 | .328 | 1.135 | 1 | .287 | .705 | .371 | 1.341 |
|  | Taking Diuretics Yes:1 No:0(1) | -.323 | .359 | .813 | 1 | .367 | .724 | .358 | 1.462 |
|  | Heart failure diagnosis (0=No HF, 1= History of HF, 2=New HF) |  |  | 3.859 | 2 | .145 |  |  |  |
|  | Heart failure diagnosis (0=No HF, 1= History of HF, 2=New HF)(1) | -.299 | .444 | .452 | 1 | .502 | .742 | .311 | 1.772 |
|  | Heart failure diagnosis (0=No HF, 1= History of HF, 2=New HF)(2) | -.761 | .387 | 3.855 | 1 | .050 | .467 | .219 | .999 |
|  | In patient diuretic use(1) | .577 | .329 | 3.069 | 1 | .080 | 1.780 | .934 | 3.393 |
|  | Hyponatremia present up to 7 days of hospitalization Yes:1 No:0(1) | .525 | .313 | 2.817 | 1 | .093 | 1.691 | .916 | 3.123 |
|  | Constant | .152 | .446 | .116 | 1 | .733 | 1.164 |  |  |
| Step 5^a^ | SEX Female: 1 Male:0(1) | -1.058 | .313 | 11.450 | 1 | <.001 | .347 | .188 | .641 |
|  | Age 1<65 2:≥ 65(1) | .343 | .316 | 1.177 | 1 | .278 | 1.409 | .758 | 2.619 |
|  | Current Smoker Yes:1 No: 0(1) | .394 | .382 | 1.062 | 1 | .303 | 1.483 | .701 | 3.137 |
|  | History of HTN Yes:1 No:0(1) | .756 | .399 | 3.598 | 1 | .058 | 2.130 | .975 | 4.651 |
|  | History of Transient Ischemic Attack Yes:1 No:0(1) | 1.301 | 1.594 | .666 | 1 | .414 | 3.673 | .161 | 83.587 |
|  | History of Peripheral Vascular Disease Yes:1 No:0(1) | -.824 | .540 | 2.327 | 1 | .127 | .439 | .152 | 1.265 |
|  | History of Cancer Yes:1 No:0(1) | .417 | .378 | 1.218 | 1 | .270 | 1.518 | .723 | 3.185 |
|  | History of Myocardial Infarction Yes:1 No:0(1) | -.716 | .423 | 2.856 | 1 | .091 | .489 | .213 | 1.121 |
|  | History of Percutaneous Coronary Intervention Yes:1 No:0(1) | -.441 | .382 | 1.334 | 1 | .248 | .643 | .304 | 1.360 |
|  | History of CABG Yes:1 No:0(1) | .788 | .531 | 2.202 | 1 | .138 | 2.200 | .777 | 6.230 |
|  | Taking ACEi, ARNi, or ARB Yes:1 No:0(1) | -.378 | .326 | 1.343 | 1 | .247 | .686 | .362 | 1.298 |
|  | Heart failure diagnosis (0=No HF, 1= History of HF, 2=New HF) |  |  | 3.811 | 2 | .149 |  |  |  |
|  | Heart failure diagnosis (0=No HF, 1= History of HF, 2=New HF)(1) | -.350 | .441 | .631 | 1 | .427 | .704 | .297 | 1.673 |
|  | Heart failure diagnosis (0=No HF, 1= History of HF, 2=New HF)(2) | -.750 | .386 | 3.776 | 1 | .052 | .472 | .222 | 1.006 |
|  | In patient diuretic use(1) | .503 | .317 | 2.510 | 1 | .113 | 1.653 | .888 | 3.079 |
|  | Hyponatremia present up to 7 days of hospitalization Yes:1 No:0(1) | .533 | .313 | 2.899 | 1 | .089 | 1.704 | .923 | 3.147 |
|  | Constant | .164 | .446 | .136 | 1 | .712 | 1.179 |  |  |
| Step 6^a^ | SEX Female: 1 Male:0(1) | -1.036 | .310 | 11.127 | 1 | <.001 | .355 | .193 | .652 |
|  | Age 1<65 2:≥ 65(1) | .329 | .315 | 1.090 | 1 | .296 | 1.389 | .749 | 2.576 |
|  | Current Smoker Yes:1 No: 0(1) | .387 | .382 | 1.023 | 1 | .312 | 1.472 | .696 | 3.114 |
|  | History of HTN Yes:1 No:0(1) | .765 | .397 | 3.710 | 1 | .054 | 2.150 | .987 | 4.683 |
|  | History of Peripheral Vascular Disease Yes:1 No:0(1) | -.706 | .519 | 1.851 | 1 | .174 | .493 | .178 | 1.365 |
|  | History of Cancer Yes:1 No:0(1) | .389 | .375 | 1.077 | 1 | .299 | 1.476 | .708 | 3.078 |
|  | History of Myocardial Infarction Yes:1 No:0(1) | -.681 | .420 | 2.628 | 1 | .105 | .506 | .222 | 1.153 |
|  | History of Percutaneous Coronary Intervention Yes:1 No:0(1) | -.416 | .380 | 1.198 | 1 | .274 | .660 | .313 | 1.389 |
|  | History of CABG Yes:1 No:0(1) | .715 | .521 | 1.882 | 1 | .170 | 2.044 | .736 | 5.674 |
|  | Taking ACEi, ARNi, or ARB Yes:1 No:0(1) | -.395 | .324 | 1.490 | 1 | .222 | .673 | .357 | 1.271 |
|  | Heart failure diagnosis (0=No HF, 1= History of HF, 2=New HF) |  |  | 3.878 | 2 | .144 |  |  |  |
|  | Heart failure diagnosis (0=No HF, 1= History of HF, 2=New HF)(1) | -.317 | .439 | .521 | 1 | .471 | .729 | .308 | 1.722 |
|  | Heart failure diagnosis (0=No HF, 1= History of HF, 2=New HF)(2) | -.757 | .385 | 3.868 | 1 | .049 | .469 | .221 | .997 |
|  | In patient diuretic use(1) | .477 | .315 | 2.299 | 1 | .129 | 1.612 | .870 | 2.987 |
|  | Hyponatremia present up to 7 days of hospitalization Yes:1 No:0(1) | .541 | .312 | 3.002 | 1 | .083 | 1.718 | .931 | 3.170 |
|  | Constant | .163 | .445 | .135 | 1 | .713 | 1.177 |  |  |
| Step 7^a^ | SEX Female: 1 Male:0(1) | -1.017 | .310 | 10.800 | 1 | .001 | .362 | .197 | .663 |
|  | Age 1<65 2:≥ 65(1) | .291 | .313 | .869 | 1 | .351 | 1.338 | .725 | 2.469 |
|  | History of HTN Yes:1 No:0(1) | .704 | .391 | 3.251 | 1 | .071 | 2.022 | .941 | 4.348 |
|  | History of Peripheral Vascular Disease Yes:1 No:0(1) | -.685 | .520 | 1.734 | 1 | .188 | .504 | .182 | 1.397 |
|  | History of Cancer Yes:1 No:0(1) | .301 | .364 | .685 | 1 | .408 | 1.351 | .662 | 2.755 |
|  | History of Myocardial Infarction Yes:1 No:0(1) | -.601 | .412 | 2.128 | 1 | .145 | .548 | .245 | 1.229 |
|  | History of Percutaneous Coronary Intervention Yes:1 No:0(1) | -.421 | .380 | 1.225 | 1 | .268 | .656 | .311 | 1.383 |
|  | History of CABG Yes:1 No:0(1) | .635 | .513 | 1.531 | 1 | .216 | 1.886 | .690 | 5.153 |
|  | Taking ACEi, ARNi, or ARB Yes:1 No:0(1) | -.405 | .324 | 1.571 | 1 | .210 | .667 | .354 | 1.257 |
|  | Heart failure diagnosis (0=No HF, 1= History of HF, 2=New HF) |  |  | 3.840 | 2 | .147 |  |  |  |
|  | Heart failure diagnosis (0=No HF, 1= History of HF, 2=New HF)(1) | -.347 | .437 | .631 | 1 | .427 | .707 | .300 | 1.664 |
|  | Heart failure diagnosis (0=No HF, 1= History of HF, 2=New HF)(2) | -.750 | .384 | 3.810 | 1 | .051 | .472 | .222 | 1.003 |
|  | In patient diuretic use(1) | .461 | .314 | 2.158 | 1 | .142 | 1.585 | .857 | 2.932 |
|  | Hyponatremia present up to 7 days of hospitalization Yes:1 No:0(1) | .604 | .306 | 3.882 | 1 | .049 | 1.829 | 1.003 | 3.335 |
|  | Constant | .303 | .421 | .519 | 1 | .471 | 1.354 |  |  |
| Step 8^a^ | SEX Female: 1 Male:0(1) | -.993 | .307 | 10.467 | 1 | .001 | .371 | .203 | .676 |
|  | Age 1<65 2:≥ 65(1) | .330 | .309 | 1.144 | 1 | .285 | 1.391 | .760 | 2.547 |
|  | History of HTN Yes:1 No:0(1) | .690 | .389 | 3.139 | 1 | .076 | 1.993 | .929 | 4.276 |
|  | History of Peripheral Vascular Disease Yes:1 No:0(1) | -.641 | .516 | 1.544 | 1 | .214 | .527 | .192 | 1.448 |
|  | History of Myocardial Infarction Yes:1 No:0(1) | -.603 | .411 | 2.147 | 1 | .143 | .547 | .244 | 1.226 |
|  | History of Percutaneous Coronary Intervention Yes:1 No:0(1) | -.416 | .381 | 1.194 | 1 | .274 | .660 | .313 | 1.391 |
|  | History of CABG Yes:1 No:0(1) | .606 | .511 | 1.403 | 1 | .236 | 1.832 | .673 | 4.990 |
|  | Taking ACEi, ARNi, or ARB Yes:1 No:0(1) | -.421 | .323 | 1.703 | 1 | .192 | .656 | .349 | 1.235 |
|  | Heart failure diagnosis (0=No HF, 1= History of HF, 2=New HF) |  |  | 3.913 | 2 | .141 |  |  |  |
|  | Heart failure diagnosis (0=No HF, 1= History of HF, 2=New HF)(1) | -.339 | .436 | .605 | 1 | .437 | .713 | .303 | 1.674 |
|  | Heart failure diagnosis (0=No HF, 1= History of HF, 2=New HF)(2) | -.757 | .384 | 3.892 | 1 | .049 | .469 | .221 | .995 |
|  | In patient diuretic use(1) | .452 | .313 | 2.092 | 1 | .148 | 1.572 | .852 | 2.902 |
|  | Hyponatremia present up to 7 days of hospitalization Yes:1 No:0(1) | .635 | .304 | 4.358 | 1 | .037 | 1.887 | 1.040 | 3.425 |
|  | Constant | .341 | .417 | .670 | 1 | .413 | 1.407 |  |  |
| Step 9^a^ | SEX Female: 1 Male:0(1) | -.948 | .302 | 9.848 | 1 | .002 | .388 | .214 | .701 |
|  | History of HTN Yes:1 No:0(1) | .728 | .388 | 3.512 | 1 | .061 | 2.070 | .967 | 4.431 |
|  | History of Peripheral Vascular Disease Yes:1 No:0(1) | -.688 | .513 | 1.801 | 1 | .180 | .503 | .184 | 1.373 |
|  | History of Myocardial Infarction Yes:1 No:0(1) | -.612 | .408 | 2.246 | 1 | .134 | .542 | .244 | 1.207 |
|  | History of Percutaneous Coronary Intervention Yes:1 No:0(1) | -.436 | .377 | 1.335 | 1 | .248 | .647 | .309 | 1.355 |
|  | History of CABG Yes:1 No:0(1) | .672 | .504 | 1.775 | 1 | .183 | 1.958 | .729 | 5.261 |
|  | Taking ACEi, ARNi, or ARB Yes:1 No:0(1) | -.395 | .320 | 1.524 | 1 | .217 | .674 | .360 | 1.261 |
|  | Heart failure diagnosis (0=No HF, 1= History of HF, 2=New HF) |  |  | 3.622 | 2 | .163 |  |  |  |
|  | Heart failure diagnosis (0=No HF, 1= History of HF, 2=New HF)(1) | -.284 | .429 | .438 | 1 | .508 | .753 | .325 | 1.745 |
|  | Heart failure diagnosis (0=No HF, 1= History of HF, 2=New HF)(2) | -.728 | .383 | 3.619 | 1 | .057 | .483 | .228 | 1.022 |
|  | In patient diuretic use(1) | .478 | .311 | 2.365 | 1 | .124 | 1.614 | .877 | 2.969 |
|  | Hyponatremia present up to 7 days of hospitalization Yes:1 No:0(1) | .603 | .302 | 4.003 | 1 | .045 | 1.828 | 1.012 | 3.302 |
|  | Constant | .439 | .408 | 1.159 | 1 | .282 | 1.551 |  |  |
| Step 10^a^ | SEX Female: 1 Male:0(1) | -.934 | .301 | 9.660 | 1 | .002 | .393 | .218 | .708 |
|  | History of HTN Yes:1 No:0(1) | .660 | .384 | 2.954 | 1 | .086 | 1.935 | .912 | 4.106 |
|  | History of Peripheral Vascular Disease Yes:1 No:0(1) | -.733 | .511 | 2.054 | 1 | .152 | .481 | .176 | 1.309 |
|  | History of Myocardial Infarction Yes:1 No:0(1) | -.797 | .378 | 4.451 | 1 | .035 | .451 | .215 | .945 |
|  | History of CABG Yes:1 No:0(1) | .576 | .501 | 1.322 | 1 | .250 | 1.779 | .667 | 4.747 |
|  | Taking ACEi, ARNi, or ARB Yes:1 No:0(1) | -.419 | .318 | 1.733 | 1 | .188 | .658 | .353 | 1.227 |
|  | Heart failure diagnosis (0=No HF, 1= History of HF, 2=New HF) |  |  | 3.985 | 2 | .136 |  |  |  |
|  | Heart failure diagnosis (0=No HF, 1= History of HF, 2=New HF)(1) | -.293 | .429 | .469 | 1 | .494 | .746 | .322 | 1.727 |
|  | Heart failure diagnosis (0=No HF, 1= History of HF, 2=New HF)(2) | -.762 | .382 | 3.982 | 1 | .046 | .467 | .221 | .987 |
|  | In patient diuretic use(1) | .478 | .310 | 2.381 | 1 | .123 | 1.613 | .879 | 2.961 |
|  | Hyponatremia present up to 7 days of hospitalization Yes:1 No:0(1) | .598 | .301 | 3.944 | 1 | .047 | 1.818 | 1.008 | 3.280 |
|  | Constant | .435 | .408 | 1.136 | 1 | .287 | 1.545 |  |  |
| Step 11^a^ | SEX Female: 1 Male:0(1) | -.943 | .300 | 9.881 | 1 | .002 | .389 | .216 | .701 |
|  | History of HTN Yes:1 No:0(1) | .721 | .380 | 3.603 | 1 | .058 | 2.057 | .977 | 4.330 |
|  | History of Peripheral Vascular Disease Yes:1 No:0(1) | -.651 | .499 | 1.701 | 1 | .192 | .522 | .196 | 1.387 |
|  | History of Myocardial Infarction Yes:1 No:0(1) | -.715 | .366 | 3.808 | 1 | .051 | .489 | .239 | 1.003 |
|  | Taking ACEi, ARNi, or ARB Yes:1 No:0(1) | -.436 | .317 | 1.893 | 1 | .169 | .647 | .348 | 1.203 |
|  | Heart failure diagnosis (0=No HF, 1= History of HF, 2=New HF) |  |  | 3.852 | 2 | .146 |  |  |  |
|  | Heart failure diagnosis (0=No HF, 1= History of HF, 2=New HF)(1) | -.169 | .413 | .167 | 1 | .683 | .845 | .376 | 1.897 |
|  | Heart failure diagnosis (0=No HF, 1= History of HF, 2=New HF)(2) | -.741 | .380 | 3.805 | 1 | .051 | .477 | .226 | 1.004 |
|  | In patient diuretic use(1) | .478 | .309 | 2.398 | 1 | .121 | 1.613 | .881 | 2.956 |
|  | Hyponatremia present up to 7 days of hospitalization Yes:1 No:0(1) | .631 | .299 | 4.456 | 1 | .035 | 1.880 | 1.046 | 3.377 |
|  | Constant | .393 | .405 | .946 | 1 | .331 | 1.482 |  |  |
| Step 12^a^ | SEX Female: 1 Male:0(1) | -.907 | .297 | 9.314 | 1 | .002 | .404 | .226 | .723 |
|  | History of HTN Yes:1 No:0(1) | .654 | .375 | 3.050 | 1 | .081 | 1.924 | .923 | 4.010 |
|  | History of Myocardial Infarction Yes:1 No:0(1) | -.783 | .362 | 4.673 | 1 | .031 | .457 | .225 | .930 |
|  | Taking ACEi, ARNi, or ARB Yes:1 No:0(1) | -.409 | .315 | 1.690 | 1 | .194 | .664 | .358 | 1.231 |
|  | Heart failure diagnosis (0=No HF, 1= History of HF, 2=New HF) |  |  | 4.242 | 2 | .120 |  |  |  |
|  | Heart failure diagnosis (0=No HF, 1= History of HF, 2=New HF)(1) | -.211 | .410 | .263 | 1 | .608 | .810 | .362 | 1.811 |
|  | Heart failure diagnosis (0=No HF, 1= History of HF, 2=New HF)(2) | -.779 | .379 | 4.225 | 1 | .040 | .459 | .218 | .964 |
|  | In patient diuretic use(1) | .514 | .307 | 2.792 | 1 | .095 | 1.671 | .915 | 3.054 |
|  | Hyponatremia present up to 7 days of hospitalization Yes:1 No:0(1) | .648 | .298 | 4.741 | 1 | .029 | 1.912 | 1.067 | 3.427 |
|  | Constant | .359 | .402 | .798 | 1 | .372 | 1.432 |  |  |
| Step 13^a^ | SEX Female: 1 Male:0(1) | -.931 | .296 | 9.924 | 1 | .002 | .394 | .221 | .703 |
|  | History of HTN Yes:1 No:0(1) | .535 | .361 | 2.198 | 1 | .138 | 1.708 | .842 | 3.466 |
|  | History of Myocardial Infarction Yes:1 No:0(1) | -.763 | .360 | 4.504 | 1 | .034 | .466 | .230 | .943 |
|  | Heart failure diagnosis (0=No HF, 1= History of HF, 2=New HF) |  |  | 4.137 | 2 | .126 |  |  |  |
|  | Heart failure diagnosis (0=No HF, 1= History of HF, 2=New HF)(1) | -.243 | .408 | .354 | 1 | .552 | .784 | .352 | 1.746 |
|  | Heart failure diagnosis (0=No HF, 1= History of HF, 2=New HF)(2) | -.767 | .377 | 4.135 | 1 | .042 | .465 | .222 | .973 |
|  | In patient diuretic use(1) | .467 | .303 | 2.376 | 1 | .123 | 1.596 | .881 | 2.891 |
|  | Hyponatremia present up to 7 days of hospitalization Yes:1 No:0(1) | .660 | .296 | 4.954 | 1 | .026 | 1.934 | 1.082 | 3.458 |
|  | Constant | .336 | .401 | .701 | 1 | .403 | 1.399 |  |  |
| Step 14^a^ | SEX Female: 1 Male:0(1) | -.919 | .294 | 9.771 | 1 | .002 | .399 | .224 | .710 |
|  | History of Myocardial Infarction Yes:1 No:0(1) | -.697 | .355 | 3.846 | 1 | .050 | .498 | .248 | 1.000 |
|  | Heart failure diagnosis (0=No HF, 1= History of HF, 2=New HF) |  |  | 3.766 | 2 | .152 |  |  |  |
|  | Heart failure diagnosis (0=No HF, 1= History of HF, 2=New HF)(1) | -.274 | .404 | .458 | 1 | .499 | .761 | .344 | 1.680 |
|  | Heart failure diagnosis (0=No HF, 1= History of HF, 2=New HF)(2) | -.726 | .374 | 3.762 | 1 | .052 | .484 | .232 | 1.008 |
|  | In patient diuretic use(1) | .508 | .300 | 2.857 | 1 | .091 | 1.661 | .922 | 2.992 |
|  | Hyponatremia present up to 7 days of hospitalization Yes:1 No:0(1) | .666 | .295 | 5.102 | 1 | .024 | 1.947 | 1.092 | 3.470 |
|  | Constant | .715 | .311 | 5.270 | 1 | .022 | 2.043 |  |  |
| Step 15^a^ | SEX Female: 1 Male:0(1) | -.867 | .289 | 8.972 | 1 | .003 | .420 | .238 | .741 |
|  | History of Myocardial Infarction Yes:1 No:0(1) | -.733 | .336 | 4.744 | 1 | .029 | .481 | .249 | .929 |
|  | In patient diuretic use(1) | .379 | .286 | 1.758 | 1 | .185 | 1.461 | .834 | 2.561 |
|  | Hyponatremia present up to 7 days of hospitalization Yes:1 No:0(1) | .577 | .287 | 4.045 | 1 | .044 | 1.781 | 1.015 | 3.124 |
|  | Constant | .607 | .303 | 4.003 | 1 | .045 | 1.835 |  |  |
| Step 16^a^ | SEX Female: 1 Male:0(1) | -.837 | .287 | 8.506 | 1 | .004 | .433 | .247 | .760 |
|  | History of Myocardial Infarction Yes:1 No:0(1) | -.696 | .333 | 4.353 | 1 | .037 | .499 | .259 | .959 |
|  | Hyponatremia present up to 7 days of hospitalization Yes:1 No:0(1) | .605 | .285 | 4.497 | 1 | .034 | 1.832 | 1.047 | 3.205 |
|  | Constant | .765 | .281 | 7.421 | 1 | .006 | 2.149 |  |  |
| a. Variable(s) entered on step 1: SEX Female: 1 Male:0, Age 1<65 2:≥ 65, Current Smoker Yes:1 No: 0, History of HTN Yes:1 No:0, History of Diabetes Yes:1 No:0, History of Stroke Yes:1 No:0, History of Transient Ischemic Attack Yes:1 No:0, History of Peripheral Vascular Disease Yes:1 No:0, History of Cancer Yes:1 No:0, History of Myocardial Infarction Yes:1 No:0, History of Percutaneous Coronary Intervention Yes:1 No:0, History of CABG Yes:1 No:0, History of CKD Yes:1 No:0, Taking ACEi, ARNi, or ARB Yes:1 No:0, Taking Diuretics Yes:1 No:0, Heart failure diagnosis (0=No HF, 1= History of HF, 2=New HF), In patient diuretic use, Hyponatremia present up to 7 days of hospitalization Yes:1 No:0. | | | | | | | | | |

**Dependent variable: New heart failure diagnosis**

**Independent variable: Hyponatremia up to 7 days of admission**

| **Omnibus Tests of Model Coefficients** | | | | |
| --- | --- | --- | --- | --- |
|  | | Chi-square | df | Sig. |
| Step 1 | Step | 23.783 | 17 | .125 |
|  | Block | 23.783 | 17 | .125 |
|  | Model | 23.783 | 17 | .125 |
| Step 2^a^ | Step | .000 | 1 | .997 |
|  | Block | 23.783 | 16 | .094 |
|  | Model | 23.783 | 16 | .094 |
| Step 3^a^ | Step | -.024 | 1 | .876 |
|  | Block | 23.759 | 15 | .069 |
|  | Model | 23.759 | 15 | .069 |
| Step 4^a^ | Step | -.036 | 1 | .850 |
|  | Block | 23.723 | 14 | .049 |
|  | Model | 23.723 | 14 | .049 |
| Step 5^a^ | Step | -.068 | 1 | .794 |
|  | Block | 23.655 | 13 | .034 |
|  | Model | 23.655 | 13 | .034 |
| Step 6^a^ | Step | -.082 | 1 | .775 |
|  | Block | 23.573 | 12 | .023 |
|  | Model | 23.573 | 12 | .023 |
| Step 7^a^ | Step | -.149 | 1 | .699 |
|  | Block | 23.424 | 11 | .015 |
|  | Model | 23.424 | 11 | .015 |
| Step 8^a^ | Step | -.448 | 1 | .503 |
|  | Block | 22.976 | 10 | .011 |
|  | Model | 22.976 | 10 | .011 |
| Step 9^a^ | Step | -.380 | 1 | .537 |
|  | Block | 22.596 | 9 | .007 |
|  | Model | 22.596 | 9 | .007 |
| Step 10^a^ | Step | -.616 | 1 | .432 |
|  | Block | 21.980 | 8 | .005 |
|  | Model | 21.980 | 8 | .005 |
| Step 11^a^ | Step | -.589 | 1 | .443 |
|  | Block | 21.391 | 7 | .003 |
|  | Model | 21.391 | 7 | .003 |
| Step 12^a^ | Step | -.807 | 1 | .369 |
|  | Block | 20.583 | 6 | .002 |
|  | Model | 20.583 | 6 | .002 |
| Step 13^a^ | Step | -.927 | 1 | .336 |
|  | Block | 19.656 | 5 | .001 |
|  | Model | 19.656 | 5 | .001 |
| Step 14^a^ | Step | -.699 | 1 | .403 |
|  | Block | 18.957 | 4 | <.001 |
|  | Model | 18.957 | 4 | <.001 |
| Step 15^a^ | Step | -1.672 | 1 | .196 |
|  | Block | 17.285 | 3 | <.001 |
|  | Model | 17.285 | 3 | <.001 |
| a. A negative Chi-squares value indicates that the Chi-squares value has decreased from the previous step. | | | | |

| **Hosmer and Lemeshow Test** | | | |
| --- | --- | --- | --- |
| Step | Chi-square | df | Sig. |
| 1 | 4.489 | 8 | .810 |
| 2 | 4.490 | 8 | .810 |
| 3 | 6.664 | 8 | .573 |
| 4 | 3.951 | 8 | .861 |
| 5 | 2.341 | 8 | .969 |
| 6 | 2.679 | 8 | .953 |
| 7 | 5.654 | 8 | .686 |
| 8 | 4.597 | 8 | .800 |
| 9 | 6.869 | 8 | .551 |
| 10 | 7.522 | 8 | .482 |
| 11 | 7.304 | 8 | .504 |
| 12 | 5.843 | 8 | .665 |
| 13 | 3.098 | 7 | .876 |
| 14 | 2.988 | 6 | .810 |
| 15 | 6.621 | 5 | .250 |

| **Classification Table**^a^ | | | | | |
| --- | --- | --- | --- | --- | --- |
|  | Observed | | Predicted | | |
|  |  |  | New HF 0=no, 1=yes | | Percentage Correct |
|  |  |  | No | Yes |  |
| Step 1 | New HF 0=no, 1=yes | No | 132 | 8 | 94.3 |
|  |  | Yes | 41 | 8 | 16.3 |
|  | Overall Percentage | |  |  | 74.1 |
| Step 2 | New HF 0=no, 1=yes | No | 132 | 8 | 94.3 |
|  |  | Yes | 41 | 8 | 16.3 |
|  | Overall Percentage | |  |  | 74.1 |
| Step 3 | New HF 0=no, 1=yes | No | 131 | 9 | 93.6 |
|  |  | Yes | 41 | 8 | 16.3 |
|  | Overall Percentage | |  |  | 73.5 |
| Step 4 | New HF 0=no, 1=yes | No | 132 | 8 | 94.3 |
|  |  | Yes | 42 | 7 | 14.3 |
|  | Overall Percentage | |  |  | 73.5 |
| Step 5 | New HF 0=no, 1=yes | No | 132 | 8 | 94.3 |
|  |  | Yes | 43 | 6 | 12.2 |
|  | Overall Percentage | |  |  | 73.0 |
| Step 6 | New HF 0=no, 1=yes | No | 132 | 8 | 94.3 |
|  |  | Yes | 43 | 6 | 12.2 |
|  | Overall Percentage | |  |  | 73.0 |
| Step 7 | New HF 0=no, 1=yes | No | 131 | 9 | 93.6 |
|  |  | Yes | 40 | 9 | 18.4 |
|  | Overall Percentage | |  |  | 74.1 |
| Step 8 | New HF 0=no, 1=yes | No | 131 | 9 | 93.6 |
|  |  | Yes | 40 | 9 | 18.4 |
|  | Overall Percentage | |  |  | 74.1 |
| Step 9 | New HF 0=no, 1=yes | No | 131 | 9 | 93.6 |
|  |  | Yes | 40 | 9 | 18.4 |
|  | Overall Percentage | |  |  | 74.1 |
| Step 10 | New HF 0=no, 1=yes | No | 131 | 9 | 93.6 |
|  |  | Yes | 41 | 8 | 16.3 |
|  | Overall Percentage | |  |  | 73.5 |
| Step 11 | New HF 0=no, 1=yes | No | 134 | 6 | 95.7 |
|  |  | Yes | 42 | 7 | 14.3 |
|  | Overall Percentage | |  |  | 74.6 |
| Step 12 | New HF 0=no, 1=yes | No | 133 | 7 | 95.0 |
|  |  | Yes | 41 | 8 | 16.3 |
|  | Overall Percentage | |  |  | 74.6 |
| Step 13 | New HF 0=no, 1=yes | No | 132 | 8 | 94.3 |
|  |  | Yes | 43 | 6 | 12.2 |
|  | Overall Percentage | |  |  | 73.0 |
| Step 14 | New HF 0=no, 1=yes | No | 132 | 8 | 94.3 |
|  |  | Yes | 41 | 8 | 16.3 |
|  | Overall Percentage | |  |  | 74.1 |
| Step 15 | New HF 0=no, 1=yes | No | 133 | 7 | 95.0 |
|  |  | Yes | 42 | 7 | 14.3 |
|  | Overall Percentage | |  |  | 74.1 |
| a. The cut value is .500 | | | | | |

| **Variables in the Equation** | | | | | | | | | |
| --- | --- | --- | --- | --- | --- | --- | --- | --- | --- |
|  | | B | S.E. | Wald | df | Sig. | Exp(B) | 95% C.I.for EXP(B) | |
|  |  |  |  |  |  |  |  | Lower | Upper |
| Step 1^a^ | SEX Female: 1 Male:0(1) | -.337 | .388 | .753 | 1 | .386 | .714 | .334 | 1.528 |
|  | Age 1<65 2:≥ 65(1) | .324 | .405 | .642 | 1 | .423 | 1.383 | .625 | 3.059 |
|  | Current Smoker Yes:1 No: 0(1) | .277 | .455 | .372 | 1 | .542 | 1.320 | .541 | 3.218 |
|  | History of HTN Yes:1 No:0(1) | .362 | .561 | .416 | 1 | .519 | 1.436 | .478 | 4.316 |
|  | History of Diabetes Yes:1 No:0(1) | .060 | .388 | .024 | 1 | .877 | 1.062 | .497 | 2.270 |
|  | History of Stroke Yes:1 No:0(1) | -.589 | .717 | .675 | 1 | .411 | .555 | .136 | 2.261 |
|  | History of Transient Ischemic Attack Yes:1 No:0(1) | -20.307 | 40192.970 | .000 | 1 | 1.000 | .000 | .000 | . |
|  | History of Peripheral Vascular Disease Yes:1 No:0(1) | .717 | .697 | 1.056 | 1 | .304 | 2.048 | .522 | 8.036 |
|  | History of Cancer Yes:1 No:0(1) | -.106 | .467 | .051 | 1 | .821 | .899 | .360 | 2.247 |
|  | History of Myocardial Infarction Yes:1 No:0(1) | -.110 | .561 | .038 | 1 | .845 | .896 | .298 | 2.691 |
|  | History of Percutaneous Coronary Intervention Yes:1 No:0(1) | .626 | .478 | 1.713 | 1 | .191 | 1.870 | .732 | 4.774 |
|  | History of CABG Yes:1 No:0(1) | .209 | .674 | .096 | 1 | .757 | 1.232 | .329 | 4.620 |
|  | History of CKD Yes:1 No:0(1) | .405 | .502 | .650 | 1 | .420 | 1.499 | .560 | 4.010 |
|  | Taking ACEi, ARNi, or ARB Yes:1 No:0(1) | -.002 | .407 | .000 | 1 | .997 | .998 | .450 | 2.215 |
|  | Taking Diuretics Yes:1 No:0(1) | -.171 | .453 | .143 | 1 | .705 | .843 | .347 | 2.046 |
|  | In patient diuretic use(1) | 1.067 | .383 | 7.759 | 1 | .005 | 2.905 | 1.372 | 6.153 |
|  | Hyponatremia present up to 7 days of hospitalization Yes:1 No:0(1) | .747 | .402 | 3.450 | 1 | .063 | 2.111 | .960 | 4.646 |
|  | Constant | -2.725 | .676 | 16.228 | 1 | <.001 | .066 |  |  |
| Step 2^a^ | SEX Female: 1 Male:0(1) | -.337 | .388 | .755 | 1 | .385 | .714 | .334 | 1.527 |
|  | Age 1<65 2:≥ 65(1) | .324 | .404 | .644 | 1 | .422 | 1.383 | .626 | 3.054 |
|  | Current Smoker Yes:1 No: 0(1) | .277 | .455 | .372 | 1 | .542 | 1.320 | .541 | 3.217 |
|  | History of HTN Yes:1 No:0(1) | .362 | .552 | .429 | 1 | .513 | 1.436 | .486 | 4.238 |
|  | History of Diabetes Yes:1 No:0(1) | .060 | .384 | .024 | 1 | .876 | 1.062 | .500 | 2.254 |
|  | History of Stroke Yes:1 No:0(1) | -.589 | .717 | .675 | 1 | .411 | .555 | .136 | 2.261 |
|  | History of Transient Ischemic Attack Yes:1 No:0(1) | -20.306 | 40192.970 | .000 | 1 | 1.000 | .000 | .000 | . |
|  | History of Peripheral Vascular Disease Yes:1 No:0(1) | .717 | .695 | 1.065 | 1 | .302 | 2.048 | .525 | 7.994 |
|  | History of Cancer Yes:1 No:0(1) | -.106 | .466 | .052 | 1 | .820 | .900 | .361 | 2.244 |
|  | History of Myocardial Infarction Yes:1 No:0(1) | -.110 | .560 | .039 | 1 | .844 | .896 | .299 | 2.685 |
|  | History of Percutaneous Coronary Intervention Yes:1 No:0(1) | .626 | .472 | 1.756 | 1 | .185 | 1.869 | .741 | 4.715 |
|  | History of CABG Yes:1 No:0(1) | .209 | .673 | .096 | 1 | .756 | 1.232 | .330 | 4.607 |
|  | History of CKD Yes:1 No:0(1) | .405 | .499 | .660 | 1 | .417 | 1.499 | .564 | 3.983 |
|  | Taking Diuretics Yes:1 No:0(1) | -.172 | .448 | .147 | 1 | .702 | .842 | .350 | 2.027 |
|  | In patient diuretic use(1) | 1.067 | .382 | 7.778 | 1 | .005 | 2.906 | 1.373 | 6.148 |
|  | Hyponatremia present up to 7 days of hospitalization Yes:1 No:0(1) | .747 | .402 | 3.450 | 1 | .063 | 2.111 | .960 | 4.646 |
|  | Constant | -2.725 | .676 | 16.243 | 1 | <.001 | .066 |  |  |
| Step 3^a^ | SEX Female: 1 Male:0(1) | -.335 | .388 | .748 | 1 | .387 | .715 | .335 | 1.529 |
|  | Age 1<65 2:≥ 65(1) | .332 | .401 | .688 | 1 | .407 | 1.394 | .636 | 3.058 |
|  | Current Smoker Yes:1 No: 0(1) | .267 | .449 | .352 | 1 | .553 | 1.306 | .541 | 3.150 |
|  | History of HTN Yes:1 No:0(1) | .367 | .551 | .443 | 1 | .506 | 1.443 | .490 | 4.246 |
|  | History of Stroke Yes:1 No:0(1) | -.589 | .718 | .675 | 1 | .411 | .555 | .136 | 2.264 |
|  | History of Transient Ischemic Attack Yes:1 No:0(1) | -20.286 | 40192.970 | .000 | 1 | 1.000 | .000 | .000 | . |
|  | History of Peripheral Vascular Disease Yes:1 No:0(1) | .734 | .686 | 1.144 | 1 | .285 | 2.084 | .543 | 7.997 |
|  | History of Cancer Yes:1 No:0(1) | -.116 | .462 | .063 | 1 | .802 | .891 | .360 | 2.203 |
|  | History of Myocardial Infarction Yes:1 No:0(1) | -.105 | .560 | .035 | 1 | .851 | .900 | .301 | 2.695 |
|  | History of Percutaneous Coronary Intervention Yes:1 No:0(1) | .629 | .472 | 1.777 | 1 | .182 | 1.876 | .744 | 4.732 |
|  | History of CABG Yes:1 No:0(1) | .208 | .673 | .095 | 1 | .757 | 1.231 | .329 | 4.600 |
|  | History of CKD Yes:1 No:0(1) | .409 | .498 | .675 | 1 | .411 | 1.505 | .567 | 3.995 |
|  | Taking Diuretics Yes:1 No:0(1) | -.171 | .448 | .146 | 1 | .702 | .843 | .350 | 2.028 |
|  | In patient diuretic use(1) | 1.072 | .381 | 7.925 | 1 | .005 | 2.922 | 1.385 | 6.163 |
|  | Hyponatremia present up to 7 days of hospitalization Yes:1 No:0(1) | .748 | .402 | 3.466 | 1 | .063 | 2.114 | .961 | 4.648 |
|  | Constant | -2.711 | .669 | 16.426 | 1 | <.001 | .066 |  |  |
| Step 4^a^ | SEX Female: 1 Male:0(1) | -.333 | .387 | .739 | 1 | .390 | .717 | .335 | 1.532 |
|  | Age 1<65 2:≥ 65(1) | .337 | .400 | .710 | 1 | .399 | 1.401 | .640 | 3.066 |
|  | Current Smoker Yes:1 No: 0(1) | .255 | .445 | .329 | 1 | .566 | 1.291 | .539 | 3.087 |
|  | History of HTN Yes:1 No:0(1) | .365 | .550 | .441 | 1 | .507 | 1.441 | .490 | 4.237 |
|  | History of Stroke Yes:1 No:0(1) | -.590 | .718 | .676 | 1 | .411 | .554 | .136 | 2.263 |
|  | History of Transient Ischemic Attack Yes:1 No:0(1) | -20.321 | 40192.970 | .000 | 1 | 1.000 | .000 | .000 | . |
|  | History of Peripheral Vascular Disease Yes:1 No:0(1) | .733 | .687 | 1.138 | 1 | .286 | 2.081 | .542 | 7.997 |
|  | History of Cancer Yes:1 No:0(1) | -.120 | .462 | .068 | 1 | .795 | .887 | .359 | 2.192 |
|  | History of Percutaneous Coronary Intervention Yes:1 No:0(1) | .592 | .428 | 1.910 | 1 | .167 | 1.808 | .781 | 4.186 |
|  | History of CABG Yes:1 No:0(1) | .179 | .654 | .075 | 1 | .784 | 1.196 | .332 | 4.313 |
|  | History of CKD Yes:1 No:0(1) | .403 | .497 | .657 | 1 | .418 | 1.497 | .565 | 3.968 |
|  | Taking Diuretics Yes:1 No:0(1) | -.175 | .448 | .152 | 1 | .697 | .840 | .349 | 2.021 |
|  | In patient diuretic use(1) | 1.075 | .380 | 7.983 | 1 | .005 | 2.930 | 1.390 | 6.177 |
|  | Hyponatremia present up to 7 days of hospitalization Yes:1 No:0(1) | .762 | .396 | 3.712 | 1 | .054 | 2.143 | .987 | 4.654 |
|  | Constant | -2.723 | .666 | 16.694 | 1 | <.001 | .066 |  |  |
| Step 5^a^ | SEX Female: 1 Male:0(1) | -.337 | .387 | .760 | 1 | .383 | .714 | .335 | 1.523 |
|  | Age 1<65 2:≥ 65(1) | .322 | .395 | .665 | 1 | .415 | 1.380 | .636 | 2.994 |
|  | Current Smoker Yes:1 No: 0(1) | .280 | .435 | .414 | 1 | .520 | 1.323 | .564 | 3.101 |
|  | History of HTN Yes:1 No:0(1) | .387 | .544 | .507 | 1 | .477 | 1.472 | .507 | 4.273 |
|  | History of Stroke Yes:1 No:0(1) | -.603 | .719 | .704 | 1 | .402 | .547 | .134 | 2.239 |
|  | History of Transient Ischemic Attack Yes:1 No:0(1) | -20.308 | 40192.970 | .000 | 1 | 1.000 | .000 | .000 | . |
|  | History of Peripheral Vascular Disease Yes:1 No:0(1) | .706 | .678 | 1.087 | 1 | .297 | 2.027 | .537 | 7.648 |
|  | History of Percutaneous Coronary Intervention Yes:1 No:0(1) | .601 | .427 | 1.978 | 1 | .160 | 1.823 | .789 | 4.211 |
|  | History of CABG Yes:1 No:0(1) | .187 | .653 | .082 | 1 | .775 | 1.206 | .335 | 4.335 |
|  | History of CKD Yes:1 No:0(1) | .407 | .497 | .672 | 1 | .412 | 1.503 | .567 | 3.982 |
|  | Taking Diuretics Yes:1 No:0(1) | -.179 | .447 | .160 | 1 | .689 | .836 | .348 | 2.009 |
|  | In patient diuretic use(1) | 1.083 | .379 | 8.159 | 1 | .004 | 2.955 | 1.405 | 6.213 |
|  | Hyponatremia present up to 7 days of hospitalization Yes:1 No:0(1) | .747 | .391 | 3.646 | 1 | .056 | 2.110 | .980 | 4.541 |
|  | Constant | -2.760 | .652 | 17.906 | 1 | <.001 | .063 |  |  |
| Step 6^a^ | SEX Female: 1 Male:0(1) | -.350 | .384 | .830 | 1 | .362 | .705 | .332 | 1.496 |
|  | Age 1<65 2:≥ 65(1) | .337 | .391 | .743 | 1 | .389 | 1.401 | .651 | 3.016 |
|  | Current Smoker Yes:1 No: 0(1) | .277 | .435 | .406 | 1 | .524 | 1.319 | .563 | 3.091 |
|  | History of HTN Yes:1 No:0(1) | .392 | .544 | .519 | 1 | .471 | 1.480 | .510 | 4.298 |
|  | History of Stroke Yes:1 No:0(1) | -.610 | .716 | .724 | 1 | .395 | .544 | .134 | 2.214 |
|  | History of Transient Ischemic Attack Yes:1 No:0(1) | -20.367 | 40192.970 | .000 | 1 | 1.000 | .000 | .000 | . |
|  | History of Peripheral Vascular Disease Yes:1 No:0(1) | .716 | .676 | 1.124 | 1 | .289 | 2.047 | .545 | 7.696 |
|  | History of Percutaneous Coronary Intervention Yes:1 No:0(1) | .645 | .398 | 2.620 | 1 | .106 | 1.905 | .873 | 4.158 |
|  | History of CKD Yes:1 No:0(1) | .432 | .488 | .784 | 1 | .376 | 1.541 | .592 | 4.013 |
|  | Taking Diuretics Yes:1 No:0(1) | -.172 | .446 | .148 | 1 | .700 | .842 | .352 | 2.019 |
|  | In patient diuretic use(1) | 1.082 | .379 | 8.146 | 1 | .004 | 2.949 | 1.403 | 6.198 |
|  | Hyponatremia present up to 7 days of hospitalization Yes:1 No:0(1) | .763 | .387 | 3.889 | 1 | .049 | 2.145 | 1.005 | 4.579 |
|  | Constant | -2.776 | .651 | 18.190 | 1 | <.001 | .062 |  |  |
| Step 7^a^ | SEX Female: 1 Male:0(1) | -.379 | .377 | 1.011 | 1 | .315 | .685 | .327 | 1.433 |
|  | Age 1<65 2:≥ 65(1) | .329 | .391 | .709 | 1 | .400 | 1.390 | .646 | 2.988 |
|  | Current Smoker Yes:1 No: 0(1) | .291 | .433 | .452 | 1 | .502 | 1.338 | .573 | 3.124 |
|  | History of HTN Yes:1 No:0(1) | .359 | .538 | .446 | 1 | .504 | 1.432 | .499 | 4.109 |
|  | History of Stroke Yes:1 No:0(1) | -.638 | .712 | .803 | 1 | .370 | .528 | .131 | 2.133 |
|  | History of Transient Ischemic Attack Yes:1 No:0(1) | -20.452 | 40192.970 | .000 | 1 | 1.000 | .000 | .000 | . |
|  | History of Peripheral Vascular Disease Yes:1 No:0(1) | .646 | .649 | .991 | 1 | .320 | 1.907 | .535 | 6.802 |
|  | History of Percutaneous Coronary Intervention Yes:1 No:0(1) | .648 | .398 | 2.660 | 1 | .103 | 1.913 | .877 | 4.169 |
|  | History of CKD Yes:1 No:0(1) | .447 | .487 | .844 | 1 | .358 | 1.564 | .603 | 4.057 |
|  | In patient diuretic use(1) | 1.056 | .373 | 8.010 | 1 | .005 | 2.874 | 1.383 | 5.970 |
|  | Hyponatremia present up to 7 days of hospitalization Yes:1 No:0(1) | .767 | .387 | 3.938 | 1 | .047 | 2.153 | 1.009 | 4.594 |
|  | Constant | -2.763 | .649 | 18.107 | 1 | <.001 | .063 |  |  |
| Step 8^a^ | SEX Female: 1 Male:0(1) | -.344 | .372 | .857 | 1 | .355 | .709 | .342 | 1.469 |
|  | Age 1<65 2:≥ 65(1) | .262 | .377 | .485 | 1 | .486 | 1.300 | .621 | 2.719 |
|  | History of HTN Yes:1 No:0(1) | .323 | .533 | .369 | 1 | .544 | 1.382 | .487 | 3.925 |
|  | History of Stroke Yes:1 No:0(1) | -.645 | .711 | .823 | 1 | .364 | .524 | .130 | 2.114 |
|  | History of Transient Ischemic Attack Yes:1 No:0(1) | -20.250 | 40192.970 | .000 | 1 | 1.000 | .000 | .000 | . |
|  | History of Peripheral Vascular Disease Yes:1 No:0(1) | .654 | .650 | 1.014 | 1 | .314 | 1.924 | .538 | 6.874 |
|  | History of Percutaneous Coronary Intervention Yes:1 No:0(1) | .650 | .397 | 2.680 | 1 | .102 | 1.916 | .880 | 4.175 |
|  | History of CKD Yes:1 No:0(1) | .401 | .481 | .695 | 1 | .405 | 1.494 | .581 | 3.837 |
|  | In patient diuretic use(1) | 1.040 | .372 | 7.834 | 1 | .005 | 2.830 | 1.366 | 5.863 |
|  | Hyponatremia present up to 7 days of hospitalization Yes:1 No:0(1) | .794 | .383 | 4.288 | 1 | .038 | 2.212 | 1.043 | 4.689 |
|  | Constant | -2.638 | .616 | 18.342 | 1 | <.001 | .072 |  |  |
| Step 9^a^ | SEX Female: 1 Male:0(1) | -.328 | .371 | .781 | 1 | .377 | .721 | .348 | 1.490 |
|  | Age 1<65 2:≥ 65(1) | .300 | .372 | .650 | 1 | .420 | 1.350 | .651 | 2.799 |
|  | History of Stroke Yes:1 No:0(1) | -.632 | .711 | .790 | 1 | .374 | .532 | .132 | 2.141 |
|  | History of Transient Ischemic Attack Yes:1 No:0(1) | -20.265 | 40192.970 | .000 | 1 | 1.000 | .000 | .000 | . |
|  | History of Peripheral Vascular Disease Yes:1 No:0(1) | .704 | .646 | 1.189 | 1 | .276 | 2.022 | .570 | 7.170 |
|  | History of Percutaneous Coronary Intervention Yes:1 No:0(1) | .694 | .392 | 3.128 | 1 | .077 | 2.001 | .928 | 4.318 |
|  | History of CKD Yes:1 No:0(1) | .408 | .482 | .718 | 1 | .397 | 1.504 | .585 | 3.866 |
|  | In patient diuretic use(1) | 1.054 | .371 | 8.084 | 1 | .004 | 2.869 | 1.387 | 5.932 |
|  | Hyponatremia present up to 7 days of hospitalization Yes:1 No:0(1) | .791 | .383 | 4.263 | 1 | .039 | 2.205 | 1.041 | 4.672 |
|  | Constant | -2.416 | .483 | 24.993 | 1 | <.001 | .089 |  |  |
| Step 10^a^ | SEX Female: 1 Male:0(1) | -.356 | .369 | .930 | 1 | .335 | .701 | .340 | 1.444 |
|  | Age 1<65 2:≥ 65(1) | .326 | .370 | .775 | 1 | .379 | 1.385 | .670 | 2.863 |
|  | History of Stroke Yes:1 No:0(1) | -.629 | .711 | .783 | 1 | .376 | .533 | .132 | 2.147 |
|  | History of Peripheral Vascular Disease Yes:1 No:0(1) | .639 | .640 | .995 | 1 | .319 | 1.894 | .540 | 6.648 |
|  | History of Percutaneous Coronary Intervention Yes:1 No:0(1) | .676 | .392 | 2.968 | 1 | .085 | 1.966 | .911 | 4.243 |
|  | History of CKD Yes:1 No:0(1) | .372 | .480 | .600 | 1 | .439 | 1.450 | .566 | 3.715 |
|  | In patient diuretic use(1) | 1.068 | .371 | 8.311 | 1 | .004 | 2.910 | 1.408 | 6.017 |
|  | Hyponatremia present up to 7 days of hospitalization Yes:1 No:0(1) | .808 | .383 | 4.450 | 1 | .035 | 2.244 | 1.059 | 4.754 |
|  | Constant | -2.428 | .484 | 25.122 | 1 | <.001 | .088 |  |  |
| Step 11^a^ | SEX Female: 1 Male:0(1) | -.337 | .367 | .842 | 1 | .359 | .714 | .348 | 1.466 |
|  | Age 1<65 2:≥ 65(1) | .377 | .363 | 1.076 | 1 | .299 | 1.458 | .715 | 2.971 |
|  | History of Stroke Yes:1 No:0(1) | -.607 | .705 | .742 | 1 | .389 | .545 | .137 | 2.170 |
|  | History of Peripheral Vascular Disease Yes:1 No:0(1) | .751 | .626 | 1.441 | 1 | .230 | 2.119 | .622 | 7.226 |
|  | History of Percutaneous Coronary Intervention Yes:1 No:0(1) | .672 | .393 | 2.920 | 1 | .087 | 1.957 | .906 | 4.229 |
|  | In patient diuretic use(1) | 1.073 | .369 | 8.434 | 1 | .004 | 2.923 | 1.417 | 6.029 |
|  | Hyponatremia present up to 7 days of hospitalization Yes:1 No:0(1) | .791 | .381 | 4.313 | 1 | .038 | 2.206 | 1.045 | 4.655 |
|  | Constant | -2.399 | .481 | 24.879 | 1 | <.001 | .091 |  |  |
| Step 12^a^ | SEX Female: 1 Male:0(1) | -.351 | .366 | .919 | 1 | .338 | .704 | .344 | 1.442 |
|  | Age 1<65 2:≥ 65(1) | .348 | .361 | .929 | 1 | .335 | 1.416 | .698 | 2.870 |
|  | History of Peripheral Vascular Disease Yes:1 No:0(1) | .803 | .623 | 1.662 | 1 | .197 | 2.232 | .658 | 7.569 |
|  | History of Percutaneous Coronary Intervention Yes:1 No:0(1) | .647 | .391 | 2.737 | 1 | .098 | 1.910 | .887 | 4.110 |
|  | In patient diuretic use(1) | 1.057 | .368 | 8.246 | 1 | .004 | 2.876 | 1.398 | 5.916 |
|  | Hyponatremia present up to 7 days of hospitalization Yes:1 No:0(1) | .795 | .380 | 4.382 | 1 | .036 | 2.214 | 1.052 | 4.659 |
|  | Constant | -2.416 | .481 | 25.223 | 1 | <.001 | .089 |  |  |
| Step 13^a^ | Age 1<65 2:≥ 65(1) | .297 | .355 | .697 | 1 | .404 | 1.345 | .670 | 2.700 |
|  | History of Peripheral Vascular Disease Yes:1 No:0(1) | .860 | .622 | 1.912 | 1 | .167 | 2.363 | .698 | 7.993 |
|  | History of Percutaneous Coronary Intervention Yes:1 No:0(1) | .666 | .390 | 2.912 | 1 | .088 | 1.946 | .906 | 4.179 |
|  | In patient diuretic use(1) | 1.041 | .366 | 8.075 | 1 | .004 | 2.832 | 1.381 | 5.805 |
|  | Hyponatremia present up to 7 days of hospitalization Yes:1 No:0(1) | .827 | .379 | 4.770 | 1 | .029 | 2.286 | 1.088 | 4.801 |
|  | Constant | -2.565 | .462 | 30.778 | 1 | <.001 | .077 |  |  |
| Step 14^a^ | History of Peripheral Vascular Disease Yes:1 No:0(1) | .819 | .622 | 1.731 | 1 | .188 | 2.268 | .670 | 7.681 |
|  | History of Percutaneous Coronary Intervention Yes:1 No:0(1) | .677 | .388 | 3.038 | 1 | .081 | 1.968 | .919 | 4.212 |
|  | In patient diuretic use(1) | 1.066 | .365 | 8.553 | 1 | .003 | 2.905 | 1.422 | 5.936 |
|  | Hyponatremia present up to 7 days of hospitalization Yes:1 No:0(1) | .793 | .375 | 4.464 | 1 | .035 | 2.210 | 1.059 | 4.612 |
|  | Constant | -2.411 | .417 | 33.422 | 1 | <.001 | .090 |  |  |
| Step 15^a^ | History of Percutaneous Coronary Intervention Yes:1 No:0(1) | .790 | .376 | 4.411 | 1 | .036 | 2.204 | 1.054 | 4.607 |
|  | In patient diuretic use(1) | 1.006 | .358 | 7.906 | 1 | .005 | 2.733 | 1.356 | 5.509 |
|  | Hyponatremia present up to 7 days of hospitalization Yes:1 No:0(1) | .759 | .372 | 4.164 | 1 | .041 | 2.137 | 1.030 | 4.432 |
|  | Constant | -2.314 | .405 | 32.614 | 1 | <.001 | .099 |  |  |
| a. Variable(s) entered on step 1: SEX Female: 1 Male:0, Age 1<65 2:≥ 65, Current Smoker Yes:1 No: 0, History of HTN Yes:1 No:0, History of Diabetes Yes:1 No:0, History of Stroke Yes:1 No:0, History of Transient Ischemic Attack Yes:1 No:0, History of Peripheral Vascular Disease Yes:1 No:0, History of Cancer Yes:1 No:0, History of Myocardial Infarction Yes:1 No:0, History of Percutaneous Coronary Intervention Yes:1 No:0, History of CABG Yes:1 No:0, History of CKD Yes:1 No:0, Taking ACEi, ARNi, or ARB Yes:1 No:0, Taking Diuretics Yes:1 No:0, In patient diuretic use, Hyponatremia present up to 7 days of hospitalization Yes:1 No:0. | | | | | | | | | |

**Dependent variable: EF<50%**

**Independent variable: Hyponatremia up to 7 days of admission**

| **Omnibus Tests of Model Coefficients** | | | | |
| --- | --- | --- | --- | --- |
|  | | Chi-square | df | Sig. |
| Step 1 | Step | 91.209 | 19 | <.001 |
|  | Block | 91.209 | 19 | <.001 |
|  | Model | 91.209 | 19 | <.001 |
| Step 2^a^ | Step | -.027 | 1 | .869 |
|  | Block | 91.182 | 18 | <.001 |
|  | Model | 91.182 | 18 | <.001 |
| Step 3^a^ | Step | -.041 | 1 | .840 |
|  | Block | 91.141 | 17 | <.001 |
|  | Model | 91.141 | 17 | <.001 |
| Step 4^a^ | Step | -.181 | 1 | .670 |
|  | Block | 90.960 | 16 | <.001 |
|  | Model | 90.960 | 16 | <.001 |
| Step 5^a^ | Step | -.226 | 1 | .635 |
|  | Block | 90.734 | 15 | <.001 |
|  | Model | 90.734 | 15 | <.001 |
| Step 6^a^ | Step | -.280 | 1 | .597 |
|  | Block | 90.454 | 14 | <.001 |
|  | Model | 90.454 | 14 | <.001 |
| Step 7^a^ | Step | -.295 | 1 | .587 |
|  | Block | 90.159 | 13 | <.001 |
|  | Model | 90.159 | 13 | <.001 |
| Step 8^a^ | Step | -.643 | 1 | .423 |
|  | Block | 89.516 | 12 | <.001 |
|  | Model | 89.516 | 12 | <.001 |
| Step 9^a^ | Step | -.871 | 1 | .351 |
|  | Block | 88.645 | 11 | <.001 |
|  | Model | 88.645 | 11 | <.001 |
| Step 10^a^ | Step | -.954 | 1 | .329 |
|  | Block | 87.691 | 10 | <.001 |
|  | Model | 87.691 | 10 | <.001 |
| Step 11^a^ | Step | -1.267 | 1 | .260 |
|  | Block | 86.423 | 9 | <.001 |
|  | Model | 86.423 | 9 | <.001 |
| Step 12^a^ | Step | -1.625 | 1 | .202 |
|  | Block | 84.798 | 8 | <.001 |
|  | Model | 84.798 | 8 | <.001 |
| a. A negative Chi-squares value indicates that the Chi-squares value has decreased from the previous step. | | | | |

| **Hosmer and Lemeshow Test** | | | |
| --- | --- | --- | --- |
| Step | Chi-square | df | Sig. |
| 1 | 2.016 | 8 | .981 |
| 2 | 3.154 | 8 | .924 |
| 3 | 2.204 | 8 | .974 |
| 4 | 8.343 | 8 | .401 |
| 5 | 7.941 | 8 | .439 |
| 6 | 9.033 | 8 | .339 |
| 7 | 14.753 | 8 | .064 |
| 8 | 11.489 | 8 | .175 |
| 9 | 12.095 | 8 | .147 |
| 10 | 14.142 | 8 | .078 |
| 11 | 15.273 | 8 | .054 |
| 12 | 9.790 | 8 | .280 |

| **Classification Table**^a^ | | | | | |
| --- | --- | --- | --- | --- | --- |
|  | Observed | | Predicted | | |
|  |  |  | Left ventricular ejection fraction <50% Yes:1 No:0 | | Percentage Correct |
|  |  |  | ≥50 | <50 |  |
| Step 1 | Left ventricular ejection fraction <50% Yes:1 No:0 | ≥50 | 106 | 21 | 83.5 |
|  |  | <50 | 27 | 67 | 71.3 |
|  | Overall Percentage | |  |  | 78.3 |
| Step 2 | Left ventricular ejection fraction <50% Yes:1 No:0 | ≥50 | 106 | 21 | 83.5 |
|  |  | <50 | 27 | 67 | 71.3 |
|  | Overall Percentage | |  |  | 78.3 |
| Step 3 | Left ventricular ejection fraction <50% Yes:1 No:0 | ≥50 | 107 | 20 | 84.3 |
|  |  | <50 | 26 | 68 | 72.3 |
|  | Overall Percentage | |  |  | 79.2 |
| Step 4 | Left ventricular ejection fraction <50% Yes:1 No:0 | ≥50 | 107 | 20 | 84.3 |
|  |  | <50 | 26 | 68 | 72.3 |
|  | Overall Percentage | |  |  | 79.2 |
| Step 5 | Left ventricular ejection fraction <50% Yes:1 No:0 | ≥50 | 107 | 20 | 84.3 |
|  |  | <50 | 26 | 68 | 72.3 |
|  | Overall Percentage | |  |  | 79.2 |
| Step 6 | Left ventricular ejection fraction <50% Yes:1 No:0 | ≥50 | 106 | 21 | 83.5 |
|  |  | <50 | 26 | 68 | 72.3 |
|  | Overall Percentage | |  |  | 78.7 |
| Step 7 | Left ventricular ejection fraction <50% Yes:1 No:0 | ≥50 | 107 | 20 | 84.3 |
|  |  | <50 | 26 | 68 | 72.3 |
|  | Overall Percentage | |  |  | 79.2 |
| Step 8 | Left ventricular ejection fraction <50% Yes:1 No:0 | ≥50 | 106 | 21 | 83.5 |
|  |  | <50 | 26 | 68 | 72.3 |
|  | Overall Percentage | |  |  | 78.7 |
| Step 9 | Left ventricular ejection fraction <50% Yes:1 No:0 | ≥50 | 107 | 20 | 84.3 |
|  |  | <50 | 26 | 68 | 72.3 |
|  | Overall Percentage | |  |  | 79.2 |
| Step 10 | Left ventricular ejection fraction <50% Yes:1 No:0 | ≥50 | 107 | 20 | 84.3 |
|  |  | <50 | 28 | 66 | 70.2 |
|  | Overall Percentage | |  |  | 78.3 |
| Step 11 | Left ventricular ejection fraction <50% Yes:1 No:0 | ≥50 | 107 | 20 | 84.3 |
|  |  | <50 | 26 | 68 | 72.3 |
|  | Overall Percentage | |  |  | 79.2 |
| Step 12 | Left ventricular ejection fraction <50% Yes:1 No:0 | ≥50 | 106 | 21 | 83.5 |
|  |  | <50 | 26 | 68 | 72.3 |
|  | Overall Percentage | |  |  | 78.7 |
| a. The cut value is .500 | | | | | |

| **Variables in the Equation** | | | | | | | | | |
| --- | --- | --- | --- | --- | --- | --- | --- | --- | --- |
|  | | B | S.E. | Wald | df | Sig. | Exp(B) | 95% C.I.for EXP(B) | |
|  |  |  |  |  |  |  |  | Lower | Upper |
| Step 1^a^ | SEX Female: 1 Male:0(1) | -.941 | .378 | 6.215 | 1 | .013 | .390 | .186 | .818 |
|  | Age 1<65 2:≥ 65(1) | .385 | .379 | 1.029 | 1 | .310 | 1.469 | .699 | 3.090 |
|  | Current Smoker Yes:1 No: 0(1) | .635 | .440 | 2.087 | 1 | .149 | 1.888 | .797 | 4.471 |
|  | History of HTN Yes:1 No:0(1) | -1.422 | .482 | 8.705 | 1 | .003 | .241 | .094 | .620 |
|  | History of Diabetes Yes:1 No:0(1) | .208 | .382 | .298 | 1 | .585 | 1.232 | .583 | 2.602 |
|  | History of Stroke Yes:1 No:0(1) | -1.975 | .859 | 5.279 | 1 | .022 | .139 | .026 | .748 |
|  | History of Transient Ischemic Attack Yes:1 No:0(1) | -18.521 | 28243.718 | .000 | 1 | .999 | .000 | .000 | . |
|  | History of Peripheral Vascular Disease Yes:1 No:0(1) | .104 | .631 | .027 | 1 | .869 | 1.110 | .322 | 3.824 |
|  | History of Cancer Yes:1 No:0(1) | .369 | .437 | .712 | 1 | .399 | 1.446 | .614 | 3.408 |
|  | History of Myocardial Infarction Yes:1 No:0(1) | .219 | .506 | .188 | 1 | .664 | 1.245 | .462 | 3.354 |
|  | History of Percutaneous Coronary Intervention Yes:1 No:0(1) | .399 | .480 | .693 | 1 | .405 | 1.491 | .582 | 3.816 |
|  | History of CABG Yes:1 No:0(1) | .522 | .608 | .736 | 1 | .391 | 1.685 | .512 | 5.547 |
|  | History of CKD Yes:1 No:0(1) | -.228 | .488 | .218 | 1 | .640 | .796 | .306 | 2.073 |
|  | Taking ACEi, ARNi, or ARB Yes:1 No:0(1) | .083 | .394 | .045 | 1 | .833 | 1.087 | .502 | 2.351 |
|  | Taking Diuretics Yes:1 No:0(1) | -.434 | .428 | 1.030 | 1 | .310 | .648 | .280 | 1.498 |
|  | Heart failure diagnosis (0=No HF, 1= History of HF, 2=New HF) |  |  | 29.903 | 2 | <.001 |  |  |  |
|  | Heart failure diagnosis (0=No HF, 1= History of HF, 2=New HF)(1) | .747 | .495 | 2.273 | 1 | .132 | 2.110 | .799 | 5.572 |
|  | Heart failure diagnosis (0=No HF, 1= History of HF, 2=New HF)(2) | 2.628 | .481 | 29.898 | 1 | <.001 | 13.848 | 5.398 | 35.522 |
|  | In patient diuretic use(1) | 1.162 | .381 | 9.303 | 1 | .002 | 3.195 | 1.515 | 6.741 |
|  | Hyponatremia present up to 7 days of hospitalization Yes:1 No:0(1) | .692 | .385 | 3.223 | 1 | .073 | 1.997 | .939 | 4.251 |
|  | Constant | -1.102 | .531 | 4.298 | 1 | .038 | .332 |  |  |
| Step 2^a^ | SEX Female: 1 Male:0(1) | -.946 | .376 | 6.320 | 1 | .012 | .388 | .186 | .812 |
|  | Age 1<65 2:≥ 65(1) | .378 | .377 | 1.006 | 1 | .316 | 1.460 | .697 | 3.057 |
|  | Current Smoker Yes:1 No: 0(1) | .642 | .438 | 2.141 | 1 | .143 | 1.900 | .804 | 4.486 |
|  | History of HTN Yes:1 No:0(1) | -1.418 | .481 | 8.682 | 1 | .003 | .242 | .094 | .622 |
|  | History of Diabetes Yes:1 No:0(1) | .216 | .379 | .327 | 1 | .568 | 1.241 | .591 | 2.607 |
|  | History of Stroke Yes:1 No:0(1) | -1.976 | .858 | 5.308 | 1 | .021 | .139 | .026 | .744 |
|  | History of Transient Ischemic Attack Yes:1 No:0(1) | -18.441 | 28247.515 | .000 | 1 | .999 | .000 | .000 | . |
|  | History of Cancer Yes:1 No:0(1) | .376 | .435 | .750 | 1 | .387 | 1.457 | .622 | 3.414 |
|  | History of Myocardial Infarction Yes:1 No:0(1) | .217 | .505 | .185 | 1 | .667 | 1.243 | .462 | 3.344 |
|  | History of Percutaneous Coronary Intervention Yes:1 No:0(1) | .409 | .476 | .738 | 1 | .390 | 1.505 | .592 | 3.828 |
|  | History of CABG Yes:1 No:0(1) | .541 | .597 | .819 | 1 | .366 | 1.717 | .532 | 5.537 |
|  | History of CKD Yes:1 No:0(1) | -.232 | .488 | .226 | 1 | .634 | .793 | .305 | 2.063 |
|  | Taking ACEi, ARNi, or ARB Yes:1 No:0(1) | .079 | .393 | .041 | 1 | .840 | 1.082 | .501 | 2.339 |
|  | Taking Diuretics Yes:1 No:0(1) | -.430 | .427 | 1.016 | 1 | .314 | .650 | .282 | 1.501 |
|  | Heart failure diagnosis (0=No HF, 1= History of HF, 2=New HF) |  |  | 30.208 | 2 | <.001 |  |  |  |
|  | Heart failure diagnosis (0=No HF, 1= History of HF, 2=New HF)(1) | .752 | .495 | 2.311 | 1 | .128 | 2.121 | .805 | 5.594 |
|  | Heart failure diagnosis (0=No HF, 1= History of HF, 2=New HF)(2) | 2.635 | .479 | 30.206 | 1 | <.001 | 13.938 | 5.447 | 35.664 |
|  | In patient diuretic use(1) | 1.159 | .381 | 9.285 | 1 | .002 | 3.188 | 1.512 | 6.721 |
|  | Hyponatremia present up to 7 days of hospitalization Yes:1 No:0(1) | .686 | .384 | 3.194 | 1 | .074 | 1.986 | .936 | 4.215 |
|  | Constant | -1.097 | .530 | 4.280 | 1 | .039 | .334 |  |  |
| Step 3^a^ | SEX Female: 1 Male:0(1) | -.939 | .374 | 6.293 | 1 | .012 | .391 | .188 | .814 |
|  | Age 1<65 2:≥ 65(1) | .385 | .376 | 1.051 | 1 | .305 | 1.470 | .704 | 3.069 |
|  | Current Smoker Yes:1 No: 0(1) | .636 | .437 | 2.114 | 1 | .146 | 1.888 | .802 | 4.449 |
|  | History of HTN Yes:1 No:0(1) | -1.401 | .473 | 8.767 | 1 | .003 | .246 | .097 | .623 |
|  | History of Diabetes Yes:1 No:0(1) | .222 | .377 | .346 | 1 | .556 | 1.249 | .596 | 2.616 |
|  | History of Stroke Yes:1 No:0(1) | -1.975 | .860 | 5.275 | 1 | .022 | .139 | .026 | .749 |
|  | History of Transient Ischemic Attack Yes:1 No:0(1) | -18.473 | 28239.053 | .000 | 1 | .999 | .000 | .000 | . |
|  | History of Cancer Yes:1 No:0(1) | .365 | .431 | .717 | 1 | .397 | 1.440 | .619 | 3.350 |
|  | History of Myocardial Infarction Yes:1 No:0(1) | .215 | .505 | .182 | 1 | .670 | 1.240 | .461 | 3.335 |
|  | History of Percutaneous Coronary Intervention Yes:1 No:0(1) | .414 | .475 | .759 | 1 | .384 | 1.513 | .596 | 3.842 |
|  | History of CABG Yes:1 No:0(1) | .532 | .596 | .799 | 1 | .371 | 1.703 | .530 | 5.471 |
|  | History of CKD Yes:1 No:0(1) | -.248 | .481 | .266 | 1 | .606 | .780 | .304 | 2.004 |
|  | Taking Diuretics Yes:1 No:0(1) | -.418 | .422 | .981 | 1 | .322 | .658 | .288 | 1.506 |
|  | Heart failure diagnosis (0=No HF, 1= History of HF, 2=New HF) |  |  | 30.257 | 2 | <.001 |  |  |  |
|  | Heart failure diagnosis (0=No HF, 1= History of HF, 2=New HF)(1) | .761 | .493 | 2.384 | 1 | .123 | 2.141 | .815 | 5.626 |
|  | Heart failure diagnosis (0=No HF, 1= History of HF, 2=New HF)(2) | 2.636 | .479 | 30.256 | 1 | <.001 | 13.959 | 5.457 | 35.711 |
|  | In patient diuretic use(1) | 1.161 | .380 | 9.346 | 1 | .002 | 3.194 | 1.517 | 6.723 |
|  | Hyponatremia present up to 7 days of hospitalization Yes:1 No:0(1) | .690 | .384 | 3.232 | 1 | .072 | 1.993 | .940 | 4.229 |
|  | Constant | -1.094 | .531 | 4.251 | 1 | .039 | .335 |  |  |
| Step 4^a^ | SEX Female: 1 Male:0(1) | -.962 | .371 | 6.720 | 1 | .010 | .382 | .185 | .791 |
|  | Age 1<65 2:≥ 65(1) | .382 | .375 | 1.038 | 1 | .308 | 1.465 | .702 | 3.057 |
|  | Current Smoker Yes:1 No: 0(1) | .666 | .430 | 2.396 | 1 | .122 | 1.947 | .838 | 4.524 |
|  | History of HTN Yes:1 No:0(1) | -1.418 | .471 | 9.053 | 1 | .003 | .242 | .096 | .610 |
|  | History of Diabetes Yes:1 No:0(1) | .237 | .376 | .399 | 1 | .528 | 1.268 | .607 | 2.649 |
|  | History of Stroke Yes:1 No:0(1) | -2.042 | .851 | 5.763 | 1 | .016 | .130 | .024 | .687 |
|  | History of Transient Ischemic Attack Yes:1 No:0(1) | -18.384 | 28239.126 | .000 | 1 | .999 | .000 | .000 | . |
|  | History of Cancer Yes:1 No:0(1) | .373 | .430 | .754 | 1 | .385 | 1.453 | .625 | 3.376 |
|  | History of Percutaneous Coronary Intervention Yes:1 No:0(1) | .502 | .428 | 1.372 | 1 | .241 | 1.651 | .713 | 3.822 |
|  | History of CABG Yes:1 No:0(1) | .575 | .587 | .959 | 1 | .327 | 1.777 | .562 | 5.616 |
|  | History of CKD Yes:1 No:0(1) | -.230 | .478 | .231 | 1 | .631 | .795 | .311 | 2.030 |
|  | Taking Diuretics Yes:1 No:0(1) | -.403 | .420 | .919 | 1 | .338 | .669 | .294 | 1.523 |
|  | Heart failure diagnosis (0=No HF, 1= History of HF, 2=New HF) |  |  | 30.378 | 2 | <.001 |  |  |  |
|  | Heart failure diagnosis (0=No HF, 1= History of HF, 2=New HF)(1) | .794 | .486 | 2.676 | 1 | .102 | 2.213 | .854 | 5.732 |
|  | Heart failure diagnosis (0=No HF, 1= History of HF, 2=New HF)(2) | 2.645 | .480 | 30.371 | 1 | <.001 | 14.087 | 5.499 | 36.092 |
|  | In patient diuretic use(1) | 1.167 | .379 | 9.459 | 1 | .002 | 3.213 | 1.527 | 6.759 |
|  | Hyponatremia present up to 7 days of hospitalization Yes:1 No:0(1) | .664 | .377 | 3.093 | 1 | .079 | 1.942 | .927 | 4.070 |
|  | Constant | -1.065 | .524 | 4.127 | 1 | .042 | .345 |  |  |
| Step 5^a^ | SEX Female: 1 Male:0(1) | -.966 | .371 | 6.774 | 1 | .009 | .381 | .184 | .788 |
|  | Age 1<65 2:≥ 65(1) | .394 | .374 | 1.108 | 1 | .292 | 1.483 | .712 | 3.088 |
|  | Current Smoker Yes:1 No: 0(1) | .661 | .429 | 2.373 | 1 | .123 | 1.938 | .835 | 4.495 |
|  | History of HTN Yes:1 No:0(1) | -1.423 | .472 | 9.099 | 1 | .003 | .241 | .096 | .607 |
|  | History of Diabetes Yes:1 No:0(1) | .232 | .376 | .381 | 1 | .537 | 1.261 | .603 | 2.637 |
|  | History of Stroke Yes:1 No:0(1) | -2.076 | .843 | 6.062 | 1 | .014 | .125 | .024 | .655 |
|  | History of Cancer Yes:1 No:0(1) | .377 | .431 | .768 | 1 | .381 | 1.459 | .627 | 3.392 |
|  | History of Percutaneous Coronary Intervention Yes:1 No:0(1) | .491 | .428 | 1.314 | 1 | .252 | 1.633 | .706 | 3.778 |
|  | History of CABG Yes:1 No:0(1) | .595 | .585 | 1.034 | 1 | .309 | 1.813 | .576 | 5.711 |
|  | History of CKD Yes:1 No:0(1) | -.251 | .475 | .279 | 1 | .598 | .778 | .306 | 1.976 |
|  | Taking Diuretics Yes:1 No:0(1) | -.415 | .419 | .981 | 1 | .322 | .660 | .290 | 1.501 |
|  | Heart failure diagnosis (0=No HF, 1= History of HF, 2=New HF) |  |  | 30.579 | 2 | <.001 |  |  |  |
|  | Heart failure diagnosis (0=No HF, 1= History of HF, 2=New HF)(1) | .795 | .485 | 2.685 | 1 | .101 | 2.215 | .856 | 5.735 |
|  | Heart failure diagnosis (0=No HF, 1= History of HF, 2=New HF)(2) | 2.655 | .480 | 30.573 | 1 | <.001 | 14.226 | 5.551 | 36.461 |
|  | In patient diuretic use(1) | 1.180 | .378 | 9.719 | 1 | .002 | 3.254 | 1.550 | 6.833 |
|  | Hyponatremia present up to 7 days of hospitalization Yes:1 No:0(1) | .668 | .378 | 3.132 | 1 | .077 | 1.951 | .931 | 4.089 |
|  | Constant | -1.069 | .525 | 4.147 | 1 | .042 | .343 |  |  |
| Step 6^a^ | SEX Female: 1 Male:0(1) | -.967 | .371 | 6.779 | 1 | .009 | .380 | .184 | .787 |
|  | Age 1<65 2:≥ 65(1) | .377 | .372 | 1.024 | 1 | .312 | 1.457 | .703 | 3.022 |
|  | Current Smoker Yes:1 No: 0(1) | .683 | .428 | 2.554 | 1 | .110 | 1.981 | .857 | 4.579 |
|  | History of HTN Yes:1 No:0(1) | -1.436 | .471 | 9.295 | 1 | .002 | .238 | .094 | .599 |
|  | History of Diabetes Yes:1 No:0(1) | .202 | .372 | .295 | 1 | .587 | 1.224 | .591 | 2.537 |
|  | History of Stroke Yes:1 No:0(1) | -2.078 | .837 | 6.159 | 1 | .013 | .125 | .024 | .646 |
|  | History of Cancer Yes:1 No:0(1) | .377 | .430 | .770 | 1 | .380 | 1.458 | .628 | 3.388 |
|  | History of Percutaneous Coronary Intervention Yes:1 No:0(1) | .517 | .425 | 1.479 | 1 | .224 | 1.676 | .729 | 3.855 |
|  | History of CABG Yes:1 No:0(1) | .532 | .570 | .869 | 1 | .351 | 1.702 | .557 | 5.202 |
|  | Taking Diuretics Yes:1 No:0(1) | -.432 | .417 | 1.071 | 1 | .301 | .649 | .287 | 1.471 |
|  | Heart failure diagnosis (0=No HF, 1= History of HF, 2=New HF) |  |  | 30.466 | 2 | <.001 |  |  |  |
|  | Heart failure diagnosis (0=No HF, 1= History of HF, 2=New HF)(1) | .746 | .474 | 2.479 | 1 | .115 | 2.108 | .833 | 5.333 |
|  | Heart failure diagnosis (0=No HF, 1= History of HF, 2=New HF)(2) | 2.637 | .478 | 30.461 | 1 | <.001 | 13.978 | 5.479 | 35.662 |
|  | In patient diuretic use(1) | 1.179 | .378 | 9.731 | 1 | .002 | 3.250 | 1.550 | 6.816 |
|  | Hyponatremia present up to 7 days of hospitalization Yes:1 No:0(1) | .666 | .378 | 3.110 | 1 | .078 | 1.947 | .928 | 4.084 |
|  | Constant | -1.073 | .525 | 4.174 | 1 | .041 | .342 |  |  |
| Step 7^a^ | SEX Female: 1 Male:0(1) | -.945 | .369 | 6.560 | 1 | .010 | .389 | .189 | .801 |
|  | Age 1<65 2:≥ 65(1) | .379 | .372 | 1.038 | 1 | .308 | 1.460 | .705 | 3.025 |
|  | Current Smoker Yes:1 No: 0(1) | .651 | .422 | 2.375 | 1 | .123 | 1.917 | .838 | 4.388 |
|  | History of HTN Yes:1 No:0(1) | -1.408 | .467 | 9.084 | 1 | .003 | .245 | .098 | .611 |
|  | History of Stroke Yes:1 No:0(1) | -2.097 | .843 | 6.184 | 1 | .013 | .123 | .024 | .641 |
|  | History of Cancer Yes:1 No:0(1) | .342 | .426 | .643 | 1 | .423 | 1.408 | .610 | 3.247 |
|  | History of Percutaneous Coronary Intervention Yes:1 No:0(1) | .534 | .424 | 1.586 | 1 | .208 | 1.707 | .743 | 3.921 |
|  | History of CABG Yes:1 No:0(1) | .561 | .567 | .979 | 1 | .322 | 1.753 | .577 | 5.331 |
|  | Taking Diuretics Yes:1 No:0(1) | -.417 | .416 | 1.004 | 1 | .316 | .659 | .292 | 1.490 |
|  | Heart failure diagnosis (0=No HF, 1= History of HF, 2=New HF) |  |  | 30.514 | 2 | <.001 |  |  |  |
|  | Heart failure diagnosis (0=No HF, 1= History of HF, 2=New HF)(1) | .762 | .473 | 2.596 | 1 | .107 | 2.142 | .848 | 5.412 |
|  | Heart failure diagnosis (0=No HF, 1= History of HF, 2=New HF)(2) | 2.635 | .477 | 30.505 | 1 | <.001 | 13.943 | 5.474 | 35.519 |
|  | In patient diuretic use(1) | 1.192 | .377 | 9.982 | 1 | .002 | 3.294 | 1.572 | 6.899 |
|  | Hyponatremia present up to 7 days of hospitalization Yes:1 No:0(1) | .664 | .377 | 3.099 | 1 | .078 | 1.943 | .927 | 4.072 |
|  | Constant | -1.026 | .516 | 3.951 | 1 | .047 | .359 |  |  |
| Step 8^a^ | SEX Female: 1 Male:0(1) | -.910 | .366 | 6.193 | 1 | .013 | .403 | .197 | .824 |
|  | Age 1<65 2:≥ 65(1) | .408 | .368 | 1.229 | 1 | .268 | 1.504 | .731 | 3.097 |
|  | Current Smoker Yes:1 No: 0(1) | .565 | .408 | 1.918 | 1 | .166 | 1.760 | .791 | 3.915 |
|  | History of HTN Yes:1 No:0(1) | -1.434 | .466 | 9.483 | 1 | .002 | .238 | .096 | .594 |
|  | History of Stroke Yes:1 No:0(1) | -2.066 | .851 | 5.893 | 1 | .015 | .127 | .024 | .672 |
|  | History of Percutaneous Coronary Intervention Yes:1 No:0(1) | .546 | .423 | 1.666 | 1 | .197 | 1.726 | .754 | 3.954 |
|  | History of CABG Yes:1 No:0(1) | .527 | .566 | .866 | 1 | .352 | 1.693 | .559 | 5.133 |
|  | Taking Diuretics Yes:1 No:0(1) | -.401 | .414 | .938 | 1 | .333 | .669 | .297 | 1.508 |
|  | Heart failure diagnosis (0=No HF, 1= History of HF, 2=New HF) |  |  | 30.339 | 2 | <.001 |  |  |  |
|  | Heart failure diagnosis (0=No HF, 1= History of HF, 2=New HF)(1) | .757 | .473 | 2.559 | 1 | .110 | 2.133 | .843 | 5.394 |
|  | Heart failure diagnosis (0=No HF, 1= History of HF, 2=New HF)(2) | 2.614 | .475 | 30.332 | 1 | <.001 | 13.658 | 5.387 | 34.630 |
|  | In patient diuretic use(1) | 1.170 | .374 | 9.803 | 1 | .002 | 3.223 | 1.549 | 6.706 |
|  | Hyponatremia present up to 7 days of hospitalization Yes:1 No:0(1) | .699 | .375 | 3.479 | 1 | .062 | 2.011 | .965 | 4.190 |
|  | Constant | -.944 | .502 | 3.529 | 1 | .060 | .389 |  |  |
| Step 9^a^ | SEX Female: 1 Male:0(1) | -.944 | .363 | 6.752 | 1 | .009 | .389 | .191 | .793 |
|  | Age 1<65 2:≥ 65(1) | .410 | .366 | 1.254 | 1 | .263 | 1.507 | .735 | 3.091 |
|  | Current Smoker Yes:1 No: 0(1) | .545 | .408 | 1.784 | 1 | .182 | 1.725 | .775 | 3.837 |
|  | History of HTN Yes:1 No:0(1) | -1.400 | .465 | 9.058 | 1 | .003 | .246 | .099 | .614 |
|  | History of Stroke Yes:1 No:0(1) | -2.018 | .844 | 5.713 | 1 | .017 | .133 | .025 | .695 |
|  | History of Percutaneous Coronary Intervention Yes:1 No:0(1) | .656 | .404 | 2.637 | 1 | .104 | 1.927 | .873 | 4.255 |
|  | Taking Diuretics Yes:1 No:0(1) | -.402 | .414 | .943 | 1 | .332 | .669 | .297 | 1.506 |
|  | Heart failure diagnosis (0=No HF, 1= History of HF, 2=New HF) |  |  | 30.552 | 2 | <.001 |  |  |  |
|  | Heart failure diagnosis (0=No HF, 1= History of HF, 2=New HF)(1) | .857 | .460 | 3.471 | 1 | .062 | 2.356 | .956 | 5.801 |
|  | Heart failure diagnosis (0=No HF, 1= History of HF, 2=New HF)(2) | 2.608 | .472 | 30.498 | 1 | <.001 | 13.569 | 5.378 | 34.236 |
|  | In patient diuretic use(1) | 1.180 | .375 | 9.908 | 1 | .002 | 3.256 | 1.561 | 6.790 |
|  | Hyponatremia present up to 7 days of hospitalization Yes:1 No:0(1) | .734 | .372 | 3.891 | 1 | .049 | 2.084 | 1.005 | 4.323 |
|  | Constant | -.967 | .503 | 3.697 | 1 | .055 | .380 |  |  |
| Step 10^a^ | SEX Female: 1 Male:0(1) | -.979 | .361 | 7.366 | 1 | .007 | .376 | .185 | .762 |
|  | Age 1<65 2:≥ 65(1) | .411 | .366 | 1.258 | 1 | .262 | 1.508 | .736 | 3.091 |
|  | Current Smoker Yes:1 No: 0(1) | .581 | .406 | 2.045 | 1 | .153 | 1.787 | .806 | 3.960 |
|  | History of HTN Yes:1 No:0(1) | -1.457 | .463 | 9.899 | 1 | .002 | .233 | .094 | .577 |
|  | History of Stroke Yes:1 No:0(1) | -2.043 | .831 | 6.051 | 1 | .014 | .130 | .025 | .660 |
|  | History of Percutaneous Coronary Intervention Yes:1 No:0(1) | .655 | .402 | 2.654 | 1 | .103 | 1.924 | .875 | 4.229 |
|  | Heart failure diagnosis (0=No HF, 1= History of HF, 2=New HF) |  |  | 30.421 | 2 | <.001 |  |  |  |
|  | Heart failure diagnosis (0=No HF, 1= History of HF, 2=New HF)(1) | .749 | .444 | 2.851 | 1 | .091 | 2.116 | .887 | 5.049 |
|  | Heart failure diagnosis (0=No HF, 1= History of HF, 2=New HF)(2) | 2.588 | .469 | 30.411 | 1 | <.001 | 13.304 | 5.303 | 33.378 |
|  | In patient diuretic use(1) | 1.137 | .370 | 9.436 | 1 | .002 | 3.117 | 1.509 | 6.438 |
|  | Hyponatremia present up to 7 days of hospitalization Yes:1 No:0(1) | .735 | .369 | 3.964 | 1 | .046 | 2.086 | 1.012 | 4.302 |
|  | Constant | -.975 | .503 | 3.755 | 1 | .053 | .377 |  |  |
| Step 11^a^ | SEX Female: 1 Male:0(1) | -.930 | .356 | 6.834 | 1 | .009 | .395 | .196 | .792 |
|  | Current Smoker Yes:1 No: 0(1) | .511 | .401 | 1.619 | 1 | .203 | 1.666 | .759 | 3.659 |
|  | History of HTN Yes:1 No:0(1) | -1.372 | .452 | 9.220 | 1 | .002 | .253 | .105 | .615 |
|  | History of Stroke Yes:1 No:0(1) | -1.947 | .824 | 5.577 | 1 | .018 | .143 | .028 | .718 |
|  | History of Percutaneous Coronary Intervention Yes:1 No:0(1) | .635 | .400 | 2.516 | 1 | .113 | 1.887 | .861 | 4.134 |
|  | Heart failure diagnosis (0=No HF, 1= History of HF, 2=New HF) |  |  | 31.030 | 2 | <.001 |  |  |  |
|  | Heart failure diagnosis (0=No HF, 1= History of HF, 2=New HF)(1) | .836 | .433 | 3.721 | 1 | .054 | 2.307 | .987 | 5.393 |
|  | Heart failure diagnosis (0=No HF, 1= History of HF, 2=New HF)(2) | 2.616 | .470 | 30.922 | 1 | <.001 | 13.677 | 5.440 | 34.386 |
|  | In patient diuretic use(1) | 1.159 | .369 | 9.895 | 1 | .002 | 3.188 | 1.548 | 6.564 |
|  | Hyponatremia present up to 7 days of hospitalization Yes:1 No:0(1) | .702 | .365 | 3.694 | 1 | .055 | 2.018 | .986 | 4.128 |
|  | Constant | -.842 | .486 | 3.002 | 1 | .083 | .431 |  |  |
| Step 12^a^ | SEX Female: 1 Male:0(1) | -.899 | .354 | 6.465 | 1 | .011 | .407 | .204 | .814 |
|  | History of HTN Yes:1 No:0(1) | -1.449 | .445 | 10.605 | 1 | .001 | .235 | .098 | .562 |
|  | History of Stroke Yes:1 No:0(1) | -1.929 | .809 | 5.683 | 1 | .017 | .145 | .030 | .710 |
|  | History of Percutaneous Coronary Intervention Yes:1 No:0(1) | .660 | .401 | 2.704 | 1 | .100 | 1.934 | .881 | 4.246 |
|  | Heart failure diagnosis (0=No HF, 1= History of HF, 2=New HF) |  |  | 30.844 | 2 | <.001 |  |  |  |
|  | Heart failure diagnosis (0=No HF, 1= History of HF, 2=New HF)(1) | .784 | .428 | 3.350 | 1 | .067 | 2.190 | .946 | 5.070 |
|  | Heart failure diagnosis (0=No HF, 1= History of HF, 2=New HF)(2) | 2.584 | .466 | 30.781 | 1 | <.001 | 13.246 | 5.317 | 32.997 |
|  | In patient diuretic use(1) | 1.102 | .363 | 9.237 | 1 | .002 | 3.011 | 1.479 | 6.128 |
|  | Hyponatremia present up to 7 days of hospitalization Yes:1 No:0(1) | .749 | .362 | 4.276 | 1 | .039 | 2.115 | 1.040 | 4.300 |
|  | Constant | -.654 | .456 | 2.054 | 1 | .152 | .520 |  |  |
| a. Variable(s) entered on step 1: SEX Female: 1 Male:0, Age 1<65 2:≥ 65, Current Smoker Yes:1 No: 0, History of HTN Yes:1 No:0, History of Diabetes Yes:1 No:0, History of Stroke Yes:1 No:0, History of Transient Ischemic Attack Yes:1 No:0, History of Peripheral Vascular Disease Yes:1 No:0, History of Cancer Yes:1 No:0, History of Myocardial Infarction Yes:1 No:0, History of Percutaneous Coronary Intervention Yes:1 No:0, History of CABG Yes:1 No:0, History of CKD Yes:1 No:0, Taking ACEi, ARNi, or ARB Yes:1 No:0, Taking Diuretics Yes:1 No:0, Heart failure diagnosis (0=No HF, 1= History of HF, 2=New HF), In patient diuretic use, Hyponatremia present up to 7 days of hospitalization Yes:1 No:0. | | | | | | | | | |
